# Lunar‐Based Photothermal CO_2_ Reduction Strategy: Self‐Evolving Transient Active Interface and Band Engineering

**DOI:** 10.1002/advs.75487

**Published:** 2026-05-06

**Authors:** Yahang Wang, Yuhuan Li, Quanxin Wang, Deng Li, Pakkin Leong, Meng Wang, Shi Feng, Runyang Mo, Xianjin Shi, Xiaohui Li, Gangqiang Zhu, Chipui Tang

**Affiliations:** ^1^ State Key Laboratory of Lunar and Planetary Sciences Macau University of Science and Technology Taipa Macao P. R. China; ^2^ School of Instrument Science and Opto‐Electronics Engineering Beijing Information Science and Technology University Beijing China; ^3^ School of Physics and Information Technology Shaanxi Normal University Xi'an P. R. China; ^4^ School of Materials Science and Engineering Shaanxi Normal University Xi'an Shaanxi China; ^5^ State Key Lab of Loess and Quaternary Geology (SKLLQG) Institute of Earth Environment Chinese Academy of Sciences Xi'an P. R. China; ^6^ Faculty of Innovation Engineering Macau University of Science and Technology Taipa Macao P. R. China

**Keywords:** d‐d transition, Ilmenite (FeTiO_3_), lunar in situ resource utilization, self‐evolutionary, transient active interface

## Abstract

Inspired by lunar soil composition, we constructed a dynamically adaptive Pd/Ov‐FeTiO_3_ photothermal catalyst for in situ resource utilization. Under photothermal conditions, the catalyst undergoes significant self‐evolution, forming a transient active interface rich in oxygen vacancies (Ov) and palladium. This unique structure effectively enhances the adsorption of CO_2_ and key intermediates by shifting the d‐band center upward, while reconstructing the interfacial electron transport pathway, enabling rapid transfer of photogenerated electrons to the reactants. Furthermore, Ov‐induced d‐d transitions successfully release the energy of infrared photons, extending the photoresponse range to the near‐infrared region and significantly improving the full‐spectrum utilization efficiency of sunlight. In the photothermal CO_2_ hydrogenation reaction at 300°C, the optimized catalyst exhibits a CO generation rate of 33.23 mol g_Pd_^−^^1^ h^−^^1^, maintaining excellent performance in the visible‐infrared region. By combining femtosecond transient absorption spectroscopy, in situ XPS, in situ EPR, and DFT calculations, this study reveals the core role of transient active interfaces in promoting charge separation, lowering reaction energy barriers, and guiding reaction pathways. It not only proposes a highly efficient photothermal catalytic material design strategy based on a “self‐evolution” mechanism but also provides a practical pathway for extraterrestrial artificial photosynthesis, marking a crucial step toward practical application.

## Graphical abstract

A self‐evolving Pd/Ov‐FeTiO_3_ photothermal catalyst, inspired by lunar soil, enables efficient extraterrestrial CO_2_ conversion. The formation of transient active interfaces and oxygen vacancies extends light absorption to the infrared range and optimizes electron transfer. Achieving high CO generation rates, this work provides a robust strategy for sustainable deep‐space resource utilization through artificial photosynthesis.

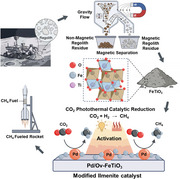

## 1 Introduction

The exploration of the Moon, representing humanity's aspiration to expand its living space, has emerged as a strategic frontier in deep‐space missions for both China and the United States, with lunar bases anticipated to be established around 2030 [1, 2, 3]. In situ utilization of lunar resources is essential to support astronauts’ life support systems and fundamental scientific investigations, thereby drawing substantial attention from the research community. Previous studies have shown that carbon dioxide and water ice exist in lunar cold traps [4, 5, 6, 7, 8]. Moreover, carbon dioxide generated from astronauts’ daily activities and plant respiration is considered a valuable resource on the resource‐limited Moon. Therefore, in situ hydrogenation of carbon dioxide on the Moon not only provides a means for hydrogen storage but also enables the conversion of CO_2_ into basic chemicals essential for routine scientific experiments (Figure S1) [9, 10].

**FIGURE 1:**
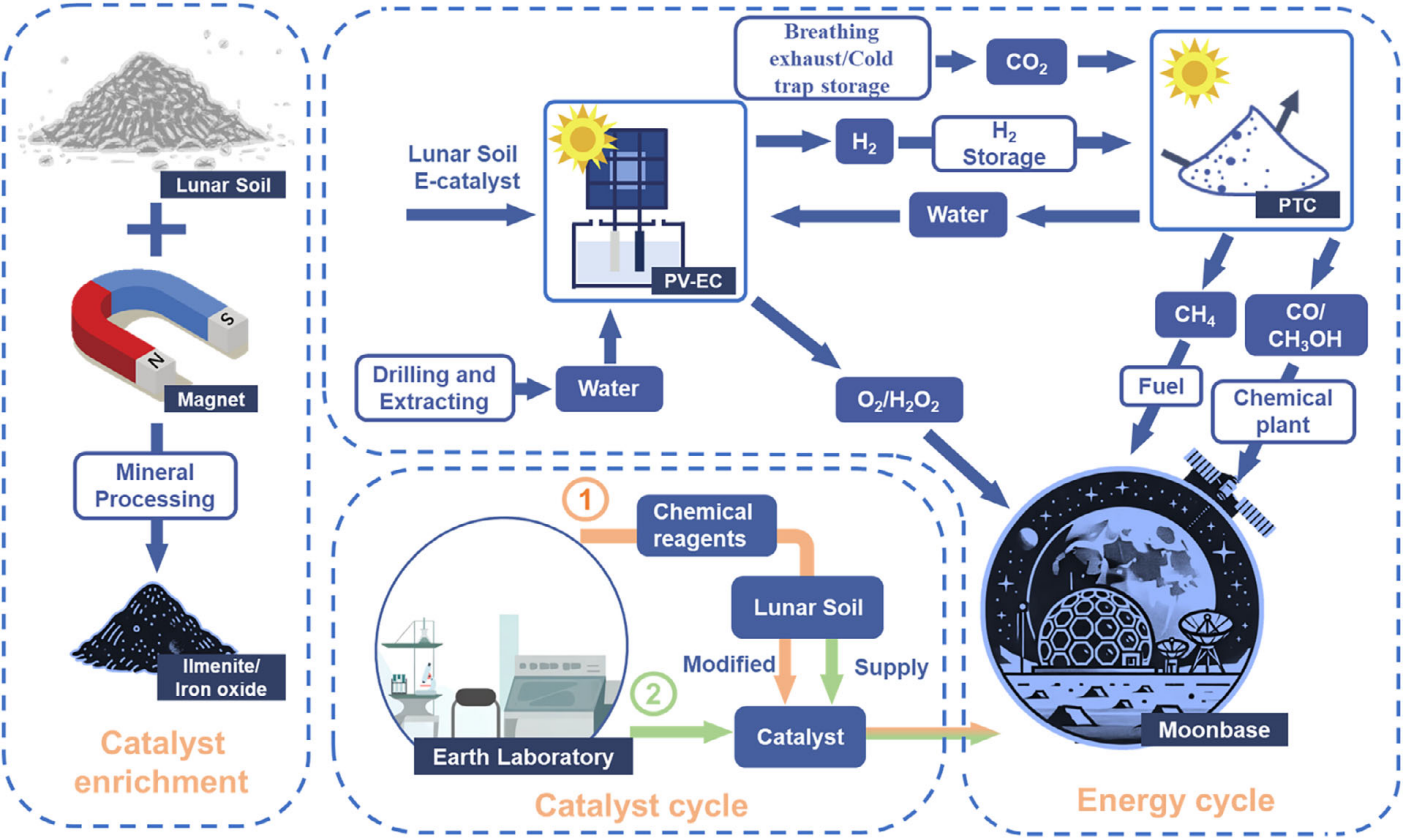
In situ resource utilization plan for the moon. Diagram of the energy cycle and catalyst cycle of the lunar base.

To establish a sustainable power supply on the Moon via carbon dioxide hydrogenation, it is essential to integrate both catalyst and energy cycles (Figure 1). In the energy cycle, abundant solar irradiation plays a dual role by driving water splitting to produce H_2_ and providing thermal energy (Figure S2). This synergistic mechanism makes photothermal CO_2_ hydrogenation a promising and viable strategy to address both energy and material demands. For the catalyst cycle, two strategic pathways are proposed. The first strategy involves fabricating high‐efficiency catalysts on Earth, followed by in situ replenishment of the consumed catalysts in lunar facilities using lunar soil. The second strategy entails direct in situ modification of lunar soil using chemical reagents delivered from Earth. Consequently, utilizing lunar soil to synthesize CO_2_ hydrogenation catalysts emerges as a crucial requirement for this integrated system.

Ilmenite (FeTiO_3_), a perovskite‐structured material abundant in lunar soil, exhibits excellent stability and a narrow band gap. It possesses high‐density hydrogen adsorption sites formed through solar wind implantation, along with excellent photothermal catalytic properties [11, 12, 13]. It is worth noting that ilmenite exhibits weak ferromagnetism and inherent electrical conductivity, which facilitate its efficient and low‐energy separation and enrichment under water‐scarce and low‐gravity conditions, Table S1 [14, 15]. Leveraging these unique characteristics, lunar soil‐derived ilmenite emerges as a promising material for photothermal CO_2_ hydrogenation on the Moon.

However, the relatively stable surface of natural ilmenite limits its performance [16]. Although the introduction of co‐catalysts (such as noble metal support) is a recognized effective activation strategy, current mechanisms are often limited to the “initial static structure” of the catalyst [17, 18]. In fact, under the strong field environment of photothermal catalysis, the catalyst surface—especially the metal‐support interface—is not static, but evolves into a thermodynamically metastable but highly active state. Therefore, simple loading is not the end point; the key is to reveal and utilize this loading‐induced “self‐evolution” process: that is, how to transform the static precursor into a transient active interface in the reaction.

This study achieved a breakthrough in photothermal catalysis by constructing a Pd/Ov‐FeTiO_3_ catalyst with a transiently active interface. This interface self‐evolves under reaction conditions, enhancing adsorption through the upward shift of the d‐band center, promoting transport through a reconstructed electron transfer chain, and extending the photoresponse through d‐d transitions, ultimately synergistically driving highly efficient CO_2_ conversion (CO yield as high as 33.23 mol g_Pd_^−^^1^·h^−^^1^). This work not only reveals the crucial role of the dynamic evolution of the interface but also provides a complete solution from material design to mechanistic innovation for in situ lunar resource utilization.

## 2 Results

### 2.1 Mineral Screening and Modification

**FIGURE 2:**
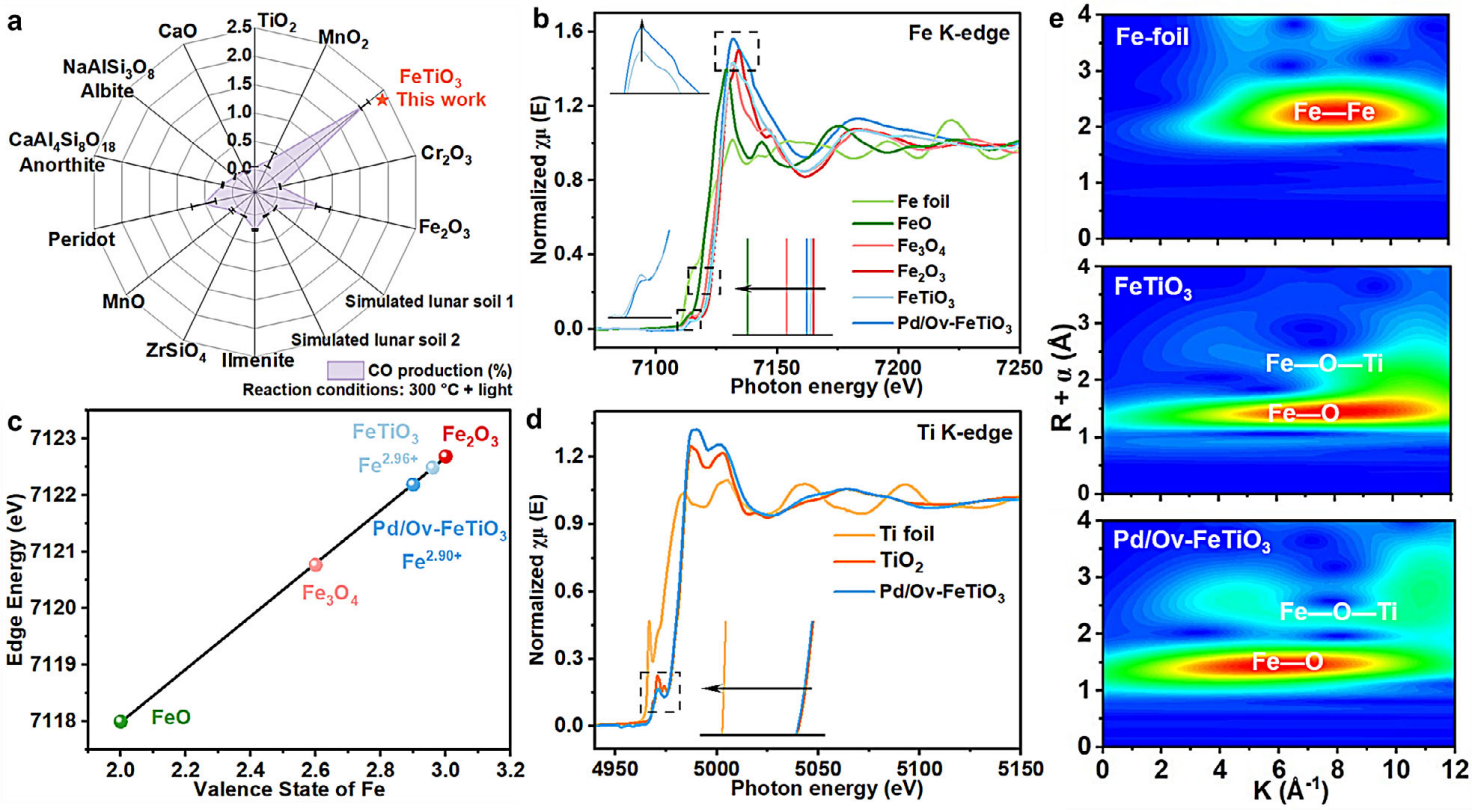
Catalyst Screening and Structural Characterization. (a) Comparison of catalyst performances formed by common minerals in lunar soil. (b) Fe K‐edge XANES spectrum, with the inset showing the pre‐edge, edge, and white‐line. (c) The oxidation states of the upper Fe in different samples were calculated via Fe K‐edge XAFS. (d) Ti K‐edge XANES spectrum, with the inset showing the edge. (e) WT of Fe foil FeTiO_3_ and Pd/Ov‐FeTiO_3_.

Considering the presence of various minerals in lunar soil [4], including TiO_2_, MnO_2_, ZrSiO_4_, Fe_2_O_3_, and FeTiO_3_, we initially screened these candidates for photothermal CO_2_ hydrogenation. A customized photothermal system, in which the catalyst bed was simultaneously heated and irradiated, was employed to evaluate the catalytic activities of these minerals (Figure S3). CO and CH_4_ were detected as the reduction products using these lunar minerals, as well as two simulated lunar soil samples provided by the Chinese Academy of Sciences. The photothermal CO_2_ hydrogenation performances of these common lunar minerals and the two simulated lunar soils were then systematically compared (Figure 2a). The results reveal that ilmenite exhibits superior photothermal catalytic activity, highlighting its potential for CO_2_ hydrogenation on the lunar surface. In addition, we evaluated the feasibility of enriching ilmenite, and the results confirmed its easy separation via magnetic mineral treatment (Figure S4).

We also observed that Fe_2_O_3_, another mineral present in lunar soil, exhibited relatively good activity for photothermal CO_2_ hydrogenation. However, Fe_2_O_3_ exhibited poor stability during continuous photothermal operation, performing worse than FeTiO_3_ (Figure S5). Therefore, FeTiO_3_ was identified as the most promising candidate in lunar soil for photothermal CO_2_ reduction on the Moon.

Continuous solar wind irradiation alters the properties of ilmenite in lunar soil, making it distinct from its terrestrial counterpart (Figure S6). Specifically, solar wind implantation increases the specific surface area and enhances hydrophilicity.[7, 8] To simulate lunar ilmenite, the goal was to modify it to overcome its insufficient reactant activation, limited spectral utilization, and restricted carrier dynamics, thereby achieving a higher CO_2_ reduction yield. Therefore, a high‐surface‐area FeTiO_3_ was synthesized via a sol‐gel method (Figures S7 and S8), and Pd and oxygen vacancies(Ov) were introduced onto its surface for modification. XRD and fourier transform infrared (FTIR) spectroscopy confirmed the successful synthesis, and the modified FeTiO_3_ retained its hexagonal structure (Figures S9 and S10). Compared to commercial ilmenite, the sol‐gel synthesized FeTiO_3_ exhibited a significantly larger surface area, as confirmed by N_2_ adsorption‐desorption isotherms (Figure S8). Photothermal performance tests demonstrated that the sol‐gel‐prepared FeTiO_3_ exhibited significantly enhanced activity compared to its commercial counterpart (Figure 2a). The sample morphology did not change significantly before and after Pd loading (Figure S11). TEM elemental mapping revealed the co‐localization of Pd, Fe, O, and Ti, indicating that the Pd cocatalyst was uniformly distributed on the surface (Figure S12).

To further evaluate the surface chemistry of FeTiO_3_ and compare it with ilmenite in lunar regolith, x‐ray photoelectron spectroscopy (XPS) was employed to investigate the surface properties of the Pd/Ov‐FeTiO_3_ catalyst. An interesting phenomenon was observed in the Fe 2p and Ti 2p spectra, revealing the coexistence of two oxidation states, Fe^2^^+^/Fe^3^^+^ and Ti^4^^+^/Ti^3^^+^, in FeTiO_3_ (Figure S13a,b, and Table S2). Pd was introduced via photoirradiation, combined with a reduction process similar to that of ilmenite in lunar soil reduced by solar wind, resulting in an enhancement of the Ov peak in the O 1s spectrum (Figure S13d) [19, 20]. Electron paramagnetic resonance (EPR) spectroscopy also confirms this conclusion, as shown in Figure S14. The introduction of surface Ov also increased the surface electron concentration. The Pd 3d spectrum (Figure S13c) shows peaks corresponding to metallic Pd^0^ and Pd^2^^+^ species, confirming the successful deposition of Pd.

The local atomic structure of the catalyst was further investigated using x‐ray absorption fine structure (XAFS) spectroscopy. Figure 2b presents the Fe K‐edge x‐ray absorption near‐edge structure (XANES) spectra. The coordination environment of Fe in FeTiO_3_ is mainly defined by Ti–O octahedra, and the pre‐edge features suggest that the octahedral framework remains intact after the introduction of Ov and Pd [21, 22]. The white line intensity corresponds to the Fe 1s→4p electronic transition, while the peak width reflects the splitting of the 4p orbital, which is associated with intermediate species adsorption [23]. The Pd/Ov‐FeTiO_3_ sample exhibits a higher white line intensity and broader peak width. This suggests that Pd/Ov‐FeTiO_3_ possesses a greater number of unsaturated Fe sites. According to the linear correlation between edge energy and oxidation state, a redshift of the absorption edge in Pd/Ov‐FeTiO_3_ indicates a reduction in Fe valence compared to FeTiO_3_ [24]. Quantitative analysis shows that the Fe oxidation states in FeTiO_3_ and Pd/Ov‐FeTiO_3_ are +2.96 and +2.90, respectively (Figure 2c). A similar analysis of Ti revealed a valence state slightly lower than +4 (Figure 2d; Figure S15). Extended x‐ray absorption fine structure (EXAFS) spectra were Fourier‐transformed into R‐space to investigate Fe local coordination (Figures S16–S18). Fe foil and various Fe oxides were used as references, with Fe foil exhibiting only Fe─Fe bonds, consistent with previous literature [25]. FeTiO_3_, Pd/Ov‐FeTiO_3_, and other Fe oxides exhibited Fe–O coordination in the first shell, confirming the presence of Fe─O bonds in all Fe‐containing samples. In the second coordination shell, FeO, Fe_2_O_3_, and Fe_3_O_4_ showed characteristic Fe–O–Fe interactions. Wavelet transform (WT) analysis (Figure 2e; Figure S19 and Tables S3 and S4) revealed that the FeO─Ti bond appears at a lower R value, attributed to the slightly smaller ionic radius of Ti compared to Fe. These findings confirm the successful synthesis of FeTiO_3_ and demonstrate that its coordination environment remains largely unaltered after the introduction of Pd and Ov.

### 2.2 “Self‐Evolutionary” Transient Active Interface and d‐d Transition

**FIGURE 3:**
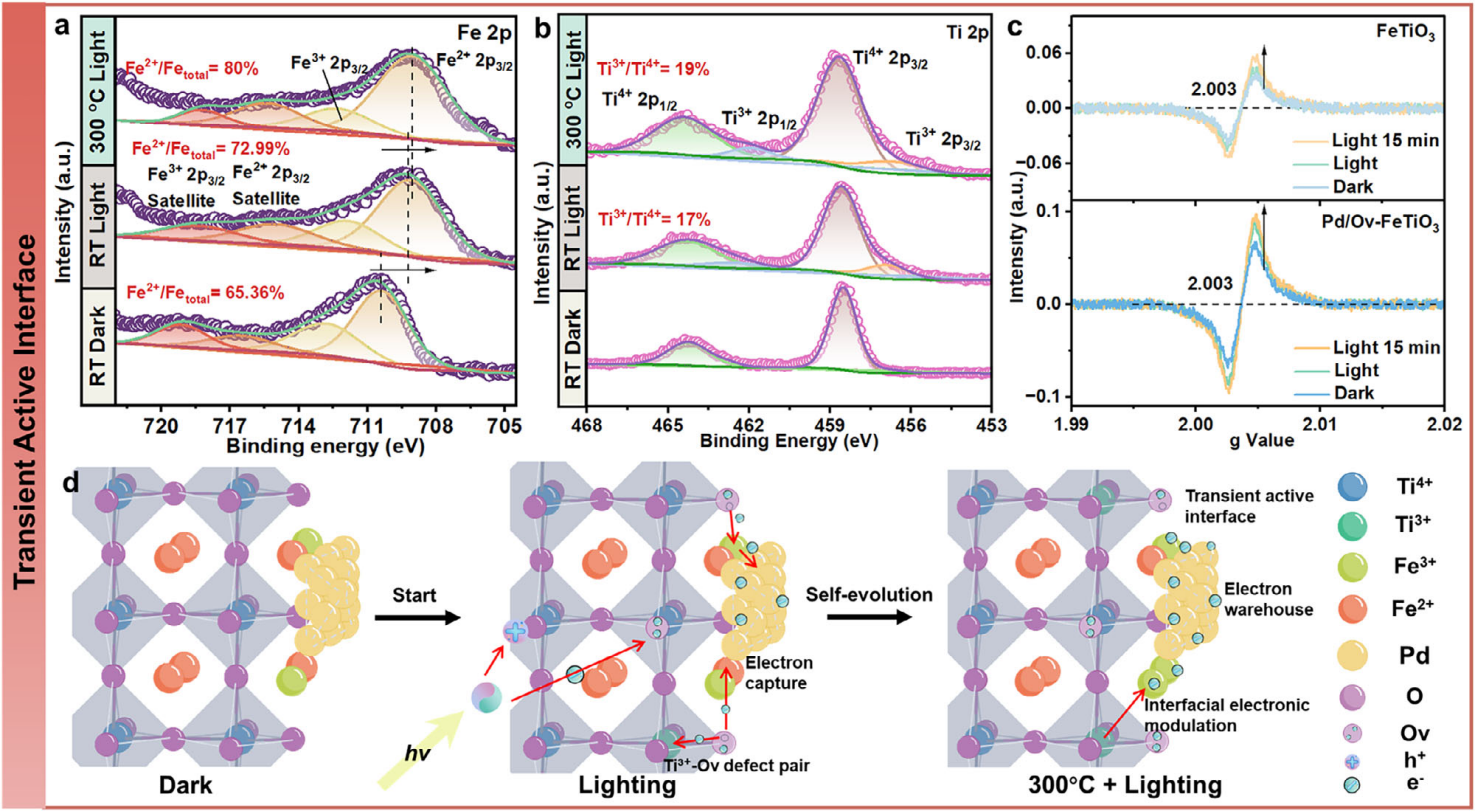
“Self‐evolution” transient active interface. High‐resolution in situ XPS spectra of Pd/Ov‐FeTiO_3_:Fe 2p (a), and Ti 2p (b). (c) In situ EPR spectrum of FeTiO_3_ and Pd/Ov‐FeTiO_3_. (d) Transient active interface schematic diagram.

Traditionally, the understanding of the structure‐activity relationship of catalysts is largely based on their initial surface chemical state, which is often considered static. However, the surface chemical state of catalysts evolves dynamically in real reaction environments [26, 27]. To further investigate the surface reconstruction behavior of Pd/Ov‐FeTiO_3_ in photothermal catalysis, we used in situ XPS and in situ EPR to track its chemical state in real time during the “dark state → light exposure → photothermal” process. The in situ XPS results clearly revealed a self‐evolutionary process triggered by Ov. As shown in Figure S20a and Table S5, intrinsic Ov exist in O 1s, and their proportion increases significantly after light exposure, further increasing under photothermal conditions. This phenomenon indicates that Ov is not only an electron‐capturing center, but its continuous generation provides a continuous driving force for subsequent surface reconstruction, which can be regarded as the “beginning” of the self‐evolutionary process. Fe 2p behaves similarly to Pd 3d. After light exposure, the binding energies of Fe and Pd both shift significantly to lower energies, and the proportion of their low‐valence species increases significantly (Figure 3a; Figure S20b). This means that photogenerated electrons are preferentially and abundantly captured, and Fe sites and Pd particles evolve into electron buffers (transient Electron Buffering and Transfer) and electron warehouse (interfacial localized electron density enrichment zones), respectively, entering an electron‐rich state. In subsequent photothermal processes, the magnitude of the binding energy shift for both decreases, and the increase in the proportion of low‐valence states slows, suggesting that their reduced states tend to saturate, reaching a dynamic equilibrium. Most interestingly, Ti evolves. In the dark, almost no Ti^3^^+^ species exist, but after illumination, a large number of Ti^3^^+^‐Ov defect pairs are generated. However, unlike Fe and Pd, the overall binding energy of Ti does not shift (Figure 3b). This seemingly contradictory phenomenon reveals a subtle balance: the increase in Ti^3^^+^ itself lowers the binding energy, but as an electron‐deficient activation site, it is subject to interfacial electron regulation by the neighboring electron‐rich Pd state, resulting in a slight electron withdrawal. The two effects cancel each other out, ultimately leading to an unchanged apparent binding energy. In situ EPR results further confirm the interfacial electronic evolution characteristics revealed by in situ XPS (Figure 2c). Unlike the linear enhancement of the Ov‐related paramagnetic signal in FeTiO_3_, Pd/Ov‐FeTiO_3_ exhibits a distinct “fast at first, slow later” evolution behavior. The rapid initial growth corresponds to the rapid activation of the interface under illumination, while the slowing growth in the later stage indicates that newly generated electrons are rapidly extracted and stored by neighboring Fe and Pd sites, rather than continuously accumulating at the Ov‐related centers. This suggests that the modification of FeTiO_3_ reconstructs the evolution path of Ov‐related electrons under illumination, accelerates the redistribution of interfacial electrons, and promotes the system to reach a steady state more quickly. In summary, these dynamic changes all point to one conclusion: photothermal conditions trigger a transient active interface with diverse functions, starting from Ov, through electron capture and redistribution, and ultimately evolving (Figure 3d). This interface is synergistically composed of an electron‐rich Pd warehouse, reduced Fe as an electron buffer, and Ti^3^^+^‐Ov defect pairs as electron‐deficient activation sites, providing an ideal platform for achieving efficient catalysis.

**FIGURE 4:**
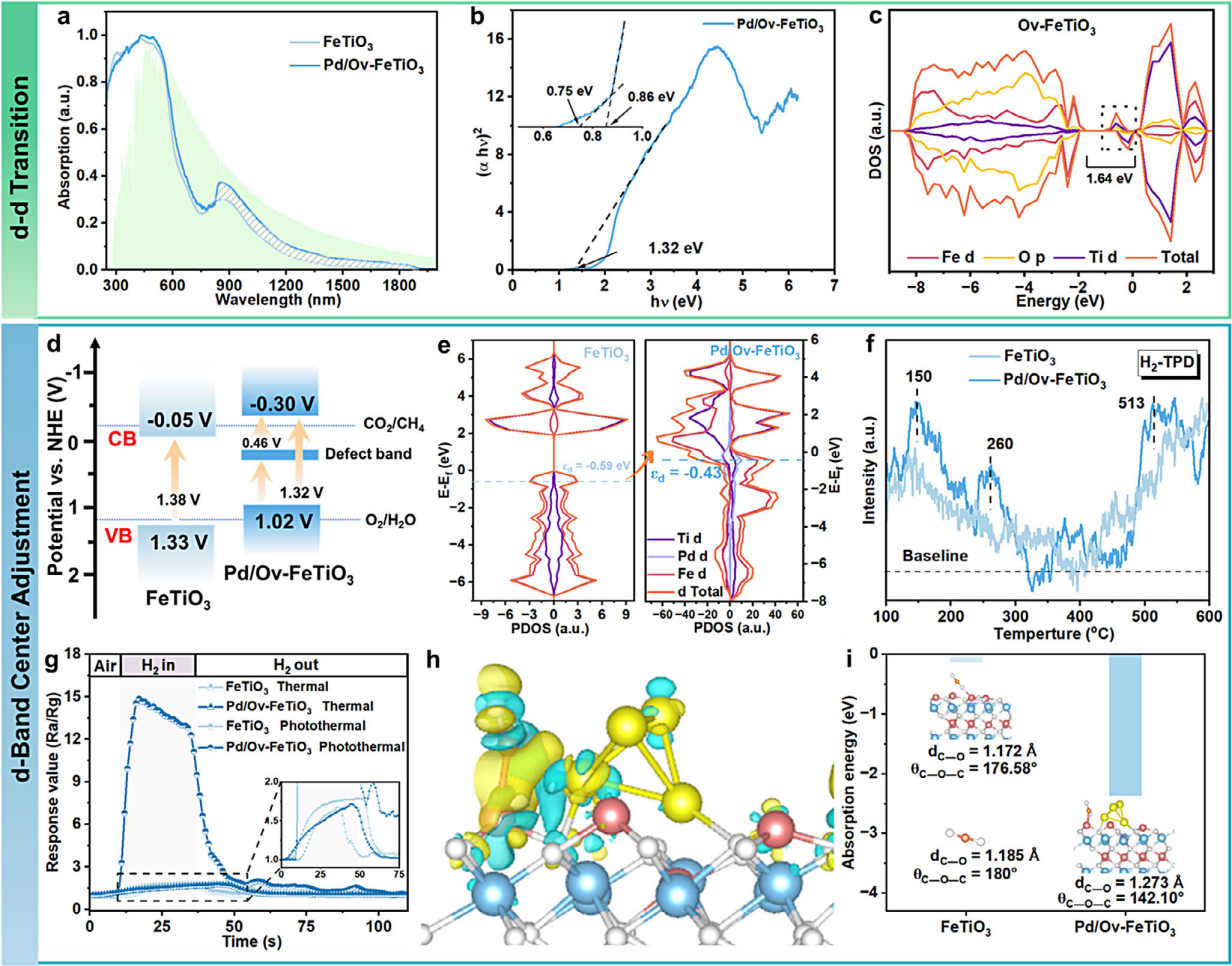
Band Structure Adjustment and its Effect on Adsorption Behavior. Optical absorption spectra of FeTiO_3_ and Pd/Ov‐FeTiO_3_ (a) and corresponding optical band gaps (b). (c) PDO diagram of Ov‐FeTiO_3_ (d) orbitals. (d) Energy band structure diagram after the introduction of Pd and Ov. (e) Diagram of the d‐band center before and after the introduction of Pd on the FeTiO_3_ surface. (f) H_2_ ‐ TPD. (g) In situ resistance response value test. (h), Differential charge density of CO_2_ adsorbed on FeTiO_3_ and Pd/Ov‐FeTiO_3_. (The yellow electron cloud surface electrons gather, and blue indicates electron dissipation. The colors of the spheres correspond to the elements: yellow (Pd), red (Fe), blue (Ti), white (O), and gray (C).) (i) Adsorption energies, bond lengths, and bond angles of FeTiO_3_ and Pd/Ov‐FeTiO_3_ for CO_2_.

The effects of Pd cocatalysts andOv on the optical properties of the catalyst were further investigated. As shown in the UV–Vis–NIR absorption spectrum (Figure 4a), the infrared absorption of FeTiO_3_ was significantly enhanced after the incorporation of Pd and surface Ov. A characteristic absorption edge near 750 nm was observed both before and after modification, corresponding to an optical band gap of approximately 1.3 eV for FeTiO_3_ (Figure 4b, Figure S21). Interestingly, the Tauc plot indicates that Pd/Ov‐FeTiO_3_ exhibits multiple apparent band gaps at 0.86 and 0.75 eV, suggesting the formation of intermediate energy states induced by surface modification [28, 29]. This observation aligns with previous reports indicating that intraband electron transitions enhance light absorption in the visible and near‐infrared regions. Projected density of states (PDOS) calculations were conducted on FeTiO_3_ before and after modification to identify the presence of intraband states (Figure 4c; Figure S22–S24). The results revealed that oxygen vacancy rich FeTiO_3_ exhibited defect states within the band gap, corroborating the experimental observations. In‐depth theoretical and symmetry analysis shows (Figure S25) that the local symmetry breaking induced by Ov and Pd nanoparticles effectively removes the Laporte forbidden effect through p‐d hybridization, making the nominally forbidden d‐d transitions partially allowed. Meanwhile, empty d orbitals were introduced into the band gap as an intermediate “buffer layer”. The above mechanisms together construct an efficient subbandgap excitation channel, which not only promotes gradual electronic transitions and cascade charge transfer, but also fundamentally and significantly enhances the infrared light response of the catalyst [30].

To determine the band structure of Pd/Ov‐FeTiO_3_, the valence band positions were measured using XPS valence band spectra (Figure S26), yielding values of 1.33 and 1.03 V (vs. NHE) for FeTiO_3_ and Pd/Ov‐FeTiO_3_, respectively. Based on these results, the band structure diagrams of FeTiO_3_ before and after modification were constructed, as shown in Figure 4d. Initially, pure FeTiO_3_ exhibits a conduction band minimum and valence band maximum at −0.05 and 1.33 eV, respectively. Upon palladium loading and the introduction of Ov, the valence band edge of Pd/Ov–FeTiO_3_ shifts upward to 1.02 eV, and an intra‐band energy level emerges at approximately 0.46 eV.

Furthermore, theoretical calculations further revealed the profound impact of surface reconstruction at the electronic structure level. DFT results showed that after undergoing self‐evolution, the d‐band center of the Pd active center in Pd/Ov‐FeTiO_3_ shifted significantly upward compared to the initial state and was closer to the Fermi level (Figure 4e) [31]. This key change mainly stemmed from two major effects in the transient active interface formed by self‐evolution: first, the electron‐rich Pd generated stronger electron‐electron repulsion due to the increased occupancy of d‐orbital electrons, leading to energy level expansion; secondly, the ligand and strain effects caused by Ov on the support surface and reduced Fe jointly modulated the electronic structure of Pd. According to the d‐band center theory, this upward shift meant that Pd's adsorption capacity for reactants and intermediates was significantly enhanced [30, 32]. At the same time, Ov induced defect states below the conduction band of FeTiO_3_, while significant orbital hybridization occurred at the interface between Pd and FeTiO_3_ (Figures S22–S25). The reconstruction of the electronic structure and the band modulation together promote the realization of the electron warehouse function and the increase of carrier concentration at the interface [31]. More importantly, the built‐in electric field formed at the interface from FeTiO_3_ to Pd (Figures S27 and S28) greatly optimizes the directional migration efficiency of photogenerated electrons from FeTiO_3_ to the Pd active center, providing a kinetic basis for the rapid catalytic cycle on the transient active interface.

To further demonstrate the impact of the upward shift of the d‐band center and the transient active interface on electron transfer, characterization and theoretical calculations were performed. Hydrogen‐programmed temperature desorption (H_2_‐TPD) analysis showed that Pd/Ov‐FeTiO_3_ exhibited more desorption peaks and larger peak areas, indicating that the introduction of Pd and Ov provided additional active sites for H_2_ adsorption and dissociation, and enhanced the binding affinity of H_2_ (Figure 4f) [33, 34]. The enhanced H_2_ adsorption was attributed to the upward shift of the d‐band center. To elucidate the electron transfer behavior of under different reaction conditions after the introduction of Pd/Ov‐, in situ resistance response measurements were performed on the samples (Figure 4g). The increase in resistance response indicated enhanced electron transfer activity. Under photothermal conditions, Pd/Ov‐FeTiO_3_ exhibited the largest resistance change. This demonstrates that the transient active interface formed through “self‐evolution” possesses a unique catalytic microenvironment composed of electron‐deficient Ti^3^^+^‐Ov sites and electron‐rich Pd. Pd, acting as an “electron pool,” not only provides locally high‐density electronic states but also serves as a continuous dynamic charge hub, continuously pumping captured photogenerated electrons into the antibonding orbitals of the H─H σ bond, thus significantly improving the activation efficiency of the H_2_ molecule. The interfacial electron transport kinetics of the transient active interface formed through “self‐evolution” are significantly improved.

Differential charge density analysis (Figure 4h; Figures S29 and S30) further confirms the interfacial electron transfer behavior during the self‐evolution process from a theoretical perspective. On the FeTiO_3_ surface, electrons accumulate at CO_2_ adsorption sites, and electrons are simultaneously enriched around Fe sites, which is consistent with our earlier hypothesized interfacial electron transport pathway from Ti^3^^+^‐Ov to Fe. In the Pd/Ov‐FeTiO_3_ system, both CO_2_ and CO molecules are stably adsorbed on the transient active interface formed by Pd and FeTiO_3_, indicating that the introduction of Pd significantly enhances the ability of electrons to transfer to reactant molecules at the interface. This phenomenon originates from a synergistic microenvironment formed through self‐evolution: the electron‐rich Pd pool enhances the adsorption of reactant molecules by shifting the d‐band center upward, while Ov and the electron‐deficient activation sites (Ti^3^^+^‐Ov) induced by them jointly construct a highly efficient electron transfer channel. Therefore, compared with pure FeTiO_3_, Pd/Ov‐FeTiO_3_ exhibits significant advantages in the adsorption, activation, and electron transfer capabilities of reactant gas molecules (Figure 4i; Figure S31), fully revealing the microscopic mechanism by which the transient active interface enhances catalytic performance at the electronic scale.

### 2.3 CO_2_ Hydrogenation Performance

**FIGURE 5:**
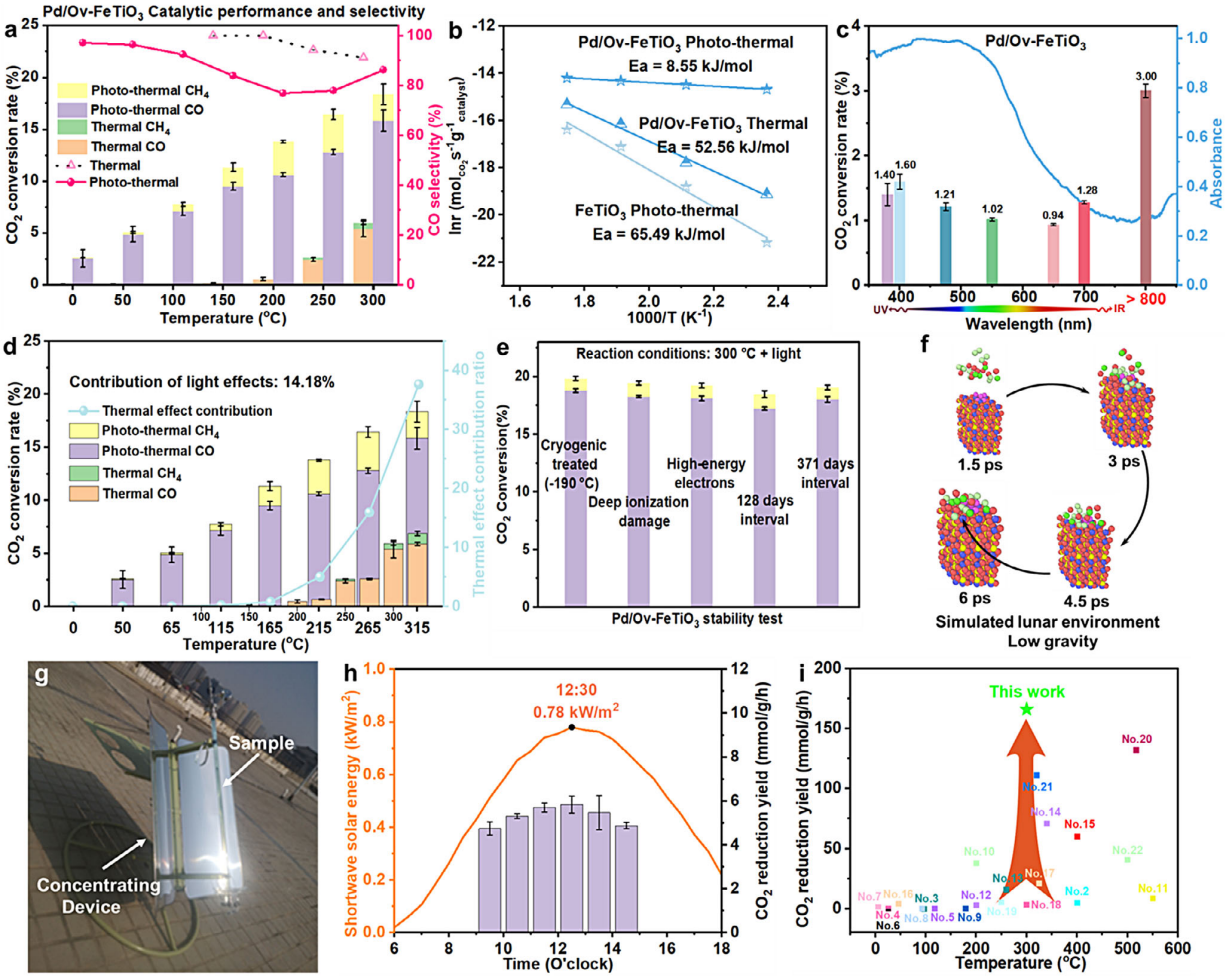
CO_2_ photothermal reduction performance. (a) Pd/Ov‐FeTiO_3_ photothermal CO_2_ hydrogenation performance and selectivity for CO (test conditions: 0°C–300°C, 15 mW cm^−2^). (b) Ea activation energy fitting diagram. (c) The performance of Pd/Ov‐FeTiO_3_ under monochromatic light and the superposition of UV–vis–NIR absorption spectra. (d). The contribution of thermal factors to the performance of Pd/Ov‐FeTiO_3_ under different temperature conditions. (e) Stability tests of Pd/Ov‐FeTiO_3_ under various conditions. (f) MD simulation of Pd/Ov‐FeTiO_3_.(The purple, blue, red, yellow, green, and light green spheres represent the elements Pd, Ti, O, Fe, C, and H, respectively.) (g) Outdoor CO_2_ reduction concentrating equipment. (h) Outdoor CO_2_ photothermal reduction performance. (i) Comparison of efficiency between this work and other works.

The photothermal CO_2_ hydrogenation performance of the catalyst before and after the introduction of d‐d transitions and the transient active interface was then evaluated. Figure 5a and Figure S32 illustrate the catalytic performance under photothermal CO_2_ hydrogenation conditions. The pristine FeTiO_3_ showed almost no catalytic activity at temperatures ≤150°C. At 300°C, the CO_2_ conversion reached 2%, and the CO selectivity approached 100%. The modified Pd/Ov‐FeTiO_3_ exhibited measurable activity even under room temperature illumination. At 300°C, the CO_2_ conversion increased to ∼20%, approximately ten times that of the pristine FeTiO_3_. The Pd/Ov‐FeTiO_3_ catalyst possesses more electrons in the CO_2_ reduction reaction, which indirectly contributes to the success of the transient active interface. Simultaneously, the CO selectivity reached 86%. With increasing temperature, the CO selectivity of the Pd/Ov‐FeTiO_3_ catalyst decreased, and the CH_4_ yield also decreased at 300°C. This indicates that the formation of CH_4_ (8 electronic processes) is thermodynamically more favorable at moderate temperatures; however, since the CO_2_ methanation reaction is exothermic, further increases in temperature lead to a decrease in the CH_4_ yield [9]. By adjusting the H_2_/CO_2_ ratio to 3:1, the CO_2_ conversion reached 31%, corresponding to a yield of approximately 166.16 mmol g^−^^1^·h^−^^1^ (Figure S33). Adjusting the Pd ratio can also adjust the CO selectivity, as shown in Figure S34. In addition, the Pd/Ov‐FeTiO_3_ catalyst exhibited high Pd utilization, with a yield of 33.23 mol·g_Pd_^−^^1^·h^−^^1^, indicating high catalytic efficiency. Increasing both the reaction temperature and the H_2_/CO_2_ ratio can improve the selectivity of CH_4_.

To elucidate the source of the enhanced activity of Pd/Ov‐FeTiO_3_, the activation energy (Ea) of the photothermal CO_2_ hydrogenation reaction of FeTiO_3_ and Pd/Ov‐FeTiO_3_ was first evaluated. The Ea values were determined by fitting reaction rate curves under different conditions (Figure 5b; Table S6). Under heating‐only conditions, the Ea of Pd/Ov‐FeTiO_3_ was 52.56 kJ mol^−^^1^, which significantly decreased to 8.55 kJ mol^−^^1^ after illumination, indicating that photothermal coupling significantly reduced the energy barrier. In contrast, FeTiO_3_ exhibited a higher Ea value of 65.49 kJ mol^−^^1^ under the same heating conditions, even higher than Pd/Ov‐FeTiO_3_ under no‐illumination conditions. These results demonstrate that coupling d‐d transitions with a transient active interface strategy can effectively reduce the activation energy of the reaction.

To further analyze the effect of modification on catalytic performance, the catalyst performance was tested under different optical bands (Figure 5c; Figure S35). The performance improvement observed in the ultraviolet and visible light regions was attributed to the success of the transient active interface strategy, which improved the adsorption and activation of reactants and intermediates. The performance improvement in the infrared region was attributed to the coupling of d‐d transitions with the transient active interface, which enhanced light absorption and enabled the utilization of infrared light [19, 28, 35].

To rule out the possibility that Pd or Ov alone could lead to enhanced activity, we prepared control catalysts by loading Pd onto ilmenite and SiO_2_ supports. Compared with Pd/Ov‐FeTiO_3_, these samples showed significantly reduced CO_2_ hydrogenation activity, confirming the synergistic effect between Pd loading and Ov (Figure S36) [19]. To elucidate the respective contributions of thermochemical and photochemical processes in CO_2_ hydrogenation, the photothermal catalytic behavior of Pd/Ov‐FeTiO_3_ was systematically analyzed (Figure 5d).

We independently quantified the thermal contribution of the photothermal effect. As the reaction temperature increased, the thermochemical contribution gradually dominated the entire process, indicating that the photothermal synergistic effect was limited at higher temperatures. Therefore, the photothermal synergistic effect was most significant in the temperature range of 150°C–250°C. Meanwhile, the number of electrons transferred by the Pd/Ov‐FeTiO_3_ catalyst is proportional to the light intensity, further indicating that the photoinduced carrier process plays an important role in advancing the reaction in this temperature range, as shown in Figure S37b. This observation is consistent with the temperature‐dependent CO selectivity exhibited by Pd/Ov‐FeTiO_3_, providing valuable guidance for the selection of product pathways in future lunar base applications.

We also evaluated the long‐term stability of Pd/Ov‐FeTiO_3_ under continuous photothermal CO_2_ hydrogenation conditions. As shown in Figures S38 and S39, the Pd/Ov‐FeTiO_3_ catalyst continuously generated CO and CH_4_ over 40 h without a significant decrease in catalytic activity, and the CO selectivity remained stable at 86% throughout the test. Considering the extremely low temperatures on the lunar surface (down to −170°C), no performance degradation was observed in the Pd/Ov‐FeTiO_3_ catalyst after liquid nitrogen treatment (Figure 5e and Figures S40–S42). Furthermore, after deep ionizing radiation and high‐energy electron treatment (simulating a partial cosmic ray environment), the catalyst still showed no significant activity loss in performance evaluations four months and one year after synthesis (Figure 5e; Figures S43–S45). As shown in Figure S46, further analysis indicates that the possible influence mechanism of common impurities in lunar soil on the Pd/Ov‐FeTiO_3_ system has been systematically ruled out. The Moon's gravity is approximately one‐sixth that of Earth. To investigate the specific impact of this gravitational difference on the reaction process, we conducted molecular dynamics (MD) simulations. As shown in Figure 5f, under the simulated low‐gravity (weakly constrained) environment of the Moon, reactant gas molecules require approximately 6 ps to effectively accumulate on the catalyst surface; while under simulated Earth conditions, this process requires only about 4 ps (Figure S47). This comparison indicates that the low‐gravity environment of the Moon does indeed delay gas mass transfer and surface accumulation, thus affecting reaction kinetics. However, we hypothesize that this adverse effect caused by reduced gravity can likely be compensated for and overcome by appropriately increasing the operating pressure of the reaction system. This hypothesis and its application in engineering practice will be an important direction for our future in‐depth research. To verify the application of the Pd/Ov‐FeTiO_3_ catalyst, we used a concentrator to focus sunlight and tested its CO_2_ reduction performance (Figure 5g). The results show that its performance is excellent (Figure 5h). In summary, the Pd/Ov‐FeTiO_3_ catalyst exhibits excellent thermal and temporal stability and strong durability under simulated lunar conditions. Furthermore, Pd/Ov‐FeTiO_3_ demonstrates top‐tier CO_2_ photothermal reduction performance compared to similar reports (Figure 5i; Table S7); even in Pd‐containing catalyst systems, its performance remains superior (Figure S48 and Table S8).

### 2.4 Carrier Dynamics

**FIGURE 6:**
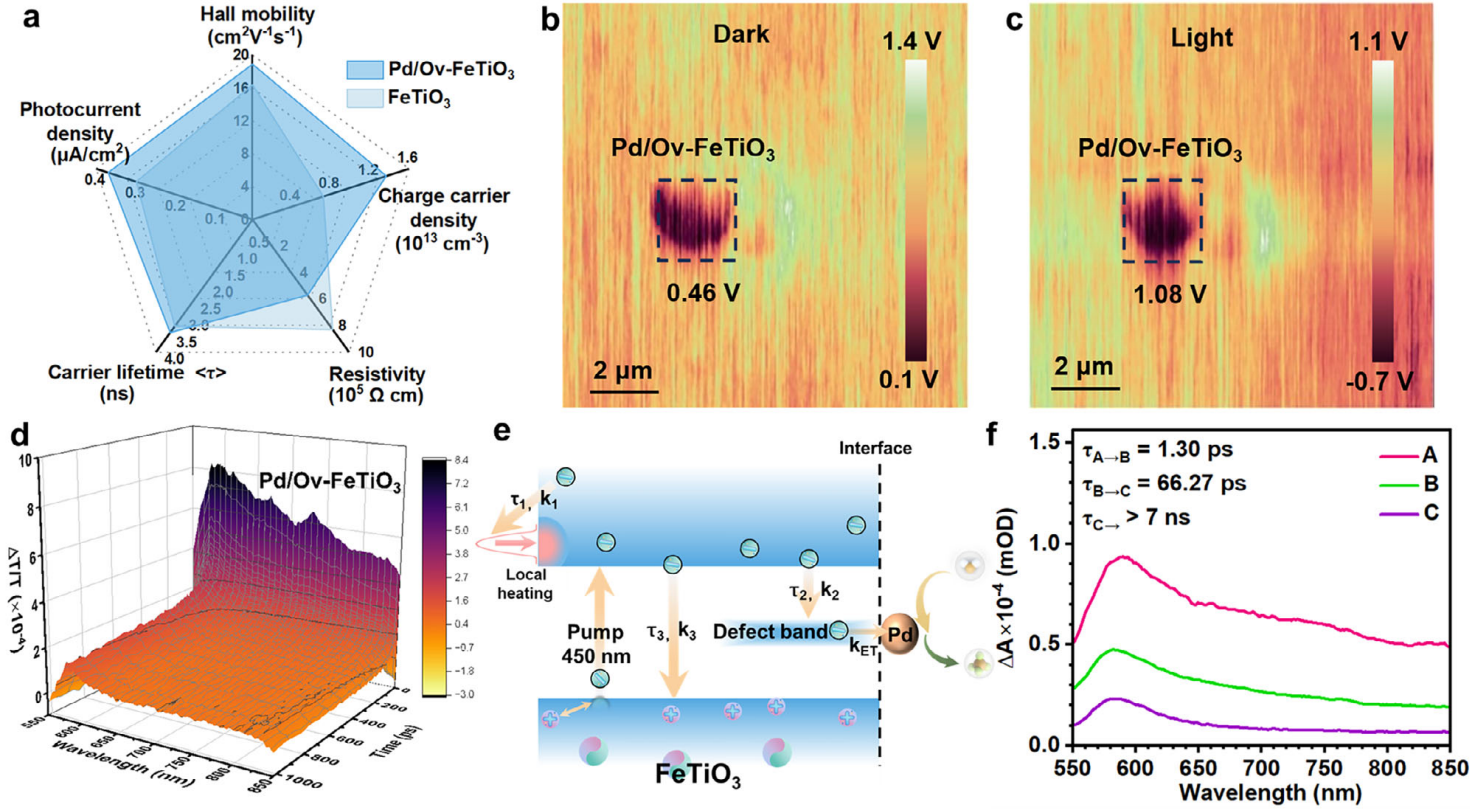
Carrier behavior. (a) Carrier concentration, lifetime, resistance, and photocurrent intensity of the sample. (b,c) KPFM images of FeTiO_3_ in the dark and under illumination. (d) fs‐TA spectra and (f) Globally fitted SAS and time constants of Pd/Ov‐FeTiO_3_. (e) a diagram of the kinetic process lifetime and charge transfer efficiency.

To further investigate the influence of transient active interfaces on carrier dynamics, carrier transport behavior was first evaluated. The carrier properties were comprehensively assessed using the Hall effect, transient fluorescence, and photocurrent measurements (Figure 6a; Figures S49 and S50 and Table S9). Due to the presence of the transient active interface, Pd/Ov‐FeTiO_3_ exhibited higher Hall mobility, higher carrier concentration, higher photocurrent density, lower resistivity, and longer electronic lifetime. Impedance spectroscopy further confirmed these phenomena (Figure S51). To investigate whether the introduction of transient active interfaces affects carrier excitation and migration behavior, we characterized the contact potential difference of different samples using light‐assisted Kelvin probe force microscopy (KPFM). As shown in Figure 6b,c, and Figure S52 the contact potential difference of Pd‐FeTiO_3_ was 49 mV under dark conditions, increasing to 102 mV under illumination. Compared with FeTiO_3_, illumination significantly increased the surface electron concentration of Pd/Ov‐FeTiO_3_, leading to a higher surface potential and a more significant potential change [36]. The above results illustrate that Ov play a dual role in the system: although they can act as exciton recombination centers, under photothermal coupling conditions, the generation of a large number of Ov triggers a self‐evolution process, thereby constructing an efficient transient active interface. This interface not only provides a higher surface electron concentration but also reconstructs the interface electron flow, enabling excited‐state electrons to bypass local defect states, be rapidly captured by the electron warehouse, and preferentially transferred to surface reaction sites, thus transforming the unfavorable recombination effect into an efficient electron utilization path.

To further understand the interface electron regulation of the transient active interface, femtosecond transient absorption spectroscopy was used to analyze the carrier dynamics in detail. Both FeTiO_3_ and Pd/Ov‐FeTiO_3_ showed strong excited‐state absorption (ESA) signals, and the specific properties of the ESA further confirmed that defect states dominate carrier dynamics (Figure 6d; Figure S53) [37, 38, 39]. This is consistent with the defect levels introduced by Pd and Ov. Detailed species‐associated spectra (SAS) analysis revealed a typical three‐step excited‐state dynamic: the initial ultrafast relaxation (τ_1_ ≈ a few picoseconds) dissipates energy through strong coupling with lattice phonons, representing the electron‐phonon energy conversion (Figure 6e). The subsequent intermediate process (τ_2_≈tens of picoseconds) corresponds to charge carriers being trapped by defect levels and migrating to the Pd interface, where they react with reactants; this stage is crucial for charge carrier diffusion and surface reactions. Finally, long‐lifetime relaxation (τ_3_≈hundreds of picoseconds) involves the relaxation of electrons trapped in deep traps or triplet states [35, 38]. Triplet states are not suitable for catalytic reactions due to spin forbidden and charge‐confined conditions. Figure 6f, Figures S53 and S54 show the global fit results of the transient absorption spectra of the two samples. The modified Pd/Ov‐FeTiO_3_ exhibits rapid cooling of hot electrons and a shortened electron lifetime in shallow trap levels in the SAS. Traditionally, this is often attributed to unfavorable energy dissipation and recombination. However, in this system, we interpret it as a manifestation of the ultra‐high efficiency of a “self‐evolving” transient active interface: hot electrons transfer energy to the lattice extremely quickly, driving photothermal reconstruction of the surface; while the shortened residence time of electrons in the shallow energy levels indicates that they are rapidly extracted by the electron‐rich Pd pool at the interface and transferred to the reactants, with the rate of consumption by the interface reaction far exceeding the rate of recombination or relaxation. Therefore, this “short lifetime” is essentially a kinetic characteristic of ultrafast interfacial electron transfer and transformation. Furthermore, the rate constant (k_2_) and charge transfer efficiency (η_et_) are used as quantitative indicators of this key process. Pd‐FeTiO_3_ exhibits a larger k_2_ value, indicating that this modification enhances the electron transfer efficiency within FeTiO_3_. The charge transfer efficiency η_et_ was estimated at 40.7% (Figure 6e), indicating that the Pd co‐catalyst enabled approximately half of the charge carriers to enter the reaction channel, effectively separating electrons. This interface, through the synergistic effect of its unique electron‐rich pool and electron‐deficient activation sites (Ti^3^^+^‐Ov), achieves rapid extraction, directional transport, and efficient utilization of photogenerated electrons. The “short lifetime” of electrons in the interface directly reflects the limiting kinetics of their rapid consumption by surface reactions, marking a shift from the metastable state of “waiting for recombination” in traditional semiconductors to a functional intermediate with “instantaneous use” in adaptive catalytic systems. This discovery demonstrates, on an ultrafast timescale, that the core of the self‐evolutionary process is the creation of an efficient quantum channel that precisely and directionally converts photothermal‐electrical energy into chemical energy.

### 2.5 Mechanism Analysis

**FIGURE 7:**
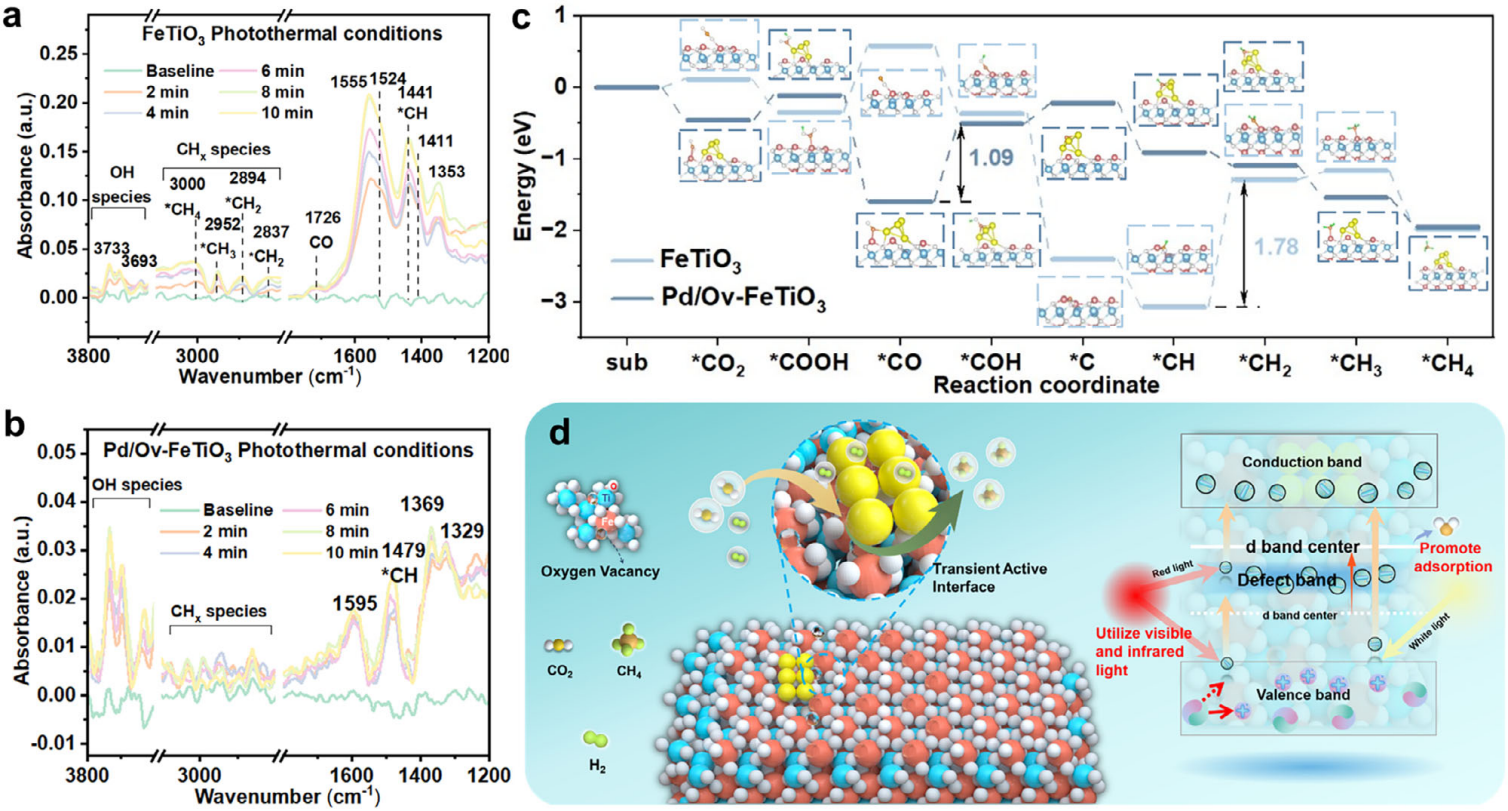
Reaction mechanism. In situ infrared spectrum of FeTiO_3_ (a) and Pd/Ov‐FeTiO_3_ (b) under photothermal conditions. (c) Reaction path diagram of the sample. (d) Reaction mechanism diagram.

To investigate the reaction process of CO_2_ hydrogenation reduction, in situ FTIR was used to detect reaction intermediates. Both samples exhibited characteristic absorption peaks near 3000 cm^−^^1^, attributed to CH_x_ intermediates (Table S10). Peaks at approximately 1450 and 1726 cm^−^^1^ corresponded to CH and CO species, respectively, while bands in the 3600–3800 cm^−^^1^ range were associated with surface OH species—key intermediates for the hydrogenation of CO_2_ to CH_4_ (Figure 7a,b; Figure S55) [40, 41, 42, 43]. Interestingly, some important intermediates gradually accumulated on FeTiO_3_ but not on Pd/Ov–FeTiO_3_, suggesting that the latter promotes faster reaction kinetics and more efficiently consumes adsorbed CO_2_ at equilibrium. Furthermore, the relatively strong OH absorption band of Pd/Ov–FeTiO_3_ indicates the formation of more OH and COOH‐containing intermediates on its surface, consistent with its higher reaction rate. In situ infrared spectroscopy confirmed the efficient electron transfer and strong activation of reactants by the transient active interface.

To verify the proposed photothermal CO_2_ reduction mechanism from theoretical calculations, DFT calculations were performed (Figure 7c; Figure S56 and S57). CO is a key intermediate, and its formation on FeTiO_3_ requires overcoming a certain energy barrier. For Pd/Ov‐FeTiO_3_, the rate‐determining step is the hydrogenation of ^*^CO to ^*^COH, with an energy barrier of 1.08 eV. Notably, the hydrogenation of ^*^C on Pd/Ov‐FeTiO_3_ is thermodynamically favorable, as evidenced by the reduced Gibbs free energy. In contrast, the rate‐determining step on FeTiO_3_ involves the hydrogenation of ^*^CH to ^*^CH_2_, which may hinder the conversion pathway from CO to CH_4_. This explains the high CO selectivity observed on FeTiO_3_. The lower rate‐determining step thermodynamic barrier indicates that CO_2_ activation and reduction are more favorable on Pd/Ov‐FeTiO_3_ than on FeTiO_3_. This advantage stems from the enhanced intermediate adsorption and activation resulting from the synergistic effect of the upward shift of the d‐band center in the self‐evolving interface and the interfacial electron flow. This mechanism not only promotes the efficiency of electron transfer to reactants but also crucially regulates the reaction pathway, thereby significantly enhancing the CO_2_ reduction activity and improving the CH_4_ selectivity.

Based on the above analysis, we propose a mechanism model for photothermal CO_2_ hydrogenation on the Pd/Ov‐FeTiO_3_ catalyst (Figure 7d). In this system, Ov introduce defect states in the semiconductor band gap, promoting d‐d electron transitions and significantly enhancing the material's spectral response and utilization efficiency in the infrared region. The introduction of Pd not only promotes the “self‐evolution” process on the catalyst surface and forms a transient active interface, but more importantly, it regulates the electronic structure of the active center, causing the d‐band center to shift upward, thereby enhancing the adsorption and activation ability of reaction intermediates and optimizing the interfacial electron transfer path.

## 3 Conclusion

This study focuses on in situ resource utilization on the moon, screening ilmenite, abundant in lunar soil, as a basic catalytic material. This mineral, due to its ease of extraction, abundant surface alkaline sites, structural stability, and suitable hydrogen adsorption capacity, shows great potential as a photothermal CO_2_ reduction catalyst for lunar bases. By introducing Pd and Ov, precise control of the electronic structure of FeTiO_3_ was achieved: the introduction of Pd promotes surface “self‐evolution,” forming a transient active interface, shifting the d‐band center upward, enhancing the adsorption of key intermediates, accelerating the electron redistribution and interface transfer process, thereby improving electron utilization efficiency; Ov introduce defect levels in the band gap, promoting d‐d transitions, expanding the infrared response, and simultaneously acting as local exciton recombination centers to improve photothermal conversion efficiency. Thanks to the synergistic effect of these multiple mechanisms, the modified catalyst exhibits excellent CO_2_ reduction performance under both infrared and full‐spectrum irradiation, with the highest CO yield reaching 33.23 mol g_Pd_^−^^1^ h^−^^1^. This study not only elucidates a highly efficient photothermal catalytic material based on lunar soil minerals, but also constructs a technical path of “lunar soil structure regulation—electronic behavior optimization—catalytic performance enhancement”, providing a promising new strategy for in situ conversion and resource recycling of CO_2_ in the lunar environment.

## Conflicts of Interest

The authors declare no conflicts of interest.

## Supporting information




**Supporting File**: advs75487‐sup‐0001‐SuppMat.docx.

## Data Availability

The data that support the findings of this study are available from the corresponding author upon reasonable request.
