# Supplementary material for: Lunar‐Based Photothermal CO2 Reduction Strategy: Self‐Evolving Transient Active Interface and Band Engineering
Source: Adv Sci (Weinh). 2026 May 6;13(41):e75487. doi: 10.1002/advs.75487 (PMC13335664; doi:10.1002/advs.75487)
Supplement: Supplementary file 1 — Supporting File: advs75487‐sup‐0001‐SuppMat.docx. [file ADVS-13-e75487-s001.docx]

**Supporting Information**

**Lunar-based photothermal CO_2_ reduction strategy: self-evolving transient active interface and band engineering**

Yahang Wang,^a^ Yuhuan Li,^b^ Quanxin Wang,^c^ Deng Li,^d^ Pakkin Leong,^a^ Meng Wang,^c^ Shi Feng,^,a^ Runyang Mo,^c^ Xianjin Shi,^e^ Xiaohui Li,^c^ Gangqiang Zhu^c^ and Chipui Tang*^,a,f^

*^a^* *State Key Laboratory of Lunar and Planetary Sciences,* *Macau University of Science and Technology,* *Taipa 999078, Macao, P. R. China*

*^b^ School of Instrument Science and Opto-Electronics Engineering, Beijing Information Science and Technology University, Beijing 100192, China*

*^c^ School of Physics and Information Technology, Shaanxi Normal University, Xi’an 710062,* *P. R. China*

*^d^ School of Materials Science and Engineering, Shaanxi Normal University, Xi’an 710119, Shaanxi, China*

*^e^ State Key Lab of Loess and Quaternary Geology (SKLLQG), Institute of Earth Environment, Chinese Academy of Sciences, Xi’an 710061, P. R. China*

*^f^ Faculty of Innovation Engineering, Macau University of Science and Technology, Taipa 999078, Macao, P. R. China;*

*Corresponding author: Tel/Fax: +853 8897 1923;

Email address: *[cptang@must.edu.mo](mailto:cptang@must.edu.mo)*

**Supplementary Notes**

**Supplementary Note 1. Stability of catalysts at extremely low temperatures**

The temperature on the lunar surface is -153 to 140°C,1 and the temperature drops sharply when the sun is not shining on the lunar surface. In order to explore the stability of the catalyst sample at extremely low temperatures, the sample was cooled to extremely low temperatures. The catalyst Pd/Ov-FeTiO_3_ sample was placed in a magnetic boat and wrapped with tin foil. The tin foil was placed in a plastic measuring cup and liquid nitrogen was added. The sample was immersed in liquid nitrogen and frozen for 30 min as shown in Figure S1.

**Supplementary Note 2. XRD, TEM and other catalyst characterization**

Crystal structures of all kinds of sample catalysts were analyzed with the X-ray diffraction (XRD) technique (Model D8 Advance, Bruker, Germany) with graphite high-intensity Cu Kα (λ = 0.15418 nm) radiation. XRD spectra were taken for 2θ angles from 10°- 80° at a scan rate of 0.02°/min. Accelerating voltage and electric current were 40 kV and 40 mA, respectively.

The Brunauer Emmett Teller (BET) method was used for the specific surface area of the samples, and the Barrett Joyner Halenda (BJH) method was used for the pore diameter. N_2_ adsorption and desorption, BET and pore size were tested using BSD-660M from BSD Instrument Technology (Beijing) Co., Ltd. Atomic force microscopy (KPFM) tests were performed using AtomExplorer from Truth Instruments Co., Ltd. The probe model was: HQ: NSC18/Pt, and the measurement range was 5 μm×5 μm. The X-ray photoelectron spectroscopy (XPS) of the sample was performed by Escalab MkII spectrometer (VG Scienta, Node Materials: Al Kα, HV=1486.6 eV).

**Supplementary Note 3. In-situ FTIR** **test**

The sample was placed in a small crucible and then placed in an in-situ infrared testing chamber. The temperature was raised to 120°C under a nitrogen atmosphere and heat-treated for 30 minutes. After cooling to room temperature, measurements were taken under dark conditions at room temperature, light conditions at room temperature, and light conditions at 300°C. The gas mixture was 5% CO_2_ + H_2_, with the remaining 5% balanced with N_2_.

**Supplementary Note 4. Temperature Programmed Desorption (TPD) tests**

The TPD test of the catalyst was conducted on the catalyst dynamic characterization system. The specific steps of the test are as follows:

(1) Preparation stage: Place quartz wool at the bottom of the thicker section of the U-shaped tube, place 50 mg of catalyst on the quartz wool, and install the quartz tube into the catalyst dynamic characterization system;

(2) Pretreatment stage: Under argon purge, set the pretreatment temperature to 150℃, the heating rate to 10 ℃/min, and the pretreatment time to 2 h. After the treatment, wait for the temperature to drop to 50℃, purge with test gas (H_2_) for 60 min, and wait for baseline equilibrium. After the purge, cool down to room temperature to prepare for the next stage of the test;

(3) Test stage: The purge gas remains unchanged or is changed to argon, set the test temperature to 700℃, the heating rate to 10 ℃/min, and the test ends when the temperature rises to 700℃. During the test, the gas passing through the catalyst is monitored by a thermal conductivity detector (TCD).

**Supplementary Note 5. X-ray Absorption Fine Structure (XAFS) test**

X-ray absorption fine structure (XAFS) spectroscopy data of the Ti element were collected from the BL07U beamline of the Shanghai Synchrotron Radiation Facility, using the TEY mode. XAFS of the Fe sample was collected from the following equipment. The test specimens were prepared by the KBr-disk method. XAFS spectroscopy was carried out using the Rapid-XAFS1M (Anhui Ab-sorption Spectroscopy Analysis Instrument Co., Ltd,) by transmission mode at 25 kV and 40 mA.

**Supplementary Note 6. Evaluation of Catalyst CO_2_ Hydrogenation Performance**

Take 50 mg of catalyst sample (0.05 g for natural catalysts such as ilmenite) and place it in a quartz tube. Then place the quartz tube in a photothermal reaction device. The catalyst undergoes CO_2_ hydrogenation reaction under the conditions of 1 MPa pressure, 1:1 H_2_/CO_2_ ratio, and 12000 mL·g^-1^·h^-1^ space velocity. The temperature rise interval is 50°C, and 3 points are taken at each temperature point from room temperature to 300°C. At the same time, photothermal and thermal conditions are tested intermittently. For the catalyst selection process, the test is directly carried out under the conditions of 300°C plus light.

The selectivity of CO is calculated using formula (1), where the total yield is the sum of all products.

$CO selectivity=\frac{CO yield}{Total yield}$ (1)

**Supplementary Note 7. Catalyst performance evaluation at different wavelengths**

The performance of the catalyst was tested at 300°C by adding bandpass filters of 380 nm, 400 nm, 475 nm, 550 nm, 650 nm and 700 nm to the xenon lamp. The 800 nm test used a cutoff filter to filter out light below 800 nm.

**Supplementary Note 8. Transient absorption kinetics fitting**

Global fitting was performed on the transient absorption data. Since both sets of data had significant background signals, background subtraction was performed. A sequential model with three parameters was used for global fitting, yielding accurate fitting results. Specific kinetic data are shown in the table above. The table also includes time-cut spectra extracted from the original data, the fitted (SAS) spectra, the evolution curve of species concentration over time, and the kinetics at selected wavelengths (600/750 nm).

The rate constant is then calculated using formula (2).

$k=\frac{1}{\tau}$ (2)

The rate constant (k_ET_) of electron transfer from the FeTiO_3_ main body to the Pd metal interface caused by the introduction of Pd and Ov catalysts is calculated using formula (3).

$k_{ET}=k_{2,({Pd-FeTiO}_{3})}-k_{2,({FeTiO}_{3})}$ (3)

The charge transfer quantum efficiency (𝜂_𝐸𝑇_) can be further inferred.

$\eta_{ET}=\frac{k_{ET}}{k_{2({Pd-FeTiO}_{3})}}$ (4)

**Supplementary Note 9. DFT calculation details**

We used the first-principles calculation method based on density functional theory (DFT) and used the Vienna ab initio simulation package (VASP) for calculations. The interaction between ions and electrons was described by the augmented wave (PAW) method, and the exchange correlation was described by the generalized gradient approximation (GGA) of the Perdew-Burke Enzerhor (PBE) functional. Both the FeTiO_3_ surface and the Pd/Ov-FeTiO_3_ surface were calculated using a 3×3×1 supercell. The cutoff energy was set to 400 eV. A 20 Å vacuum layer was set to avoid periodic repetition effects. The Brillouin zone was sampled with a 1×1×1 k-point grid, centered on the geometry optimization. The energy convergence criterion and mechanical convergence criterion for all structural optimizations were set to 1.0 × 10^-4^ eV/atom and 0.01 eV/Å, respectively.

The adsorption energy (E_ads_) is calculated as follows:

E_ads_ = E_total_ – E_surface_ – E_adsorbates_

Where E_total_, E_surface_ and E_adsorbates_ are the energy of substrate and adsorbate, respectively.

The change in the Gibbs free energy (∆G) value of each step in the reduction process is calculated by correcting the zero-point energy and entropy of DFT. The calculation formula is as follows:

∆G=∆E+∆ZPE-T∆S

∆E is obtained by DFT calculation, ∆ZPE is the zero-point energy, T is 298.15 K, and ∆S is the entropy change.

**Supplementary Note 10. Outdoor CO_2_ Hydrogenation Reduction Experiment**

This experiment was conducted on July 27, 2025, in Xi'an, Shaanxi Province, China (latitude 34.258°, longitude 108.929°, and altitude 416 meters). The experimental gas consisted of 5% CO_2_, 5% H_2_, and the balance N_2_, at a pressure of 0.1 MPa.

**Supplementary Note 11. Materials and Methods**

**Materials**

Ferrous chloride (99.5%), titanium tetrachloride (>99%), citric acid monohydrate (>99%), potassium tetrachloropalladate (>98%), titanium oxide (>99%), ferric oxide (>96.5%), manganese oxide (>99.5%) and ethanol (A.R. grade) were all provided by Shanghai Aladdin Biochemical Technology Co., Ltd. Ilmenite is produced in Wenshan, Yunnan, China. Titanium oxide (>99.9%), chromium oxide (>99%) and zirconium silicate (>99%) were all provided by Sinopharm Chemical Reagent Co., Ltd. The simulated lunar soil model was provided by the Chinese Academy of Sciences. Simulated lunar soil 1 is CAS-1, a basalt-type simulated lunar soil that mainly simulates the lunar sea area (mainly composed of plagioclase, pyroxene and ilmenite). Simulated lunar soil No. 2 is a highland-type simulated lunar soil, which mainly contains amorphous or glass and a small amount of olivine.

**Synthesis of FeTiO_3_ and Pd/Ov-FeTiO_3_**

FeTiO_3_ was prepared by sol-gel method. In 0℃ water, 2 mmol of TiCl_4_ and 2 mmol of ferrous chloride were added to 100 mL of deionized water and stirred for 30 minutes. Then the temperature was raised to 80℃ and stirring was continued for 1 h to form a sol. The prepared sol was placed in a vacuum drying oven for drying to obtain a yellow-brown gel. The ground sol was placed in a magnetic boat and calcined at 300℃ and 500℃ for 2 hours each, with a heating rate of 3 ℃/min. Then it was washed alternately with deionized water and anhydrous ethanol three times, and finally vacuum dried at 70℃. The final product was a red-brown FeTiO_3_ sample.

0.5g of FeTiO_3_ catalyst was dispersed in 50mL of deionized water and stirred for 0.5 hours. Then a Pd solution with a VOL of 0.5% was added and stirred for 1h. Stirring was continued for one hour under xenon lamp irradiation, the obtained sample was collected by centrifugation, and then washed alternately with deionized water and anhydrous ethanol three times, and finally vacuum dried at 70℃ to obtain the sample Pd/Ov-FeTiO_3_.

0.00763 g of Pd/Ov-FeTiO_3_ sample was digested with aqua regia and diluted to a final volume of 50 mL. The sample was compared with a standard solution using inductively coupled plasma optical emission spectrometry (ICP-OES, Thermo ICAP PRO, detection limit 0.01 mg/L). The final ICP test revealed that the Pd content in Pd/Ov-FeTiO_3_ was 0.11 wt%.

**Supplementary Note 12. In-situ XPS Test**

This experiment used an X-ray photoelectron spectrometer (ThermoFischer, ESCALAB 250Xi, USA) for XPS testing. The spectrometer consists of three parts: a preparation chamber, a reaction cell, and an analysis chamber. The sample can be transferred between the different chambers using a metal rocker arm. The analysis chamber was set to a vacuum of 8**×**10^-10^ Pa, with Al_2_K_3_ rays (hv = 1486.8 eV) as the excitation source, an operating voltage of 12.5 kV, and a filament current of 16 mA. Ten cycles of signal accumulation were performed. The passing energy was 40 eV with a step size of 0.1 eV, and charge correction was performed using the C₁s = 284.80 eV binding energy as the energy standard. The specific testing steps are as follows:

Step 1: First, evacuate the analysis chamber to (2**×**10⁻⁶ mBar). Open the valves between the analysis chamber and the preparation cell, push the sample in, and then close the valves.

Step 2: Test the XPS of the sample under vacuum at room temperature;

Step 3: Irradiate the sample with a light source (power: 1000 mW) and test the XPS under illumination conditions;

Step 4: Subsequently, heat the sample to 300±5°C and irradiate it with the light source to test the XPS under photothermal conditions.

Test complete.


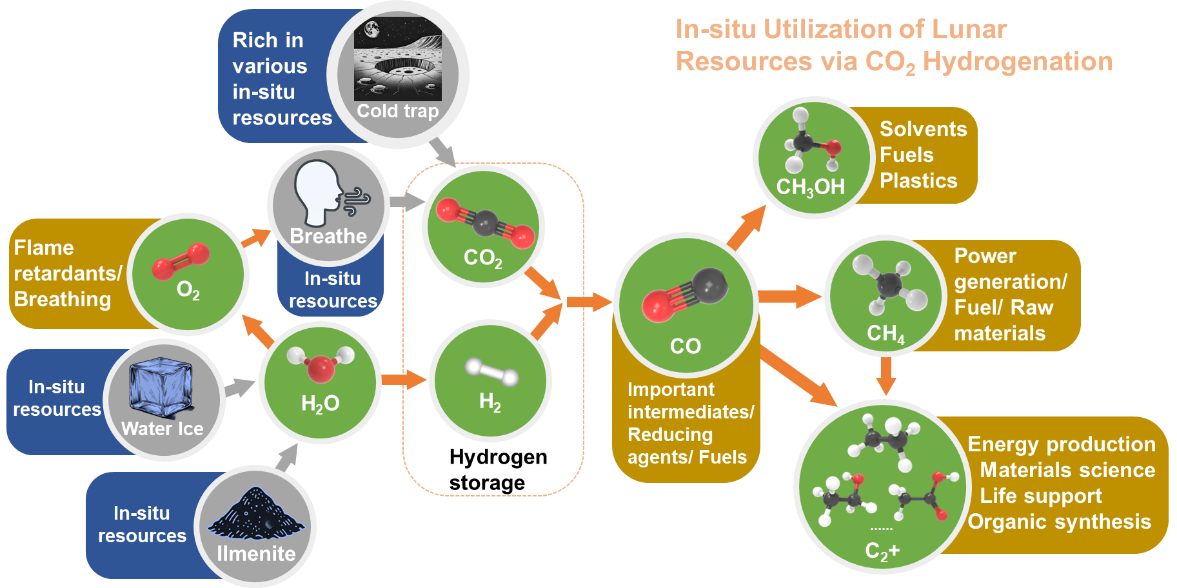


**Supplementary Figure 1.** Diagram of the mechanism of in-situ carbon-hydrogen recycling on the lunar surface.


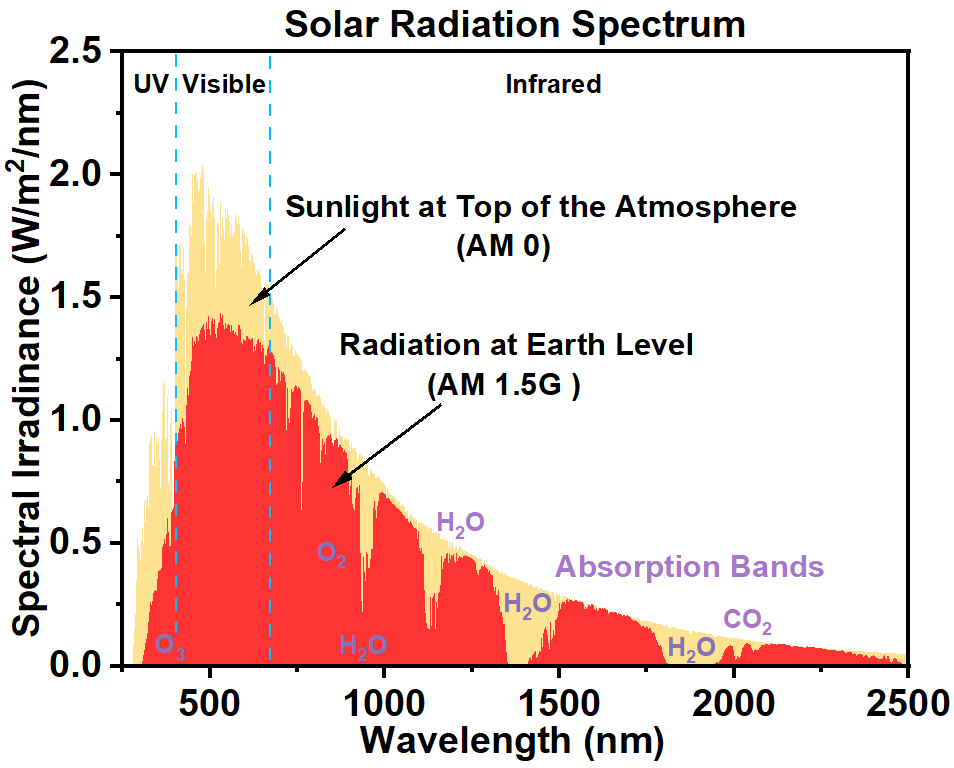


**Supplementary Figure 2.** Solar spectrum on the lunar surface.^2^

^2^ ASTM G173-03(2012) Standard tables for reference solar spectral irradiances: direct normal and hemispherical on 33° tilted surface.

Atmospheric mass (AM) is a key physical quantity that measures the degree to which the atmosphere attenuates solar radiation. Outside the atmosphere in Earth's orbit, solar irradiance is constant at 1353 W/m^2^ (AM 0, the solar constant); while when the solar zenith angle is 48.2°, the path of light through the atmosphere is defined as AM 1.5, corresponding to a ground standard irradiance of 1000 W/m² (AM 1.5G). Unlike Earth, the Moon lacks an atmosphere composed of H_2_O, O_2_, and O_3_, thus eliminating the absorption and scattering interference of specific wavelengths of radiation.^2^ This vacuum environment makes the solar spectrum on the Moon's surface more direct and purer than that on Earth, with an energy abundance comparable to or even superior to Earth's standard spectrum. Combining the Moon's "Eternal Peak" of sunlight, which provides over 90% of the daylight hours and a constant light temperature of approximately 130°C, with the aid of a simple Fresnel lens, the temperature can be easily raised to over 300°C.^3,4^ This excellent light and heat condition makes the Moon an ideal natural laboratory for conducting photothermal catalysis research.


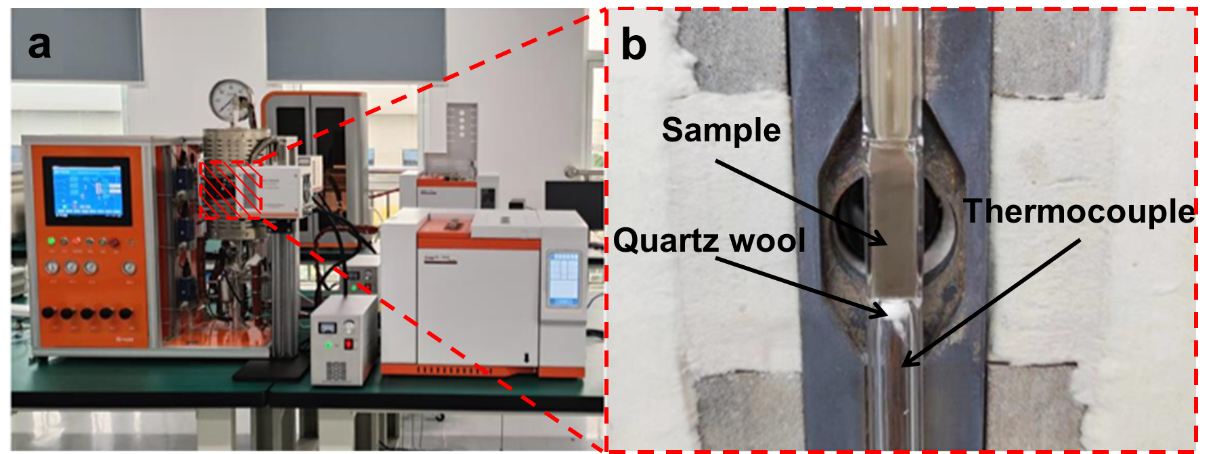


**Supplementary Figure 3.** (a) Catalyst photothermal CO_2_ hydrogenation performance evaluation system, (b) Sample placement in the evaluation system.


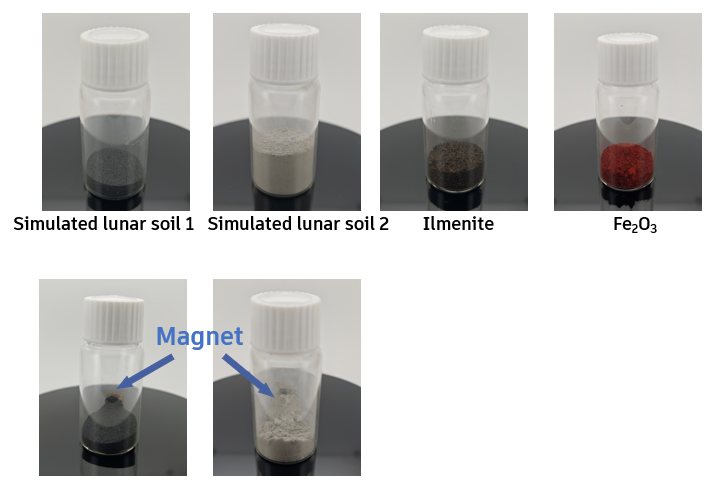


**Supplementary Figure 4.** Photos of simulated lunar soil, ilmenite and iron oxide.

The simulated lunar soil can be attracted by strong magnets, proving that it contains a significant amount of Fe. At the same time, according to the literature, ilmenite has paramagnetism and can be easily enriched by magnetic separation, electrostatic separation and other means.^5^


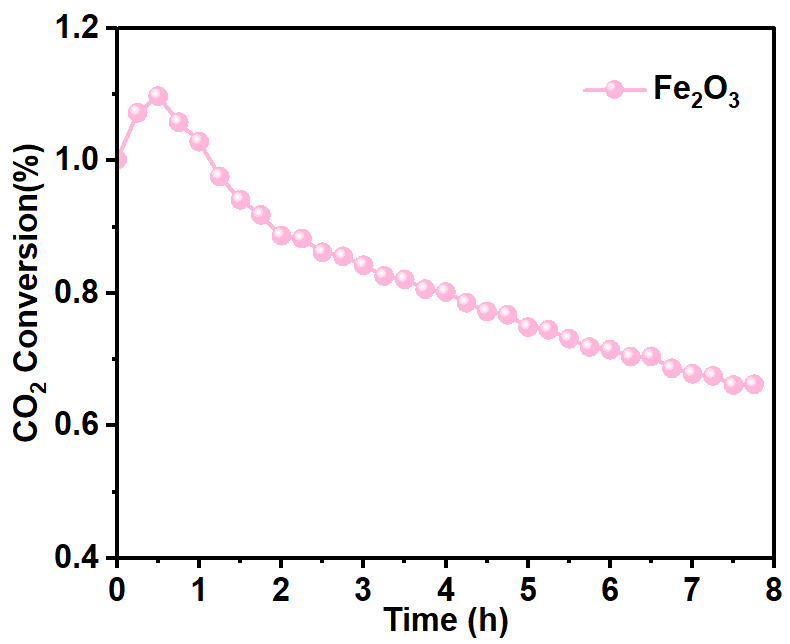


**Supplementary Figure 5.** Iron Oxide Stability Test.

In the process of testing CO_2_ hydrogenation, it was found that Fe_2_O_3_ also has good activity. However, it was found that its activity decreased significantly under longer testing conditions. Therefore, it is not used as an alternative catalyst.


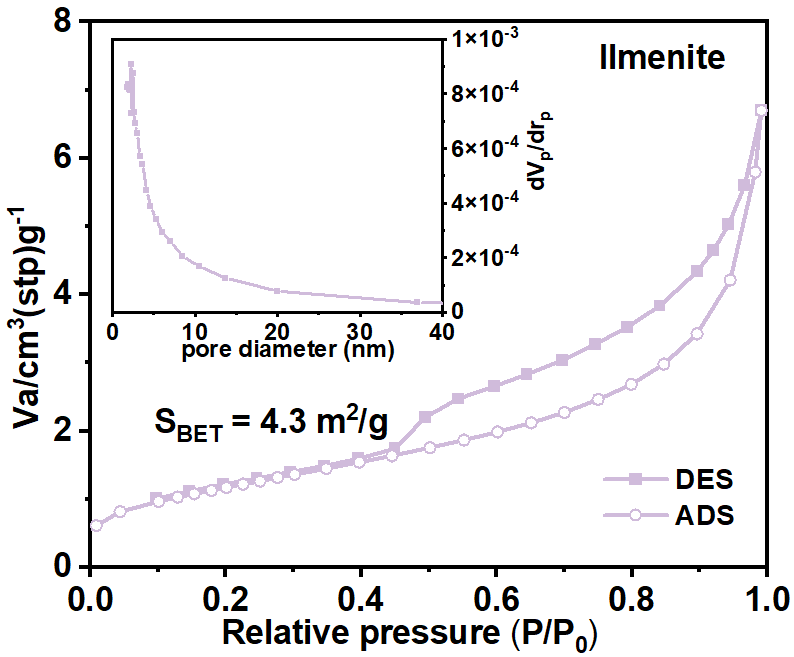


**Supplementary Figure 6.** N_2_ isothermal adsorption and desorption and pore size distribution of ilmenite.

The ilmenite obtained from Yunnan, China cannot fully represent the ilmenite found in lunar soil due to its relatively small specific surface area. Therefore, in this study, FeTiO₃ was synthesized via the sol-gel method to better simulate lunar conditions.


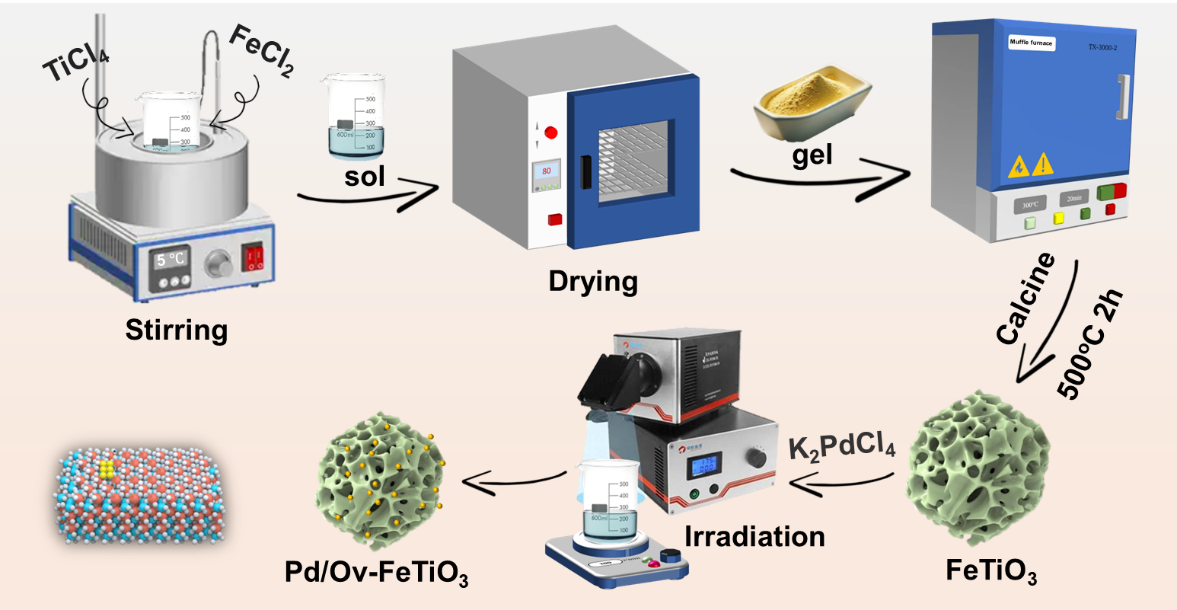


**Supplementary Figure 7.** Synthesis flow chart of FeTiO_3_ and Pd/Ov-FeTiO_3_.

FeTiO_3_ was synthesized via a sol-gel method, and Pd nanoparticles were then loaded onto the substrate via photodeposition.


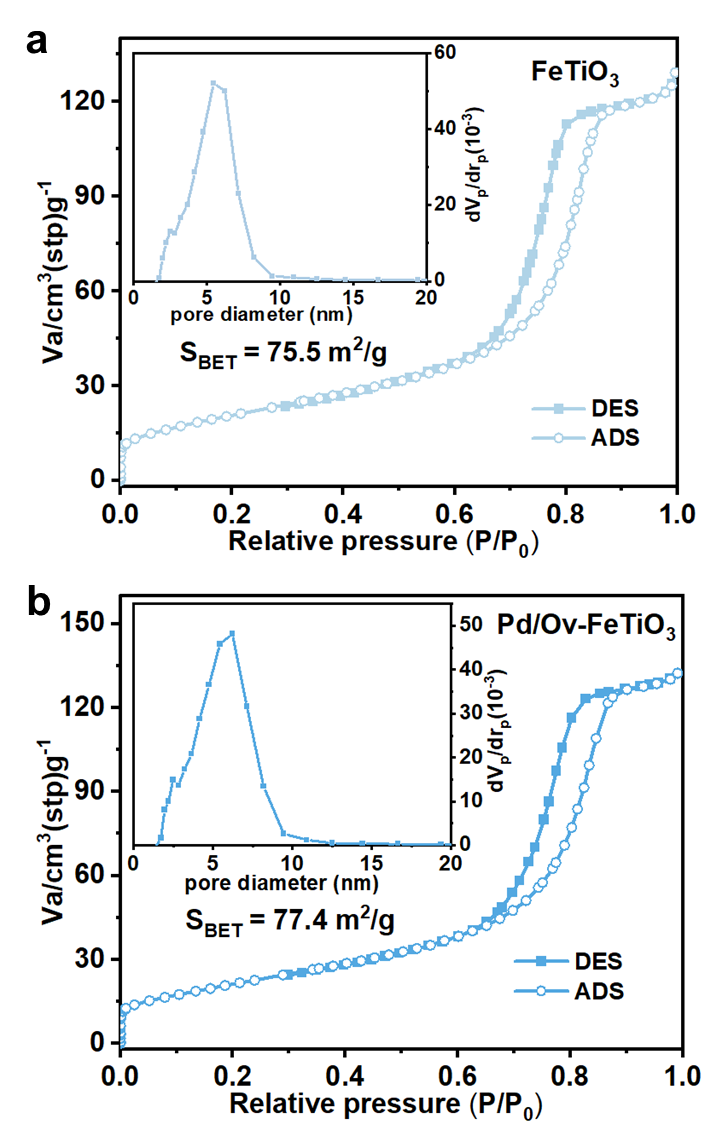


**Supplementary Figure 8.** N_2_ isothermal adsorption-desorption and pore size distribution of FeTiO_3_ (a) and Pd/Ov-FeTiO_3_ (b).


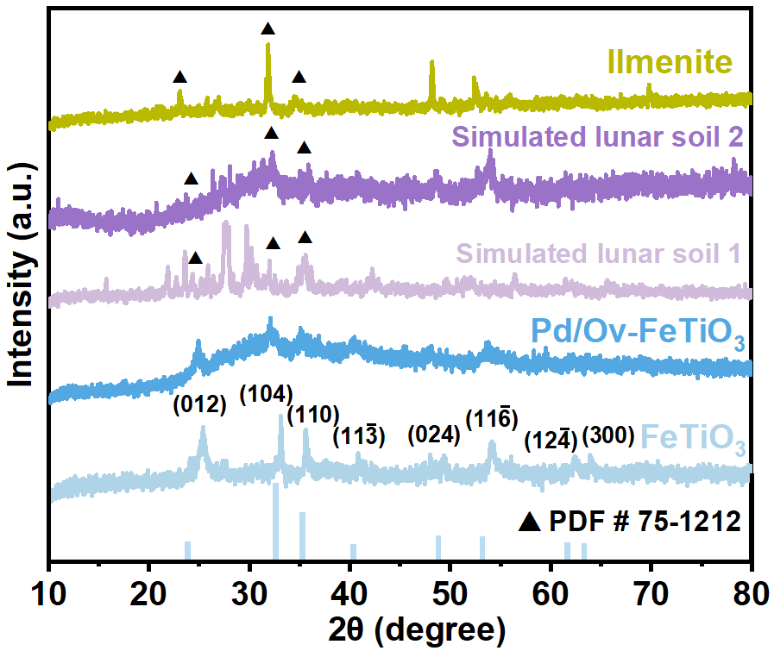


**Supplementary Figure 9.** XRD patterns of ilmenite, simulated lunar soil, FeTiO_3_ and Pd/Ov-FeTiO_3_.


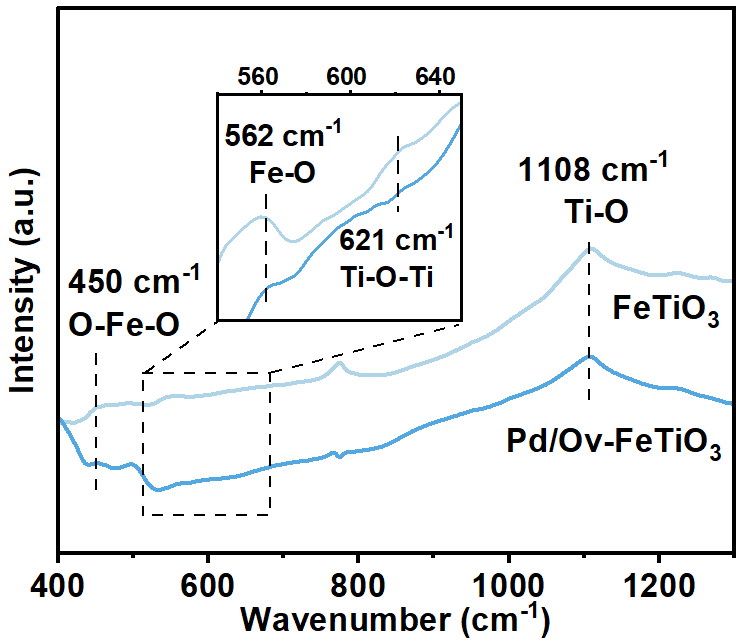


**Supplementary Figure 10.** FTIR spectra of FeTiO_3_ and Pd/Ov-FeTiO_3_.

The results showed that the key functional groups, such as Ti–O and Fe–O^6,7^ remained unchanged after Pd introduction, confirming that the modification preserved the original structure.


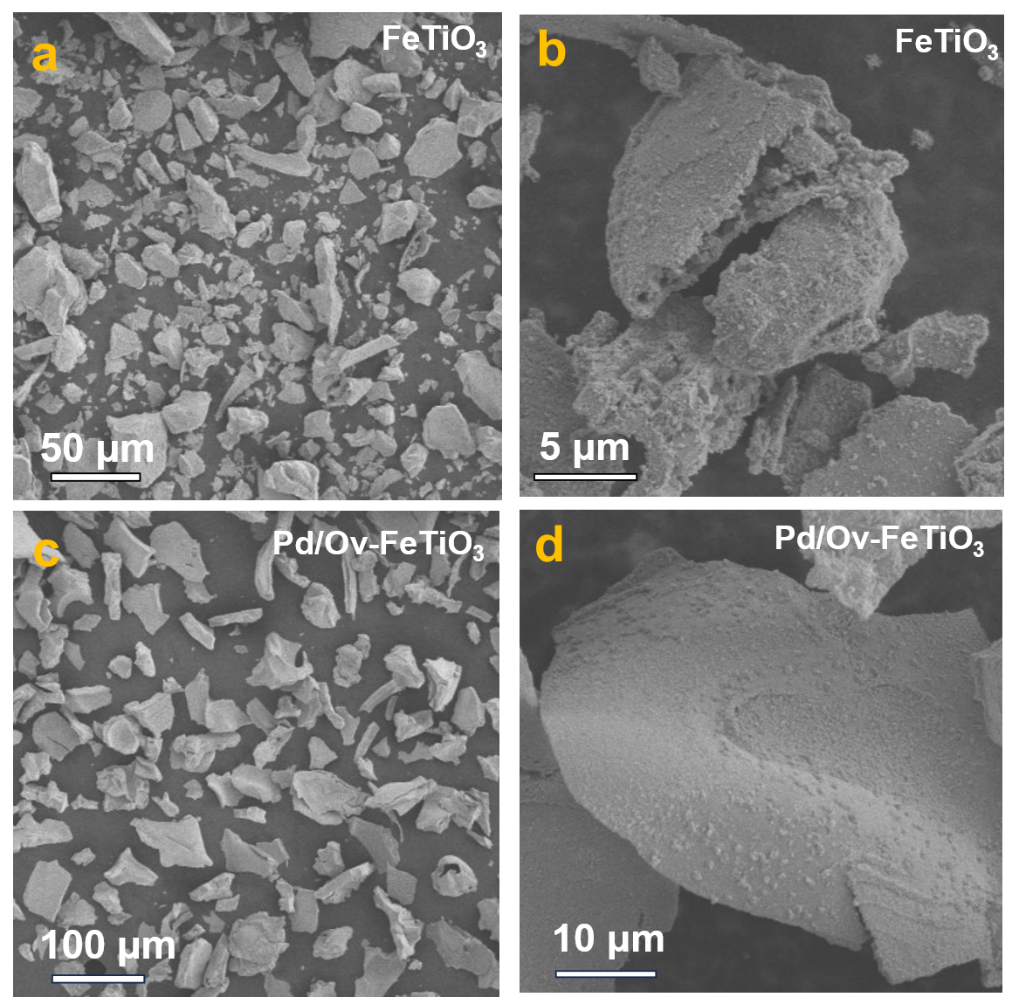


**Supplementary Figure 11.** SEM image of FeTiO_3_ (a, b) and Pd/Ov-FeTiO_3_ (c, d).

As shown in Figure S10, it is the SEM image of FTO before and after modification. The morphology of FTO is a nanoblock of 10 ~ 20 μm. As shown in Figure S9, it is the EDX element distribution image of the sample. It can be observed that the distribution of Pd element on the surface of FTO is relatively uniform. Through high-resolution transmission electron microscopy, it was observed that the FTO block is actually composed of small nanoparticles. According to the literature, there are nano-micro pores in lunar soil ilmenite.^8^ This nanopore can adsorb and store a large number of hydrogen atoms from the solar wind. Each ilmenite molecule (FeTiO_3_) can adsorb 4 hydrogen atoms and is the "water reservoir" of the moon.^8^ As shown in Figure S9, FTO is analyzed by specific surface area and pore size. The prepared FTO has a large specific surface area and its pore size is about 5nm. Interestingly, the FTO synthesized in this work is similar to the ilmenite structure in the lunar soil.


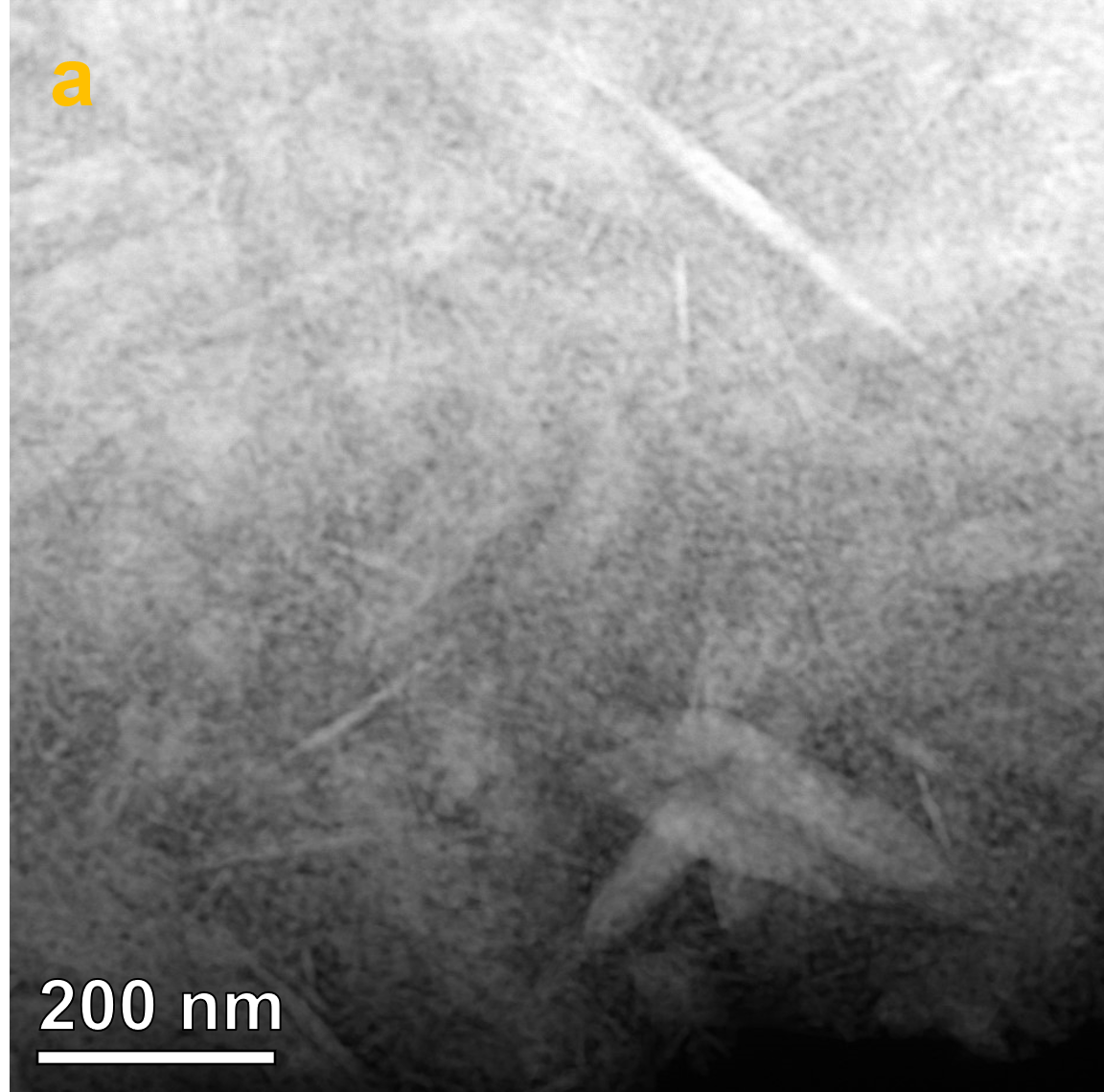

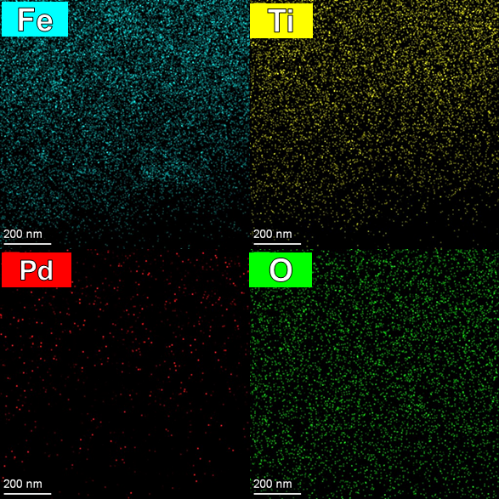


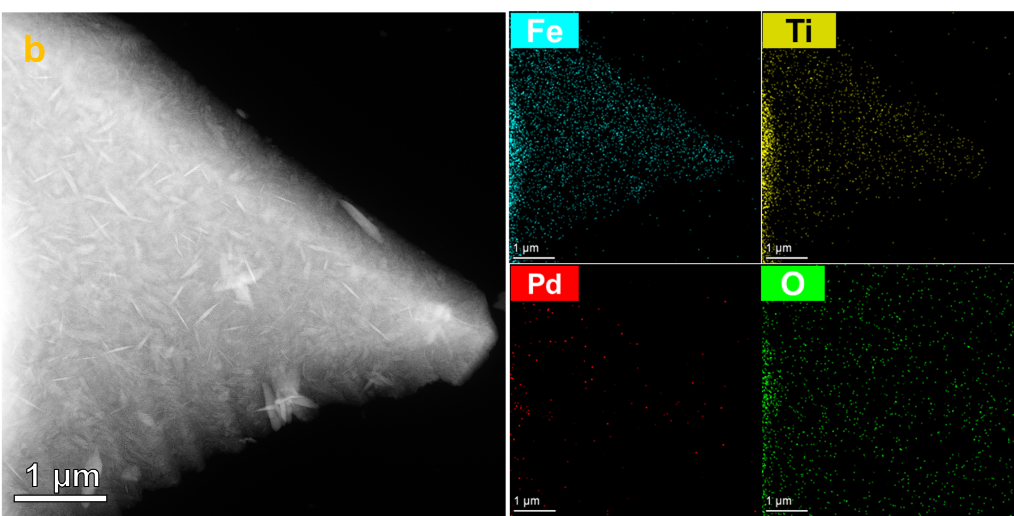


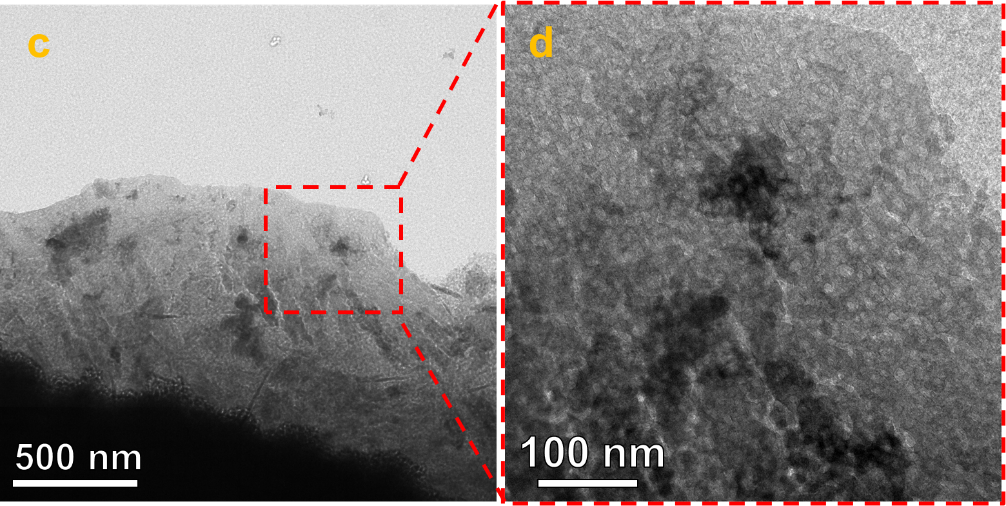


**Supplementary Figure** **12.** (a, b) EDX element-mapping images and (c, d) High-resolution TEM images of Pd/Ov-FeTiO_3._


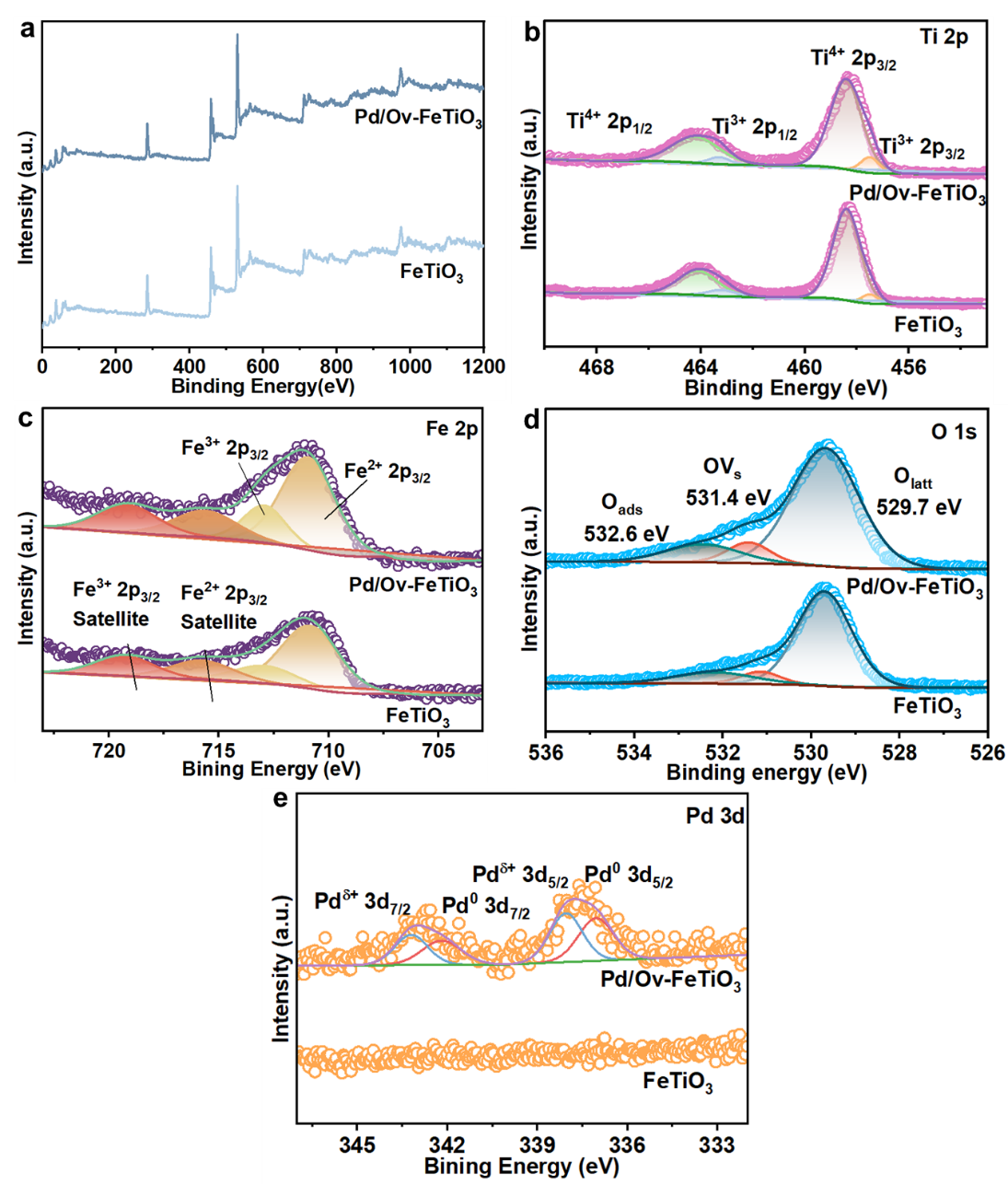


**Supplementary Figure 13.** (a)XPS full spectrum and high-resolution Ti 2p (b), Fe 2p (c), Pd 3d (d), and O 1s (e).


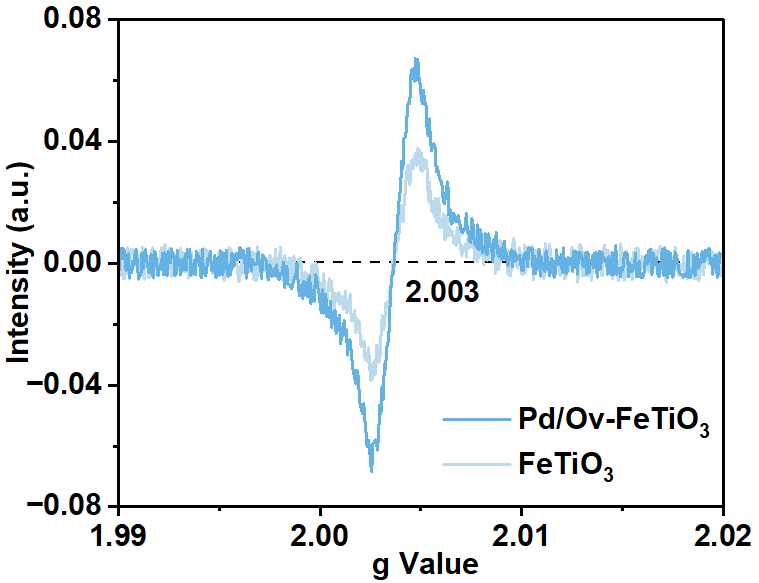


**Supplementary Figure 14.** Electron paramagnetic resonance (EPR) spectra of FeTiO_3_ and Pd/Ov-FeTiO_3_.


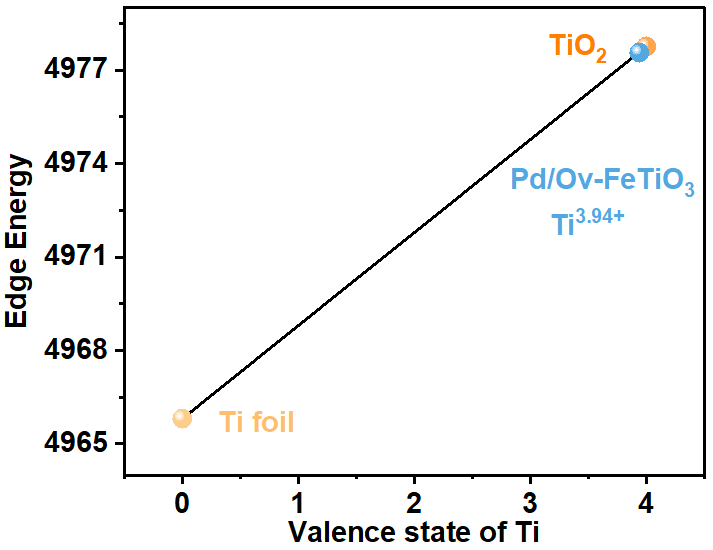


**Supplementary Figure 15.** The oxidation states of the upper Ti in different samples were calculated via Ti K-edge XAFS.


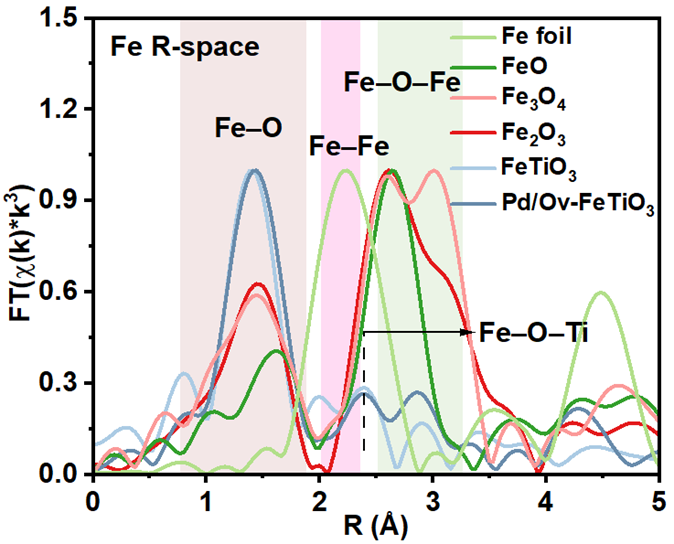


**Supplementary Figure 16.** EXAFS fitting curve of the sample in R space.

**
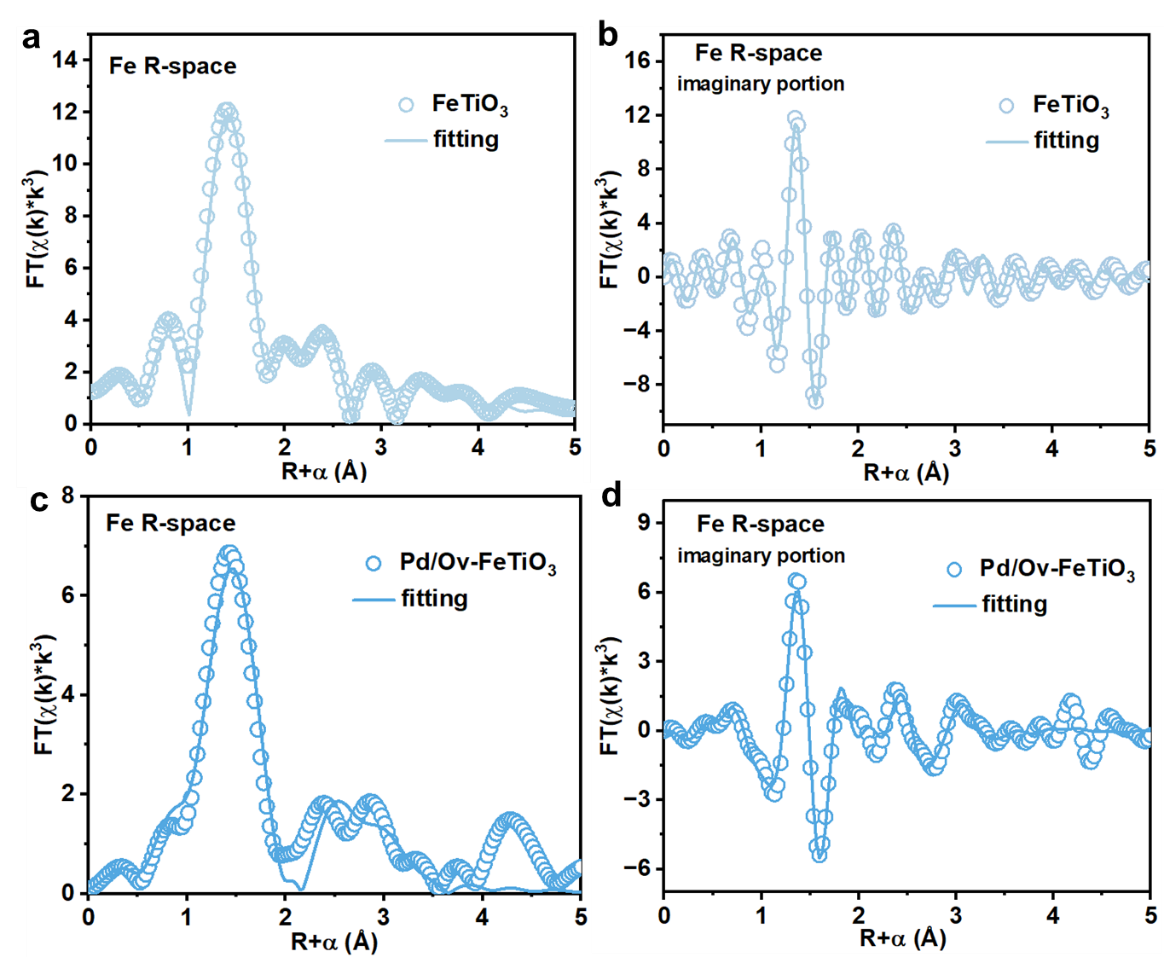
**

**
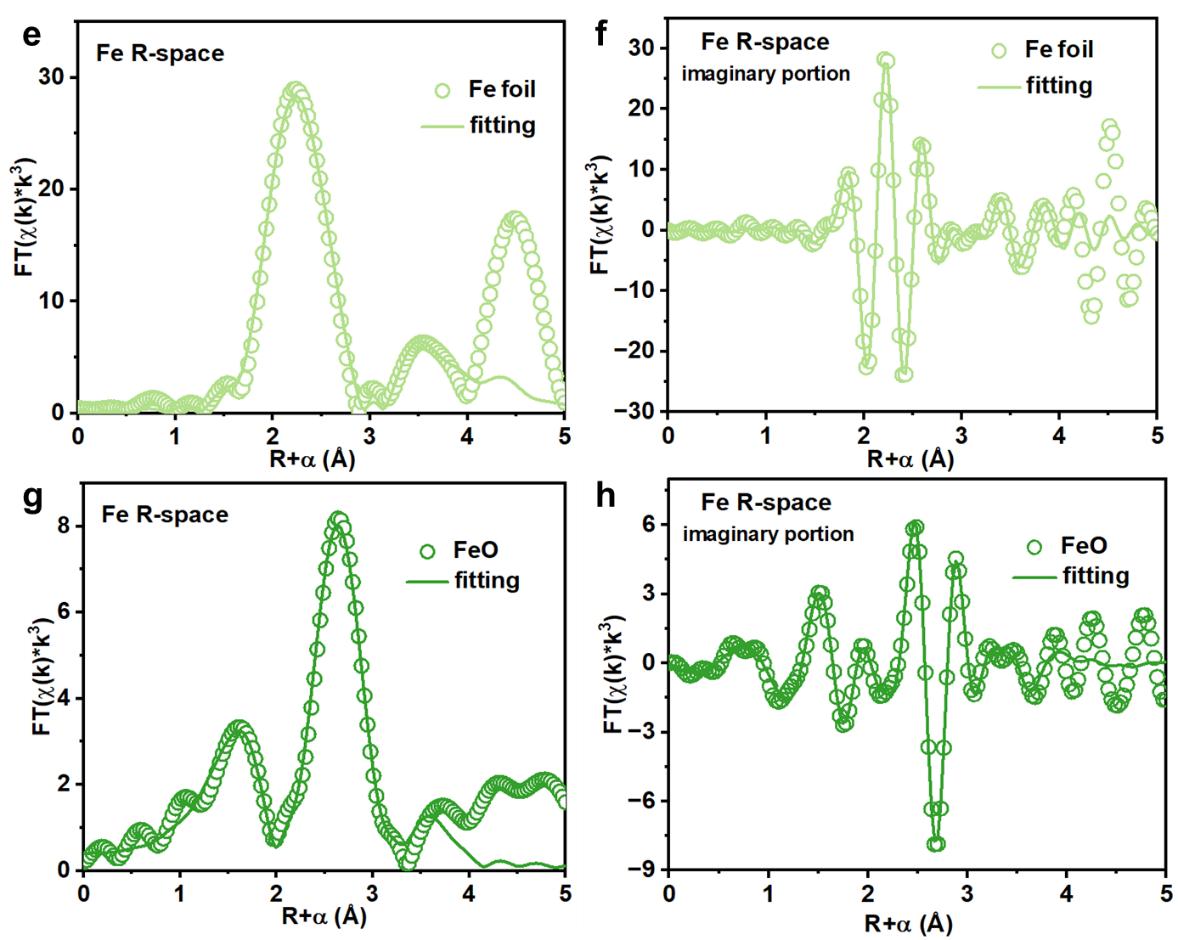
**

**
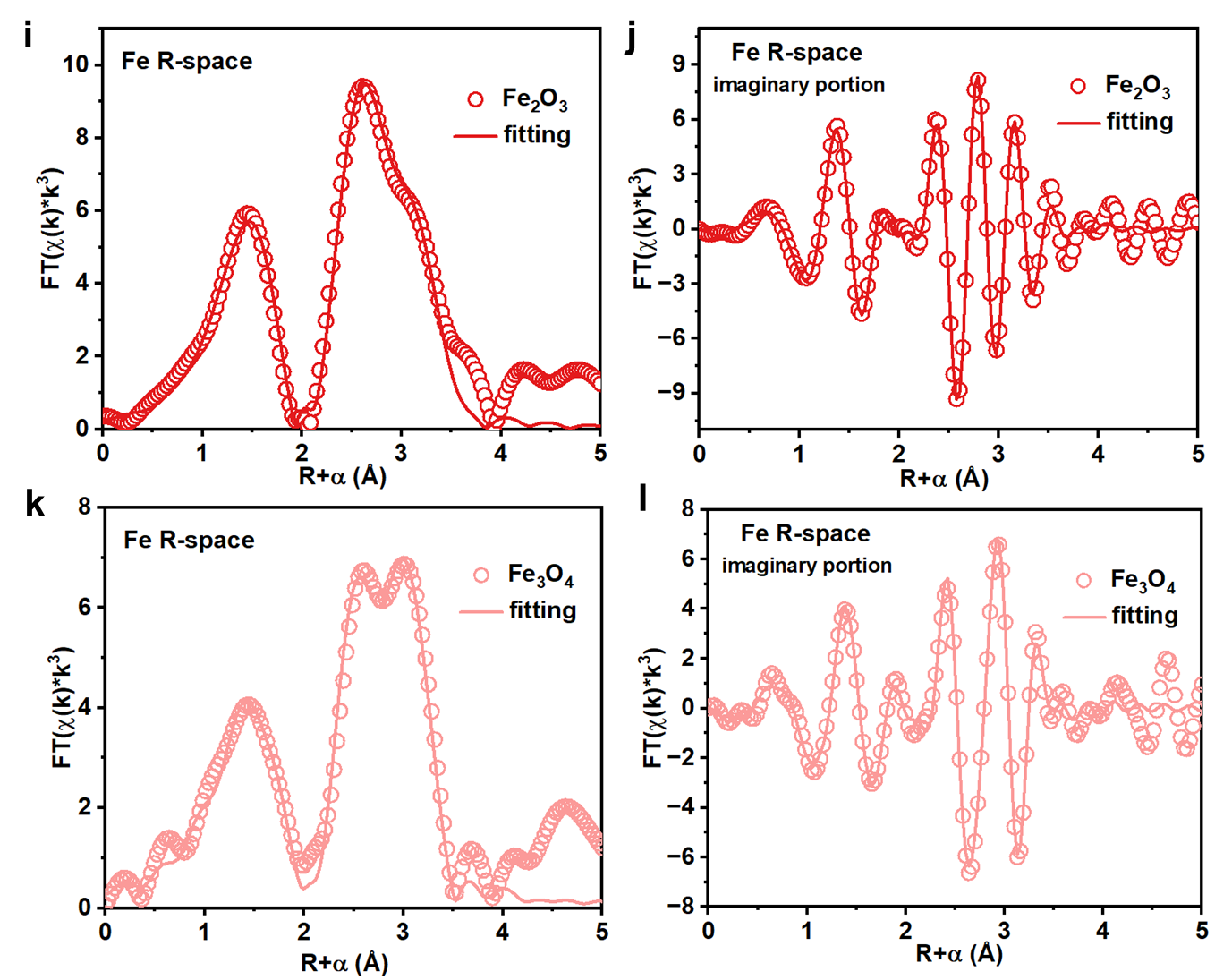
**

**Supplementary Figure 17.** Original data and fitted data of the real and imaginary parts of R space of FeTiO_3_ (a, b), Pd/Ov-FeTiO_3_ (c, d), Fe foil (e, f), FeO (g, h), Fe_2_O_3_ (i, j) and Fe_3_O_4_ (k, i).


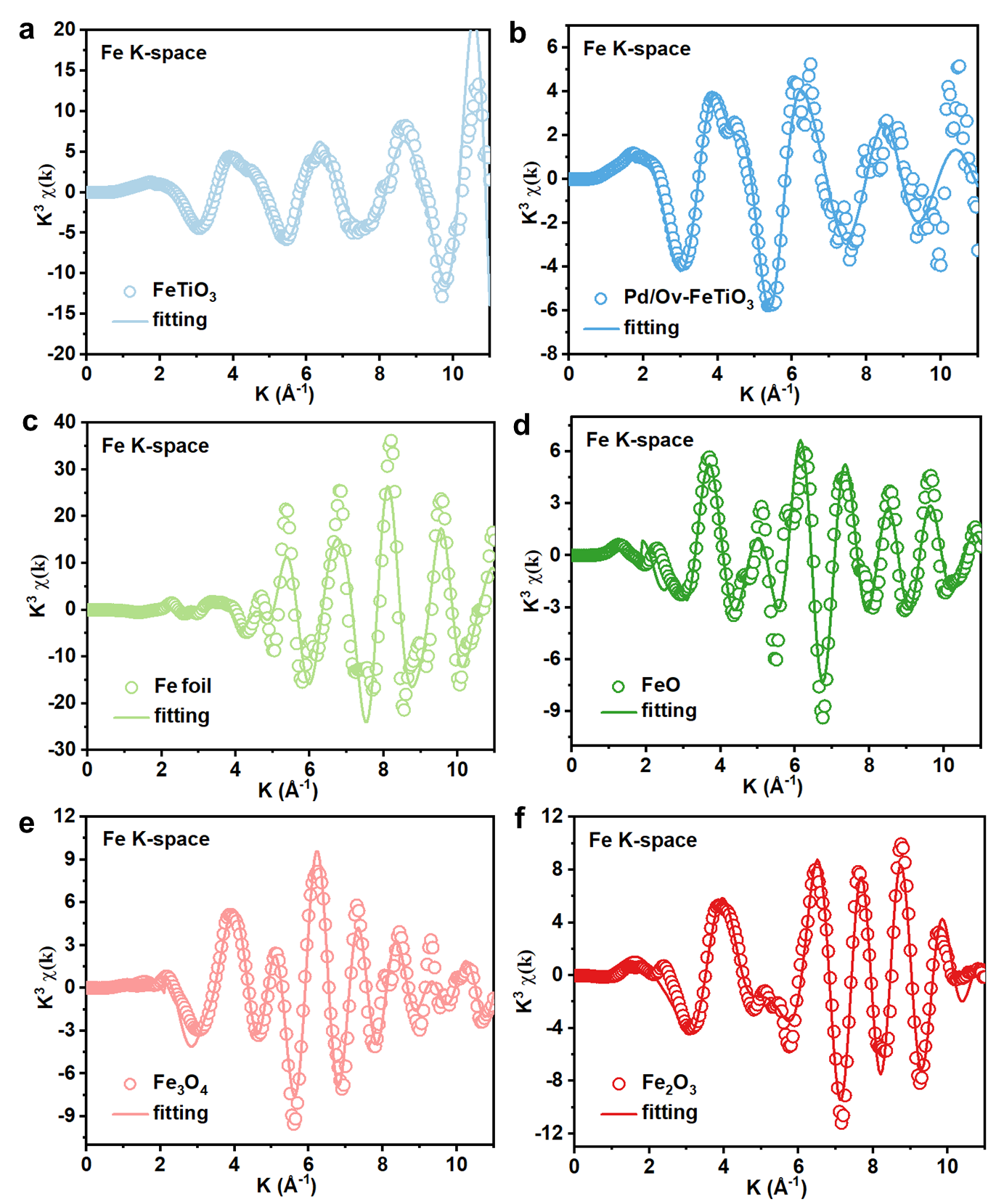


**Supplementary Figure 18.** Original data and fitting data of K space of FeTiO_3_ (a), Pd/Ov-FeTiO_3_ (b), Fe foil (c), FeO (d), Fe_2_O_3_ (e) and Fe_3_O_4_ (f).


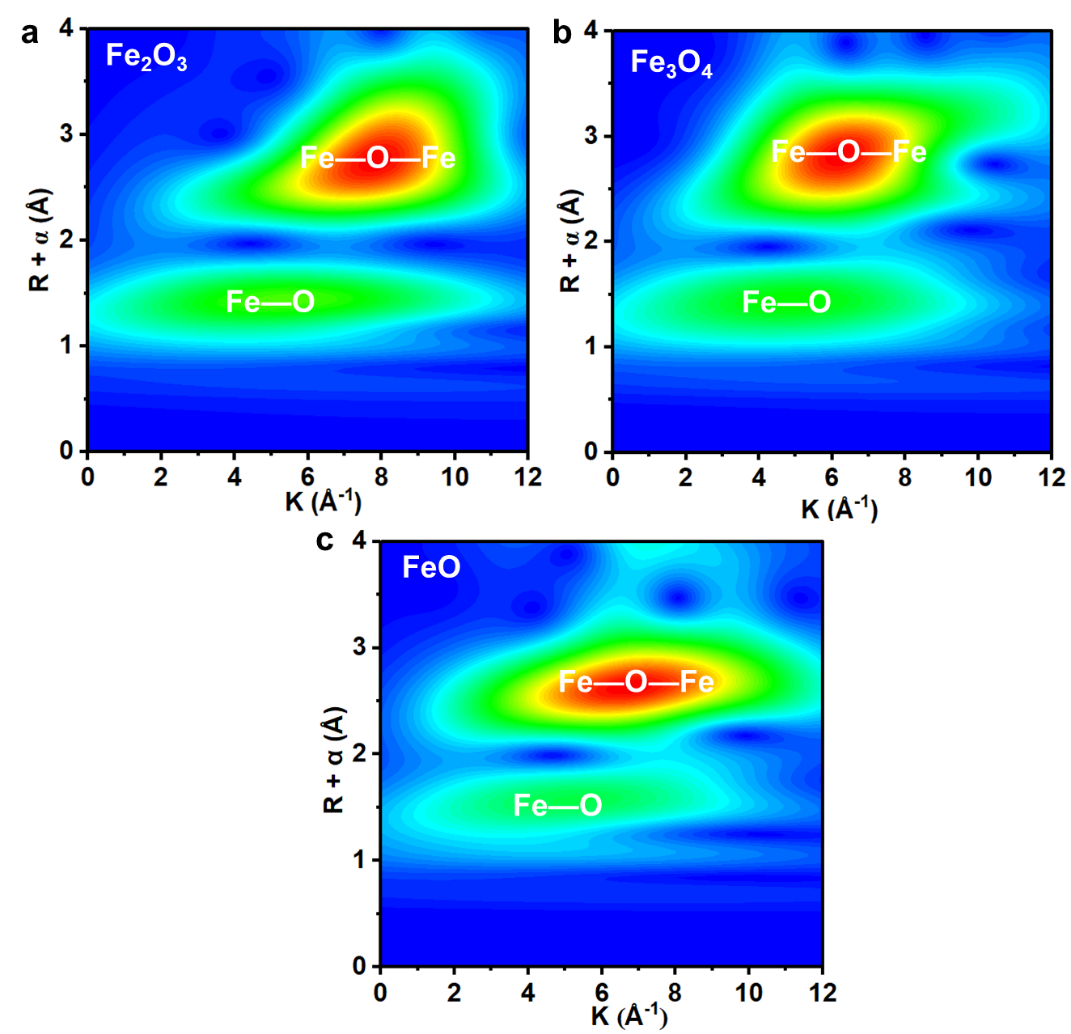


**Supplementary Figure 19.** WT of Fe_2_O_3_(a) Fe_3_O_4_(b) and FeO(c).

**
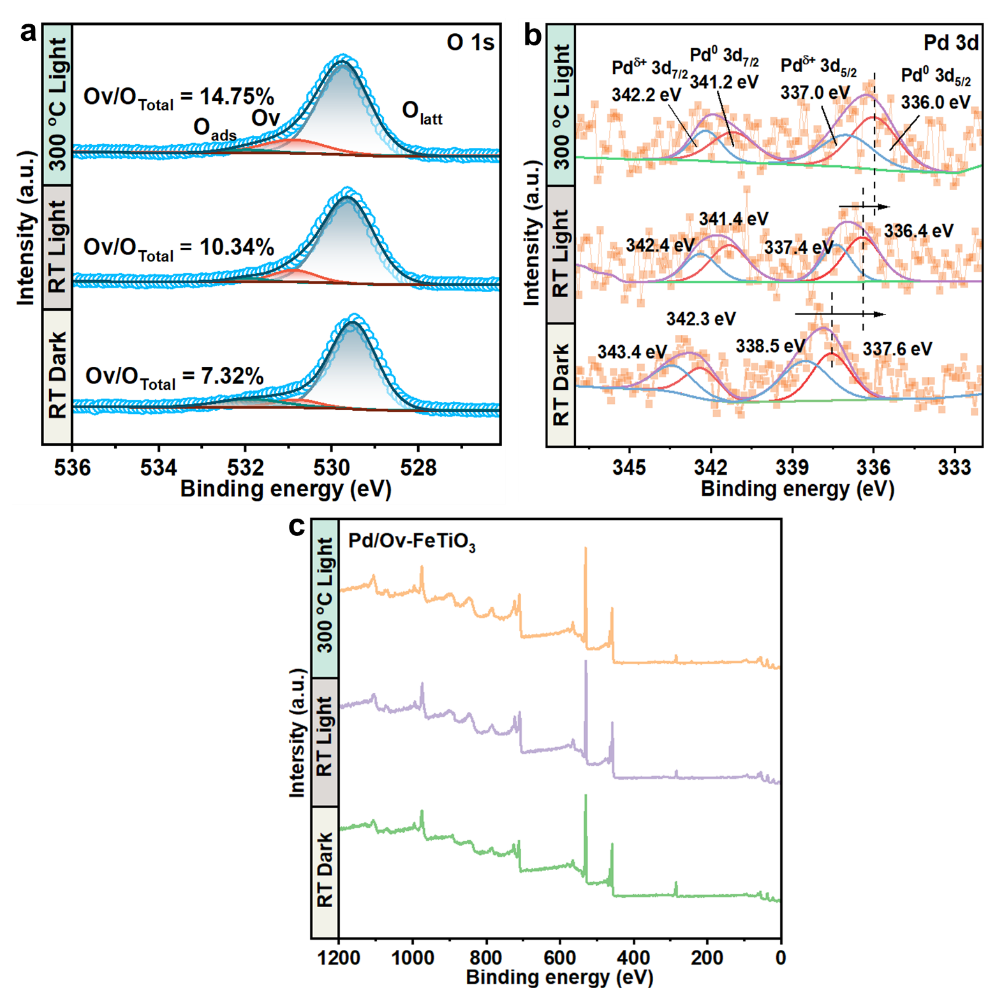
**

**Supplementary Figure 20.** High-resolution in-situ XPS O 1s(a) and Pd 3d (b) and full spectrum (c) of Pd/Ov-FeTiO_3_.


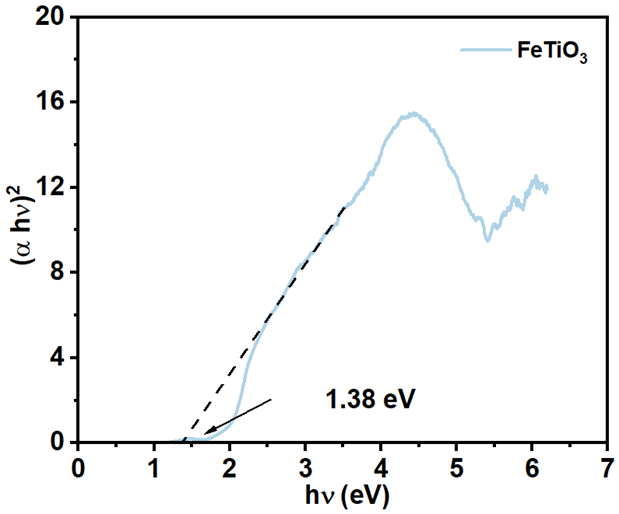


**Supplementary Figure 21.** Tauc plot of FeTiO_3_.


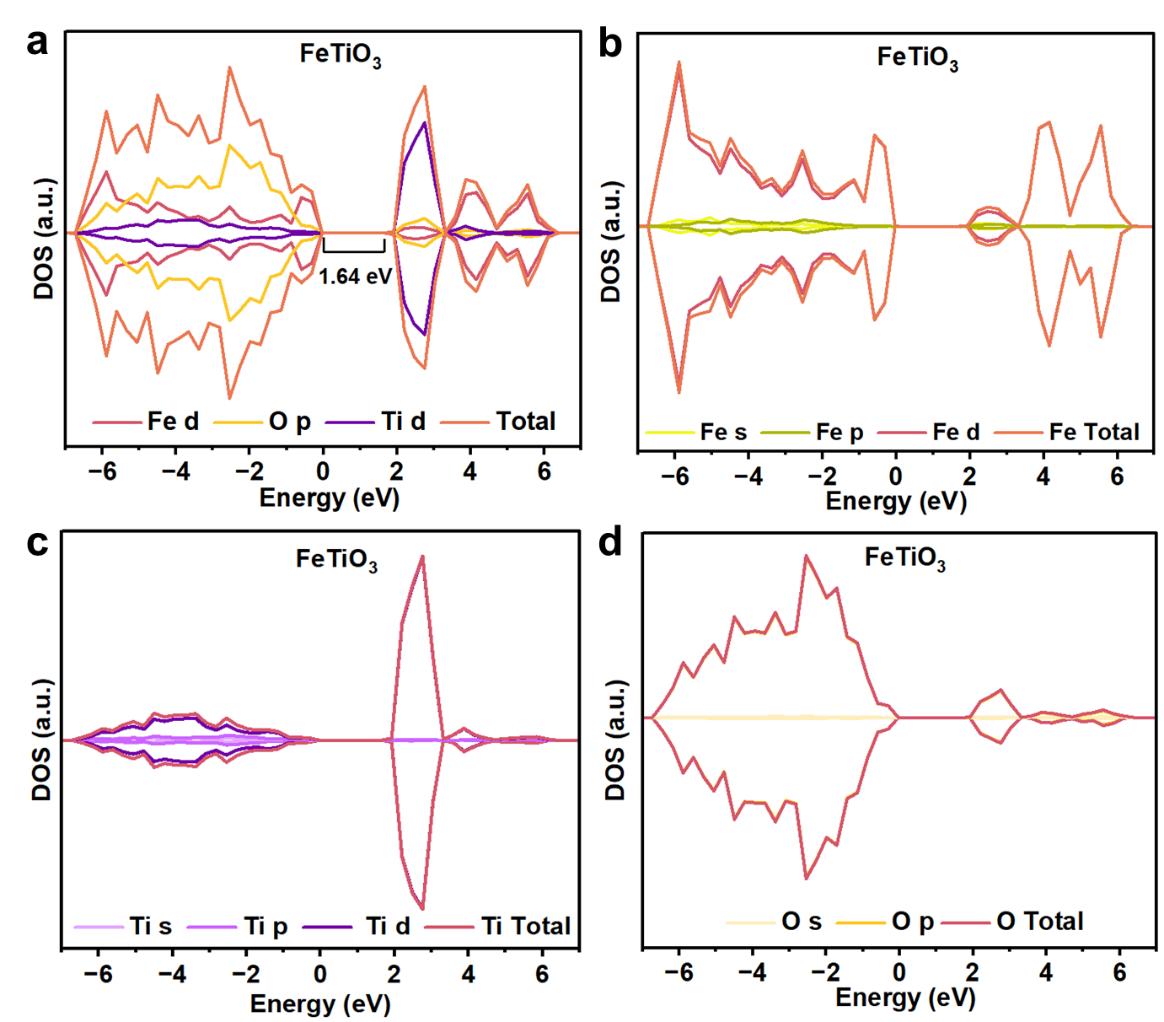


**Supplementary Figure 22.** DOS and PDOS diagrams of FeTiO_3_.


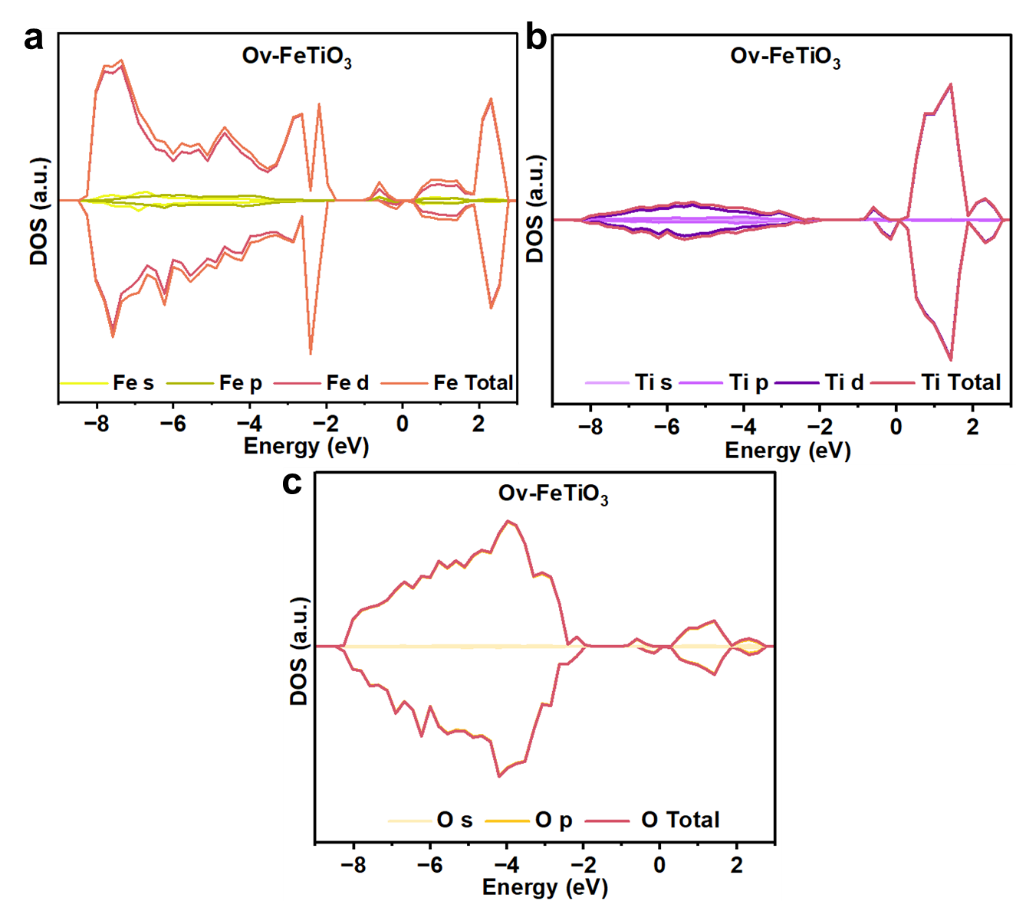


**Supplementary Figure 23.** DOS and PDOS diagrams of Ov-FeTiO_3_.


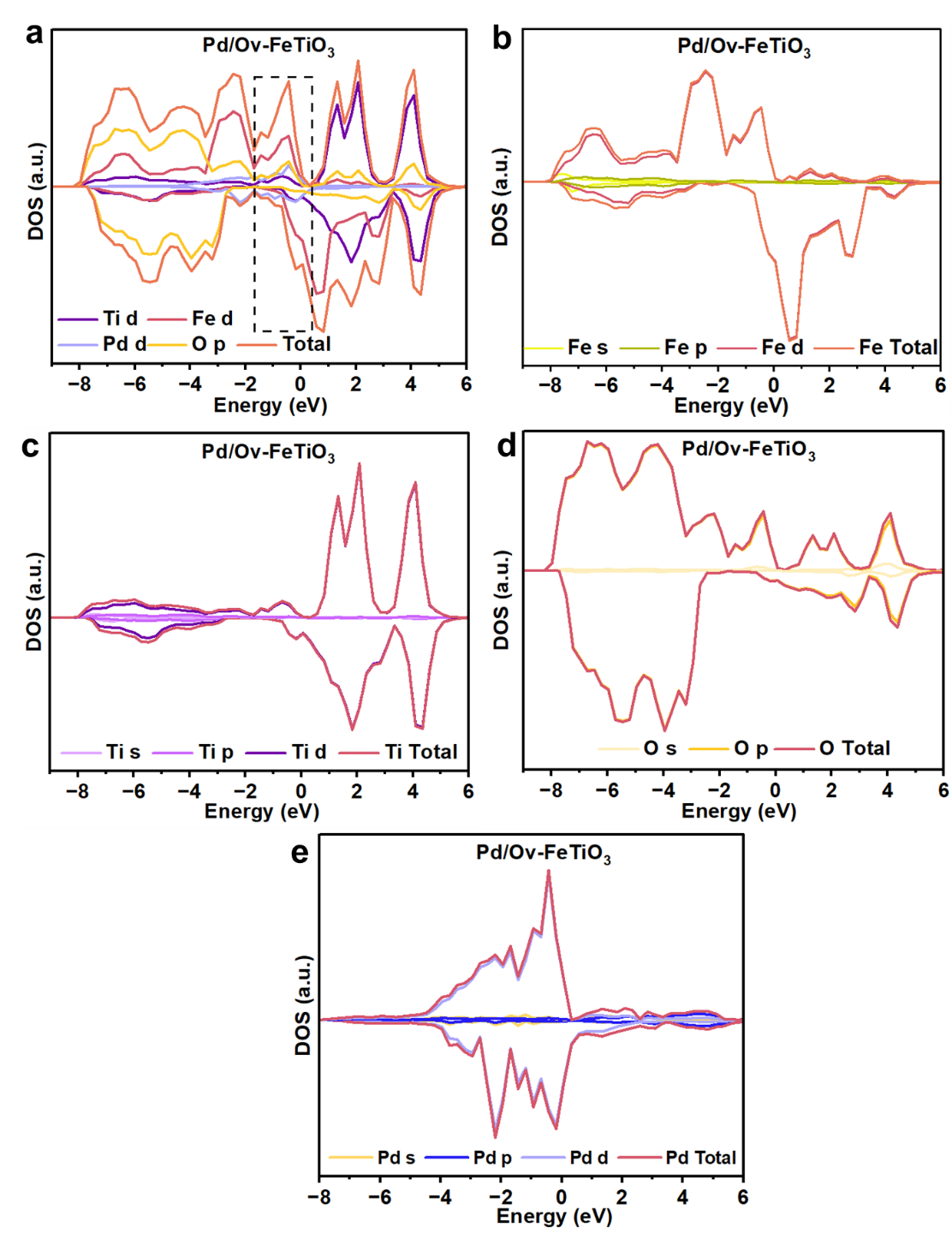


**Supplementary Figure 24.** DOS and PDOS of Pd/Ov-FeTiO_3._


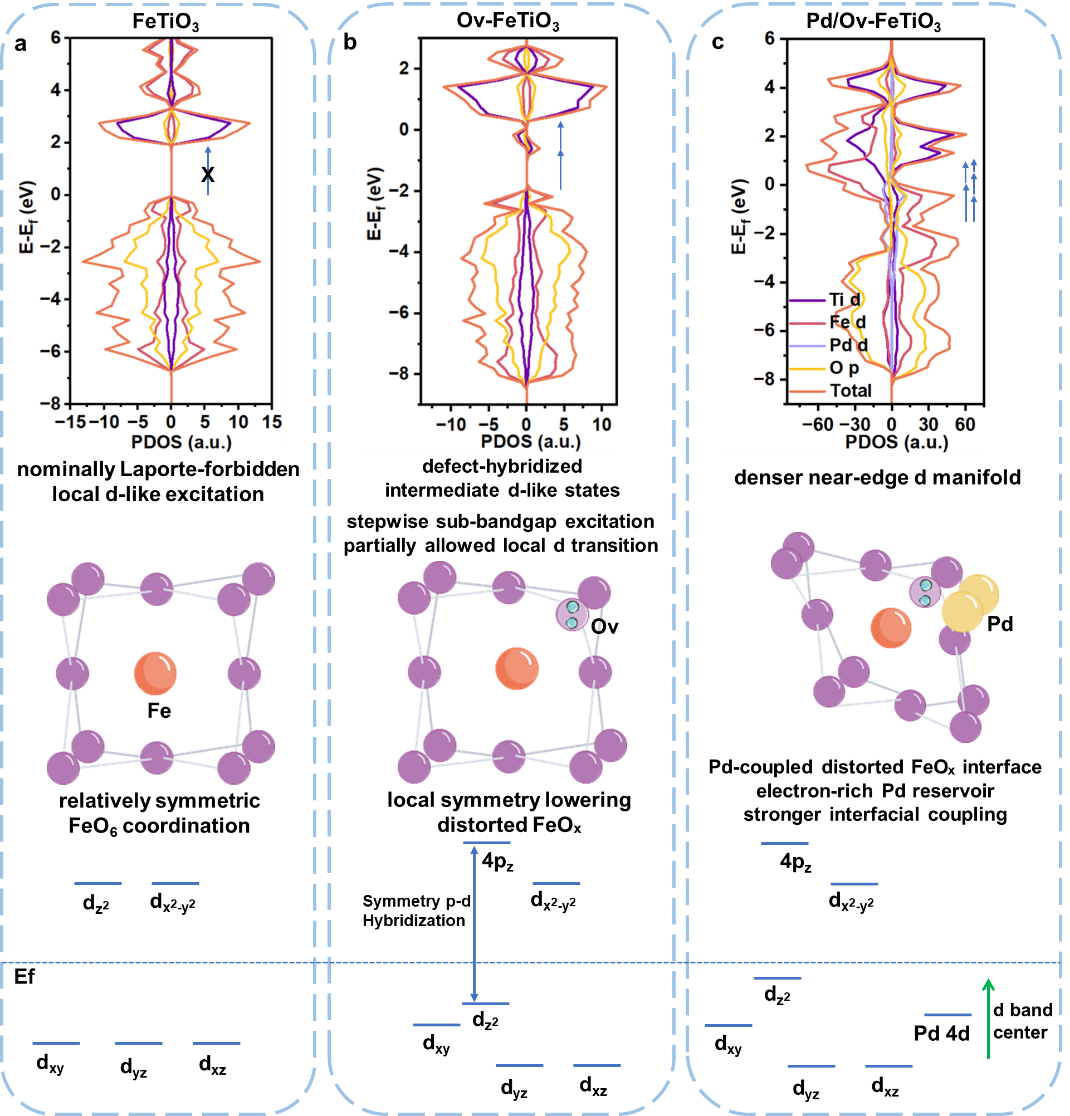


**Supplementary Figure 25.** (a) PDOS of FeTiO_3_, (b) Ov-FeTiO_3_ and (c) Pd/Ov-FeTiO_3_, local coordination environment of Fe-O and molecular orbital arrangement.

**(a) Local confinement (O_h_ crystal field) in intrinsic FeTiO_3_:** As shown in the figure, the Fe sites within the intrinsic crystal are situated in a relatively regular, near-centrosymmetric FeO_6_ octahedral environment (approximately O_h_ point group). In this highly symmetric crystal field, purely local d-d transitions (e.g., t_2g_→e_g_) are strictly forbidden by the Laporte parity conservation law (even-parity to even-parity, g→g). Combined with PDOS results, there is a lack of obvious intermediate states near the

Fermi level in this state, and the system maintains a clear bandgap structure, resulting in a weak infrared response.

**(b) Oxygen Vacancy-Induced Symmetry Breaking and p-d Hybridization (C_4v_ Crystal Field):** Upon introduction of Ov, the neighboring Fe sites collapse from FeO6 to low-coordinate, distorted FeO_x_ (approximately FeO_5_ polyhedra, with local symmetry downgraded to approximately C_4v_ group). The loss of centrosymmetry allows the originally orthogonal even-parity Fe 3d orbitals and odd-parity 4p orbitals to acquire the same irreducible representation (e.g., A_1_ symmetry), resulting in strong p-d orbital hybridization. This distortion-induced mixed parity transforms the originally restricted local d-like transitions into "partially allowed" transitions. This mechanism is strongly supported by Fe K-edge XANES spectra: the significant enhancement and broadening of the white line peak (Fe 1s→4p transition) directly reflects the large amount of unoccupied 4p states and energy level splitting under the low-coordinate distorted crystal field.

**(c) Pd Anchored Triggered Interface Coupling and Sub-Bandgap Excitation (Excitation):** Based on the symmetry breaking, the further loaded Pd is not simply a physical attachment. The d orbitals of Pd undergo strong interfacial electronic coupling with the defect-reconstructed Fe/Ti d states, significantly shifting the local d-band center upwards. The Pd d, Fe/Ti d, and Op states together construct a dense d-like intermediate state (intermediate step) near the Fermi level. This synergistic effect of deep orbital hybridization successfully transforms the originally difficult one-time cross-bandgap excitation into a highly efficient stepwise subbandgap excitation, fundamentally elucidating the physical mechanism of the significant improvement in infrared absorption and photothermal catalytic response of the Pd/Ov-FeTiO_3_ system.


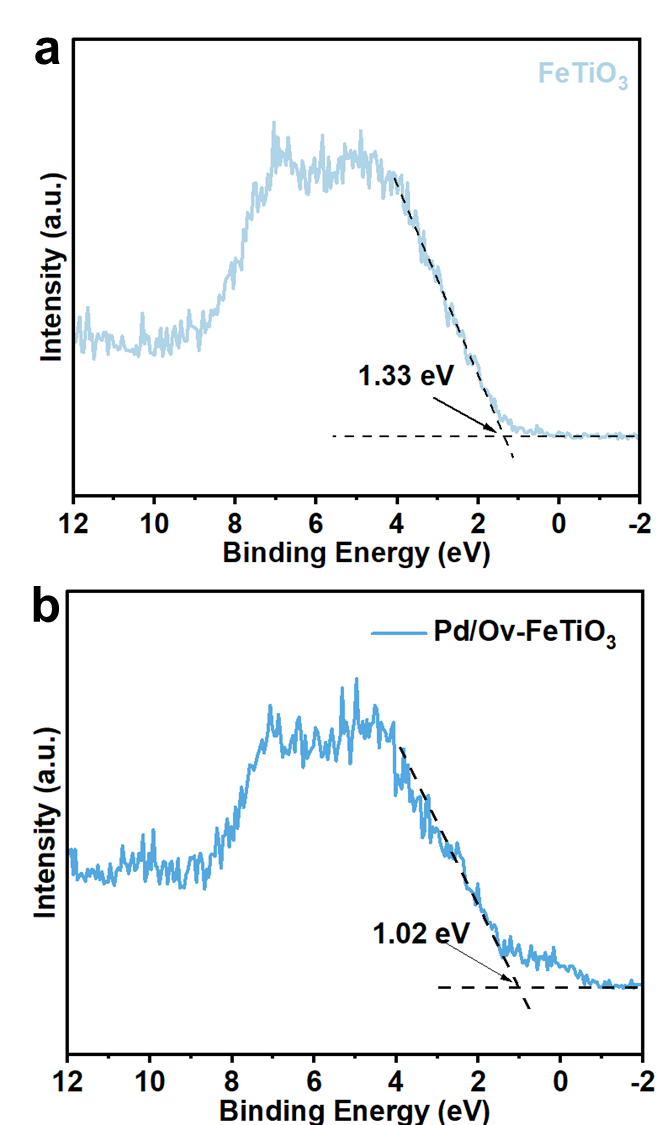


**Supplementary Figure 26.** XPS valence band spectra of FeTiO_3_ and Pd/Ov-FeTiO_3_.


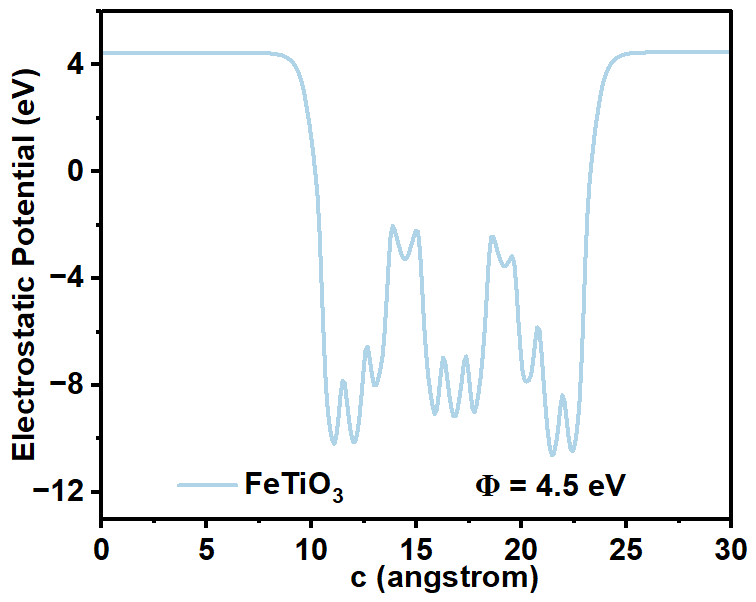


**Supplementary Figure 27.** Work function of FeTiO_3_.


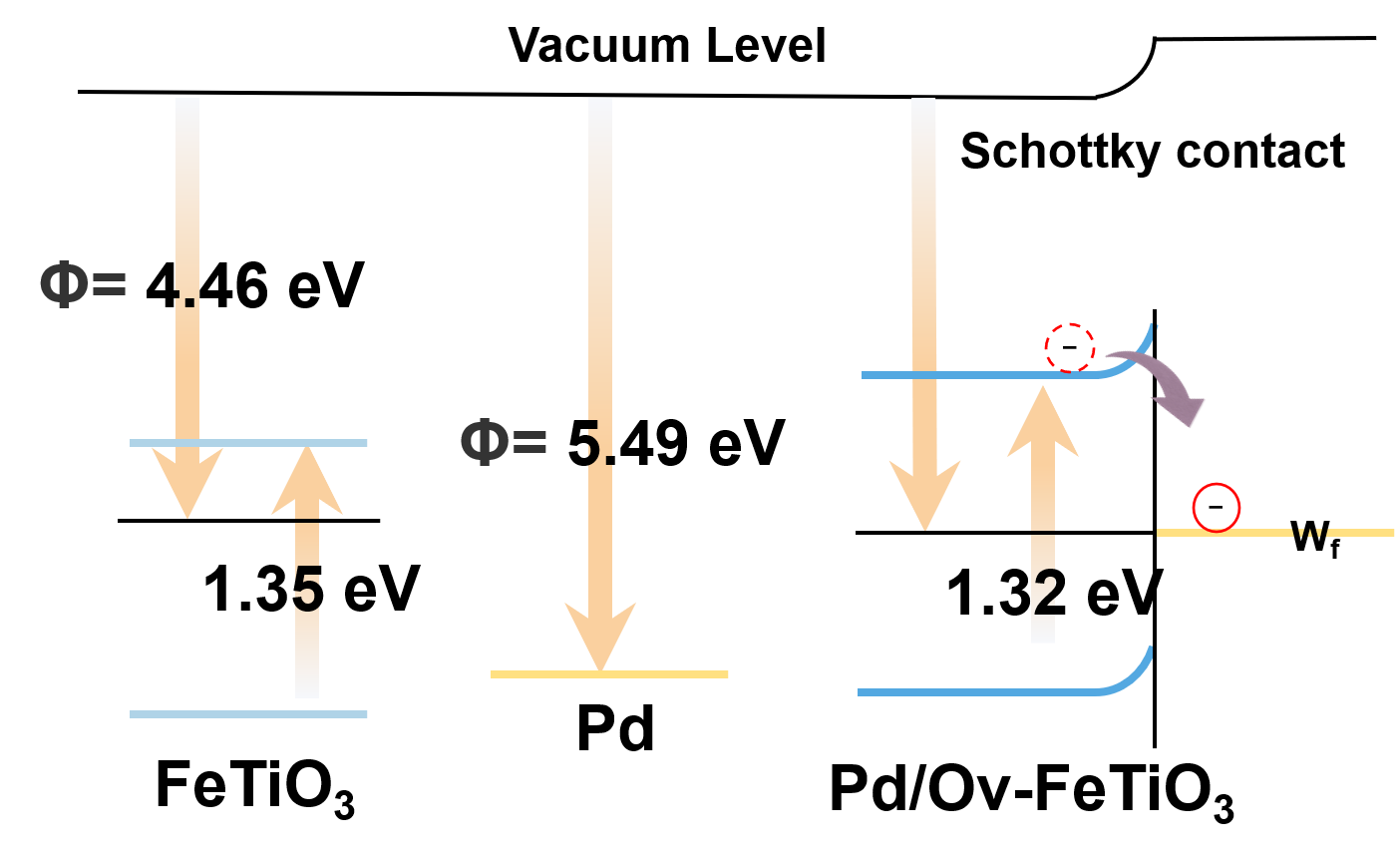


**Supplementary Figure 28.** Energy band structure diagram of FeTiO_3_ and Pd/Ov-FeTiO_3_.

According to the literature, the work function of Pd is about 5.5 ev.^9,10^


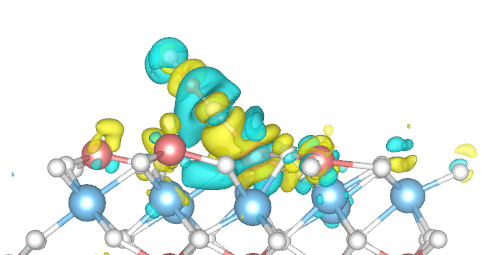


**Supplementary Figure 29.** Differential charge density of CO_2_ adsorbed on FeTiO_3_.


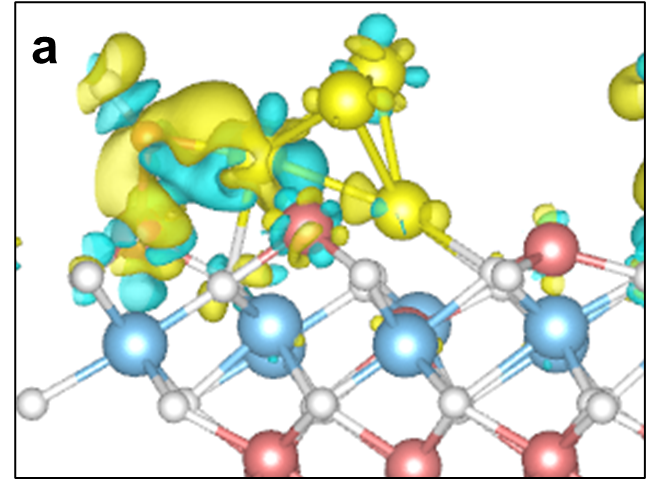


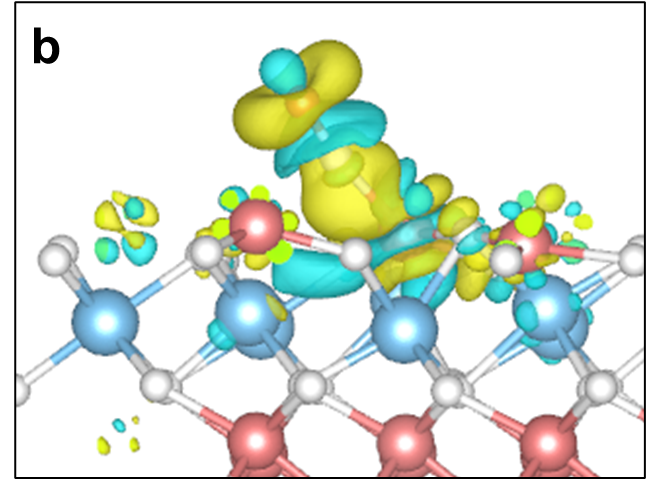


**Supplementary Figure 30.** Differential charge density of CO adsorbed on Pd/Ov-FeTiO_3_ (a) and FeTiO_3_ (b).


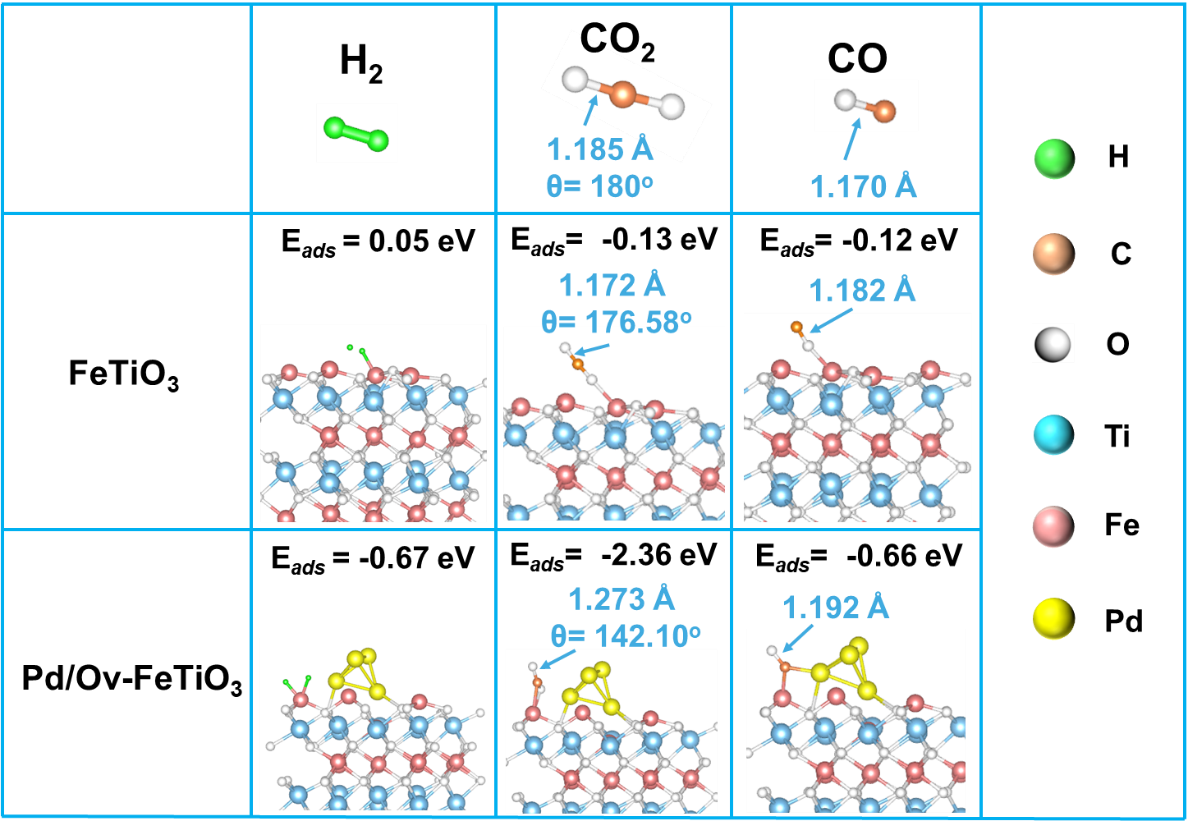


**Supplementary Figure 31.** Adsorption energy and bond length and bond angle of Pd/Ov-FeTiO_3_ and FeTiO_3_ for H_2_, CO_2_, and CO.

The activation performance of different samples was investigated by the bond length and bond angle after CO_2_ and CO adsorption. It was found that Pd/Ov-FeTiO_3_ can better activate CO_2_ and CO.


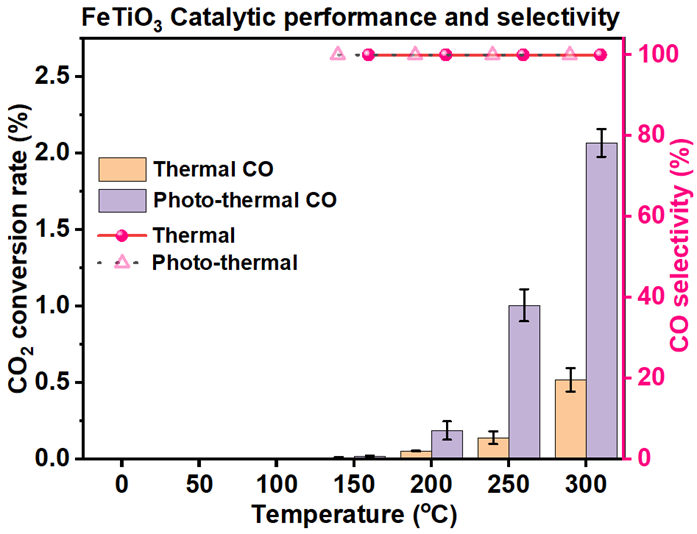


**Supplementary Figure 32.** The yields of CO and the corresponding CO selectivity of FeTiO_3_ at different temperatures.

Interestingly, based on the band structure analysis, the reduction potential of FeTiO₃ is insufficient to reach the redox potentials required for CO and CH₄ formation. As a result, no CO₂ hydrogenation products are observed at room temperature, 50 °C, or 100 °C. However, as the reaction temperature increases, the activation energy barrier decreases, enabling FeTiO₃ to exhibit CO₂ reduction activity at elevated temperatures.


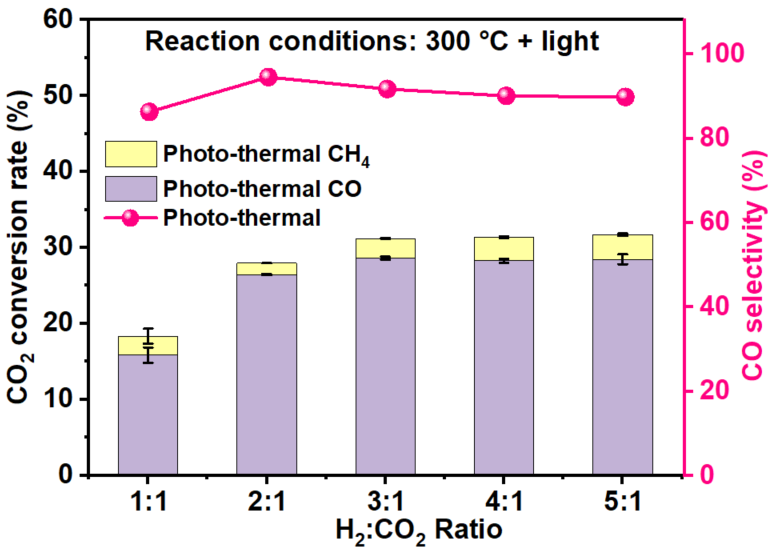


**Supplementary Figure 33.** CO_2_ reduction performance and CO selectivity of Pd/Ov-FeTiO_3_ at 300°C with light for different H_2_ : CO_2_ ratios.


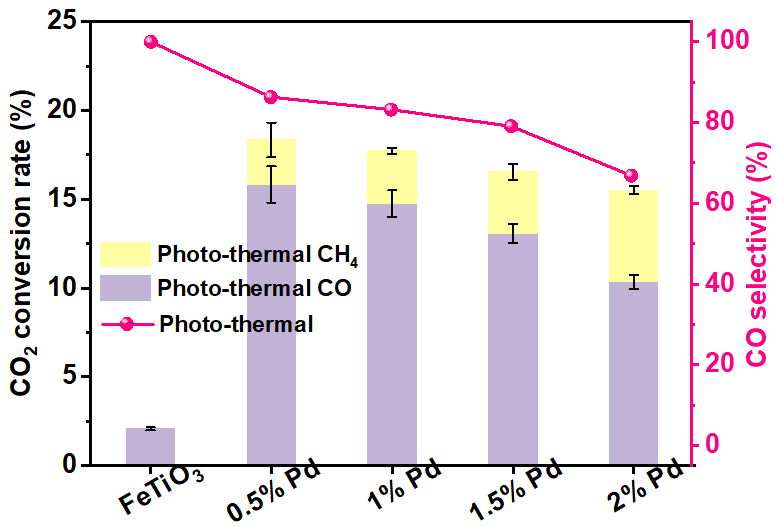


**Supplementary Figure 34.** CO_2_ reduction performance and CO selectivity under different Pd loading ratios.


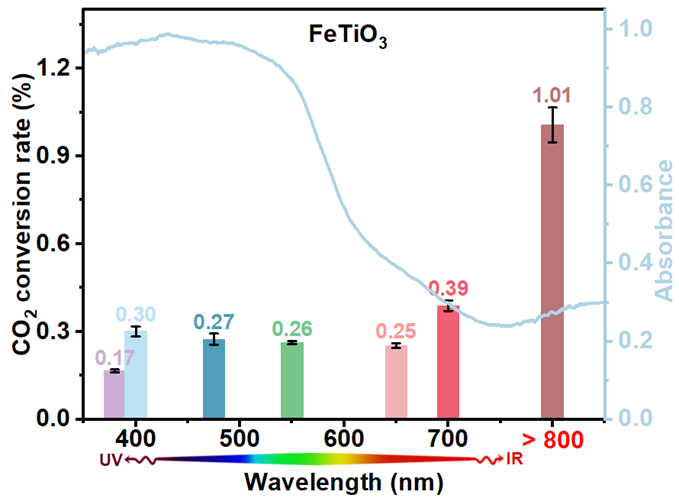


**Supplementary Figure 35.** The product yields of FeTiO_3_ under different monochromatic light irradiation at 300 ^o^C and 15mW·cm^-2^.


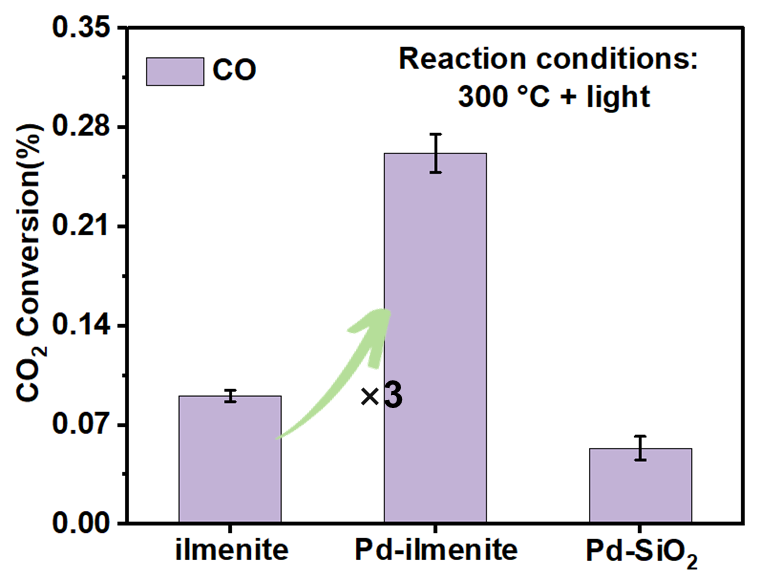


**Supplementary Figure 36.** Performance of different samples after the introduction of Pd.

In order to investigate the effect of Pd introduction on performance improvement, SiO_2_ was used as a carrier and it was found that it only had a trace amount of CO_2_ conversion efficiency. This means that the performance improvement brought by Pd and Ov is also very important to the substrate. The performance improvement of ilmenite surface by introducing Pd is only 3 times, which is far less than the performance improvement of FTO.


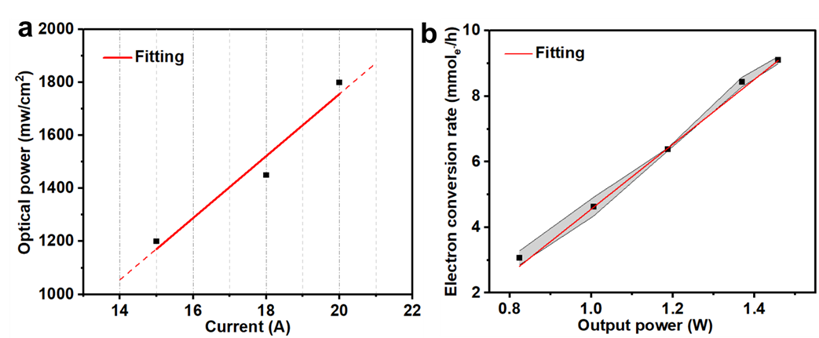


**Supplementary Figure 37.** (a) shows the relationship between the current and light intensity of the light source. (b) shows the electron transfer under different light intensities.


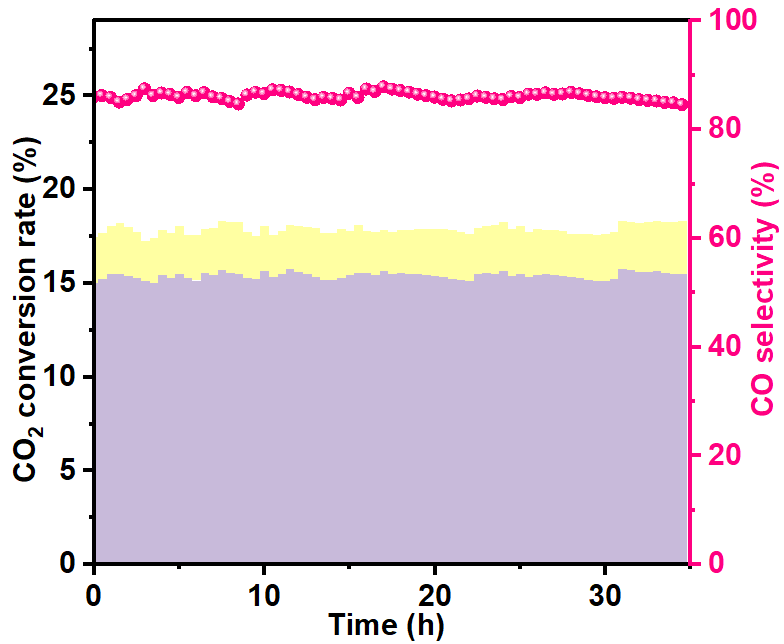


**Supplementary Figure 38.** Stability testing of Pd/Ov-FeTiO_3_.


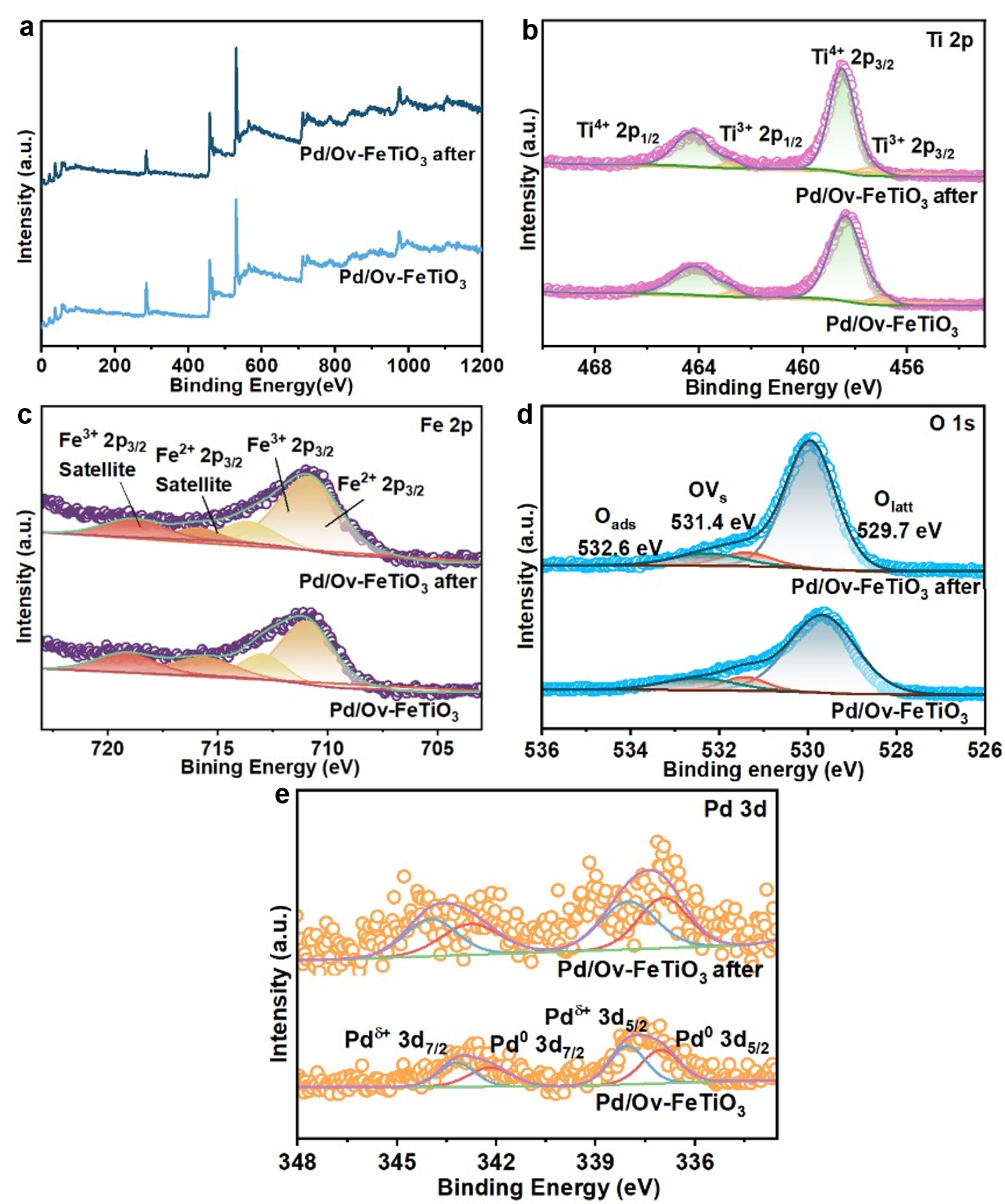


**Supplementary Figure 39.** XPS high-resolution spectra and full spectra of Ti 2p (a), Fe 2p (b), O 1s (c) and Pd 3d (d) of Pd/Ov-FeTiO_3_ before and after reaction.

After more than 40 hours of stability testing, the samples were characterized by XPS. The results showed that there were no significant changes in the XPS spectra of the samples before and after the reaction.


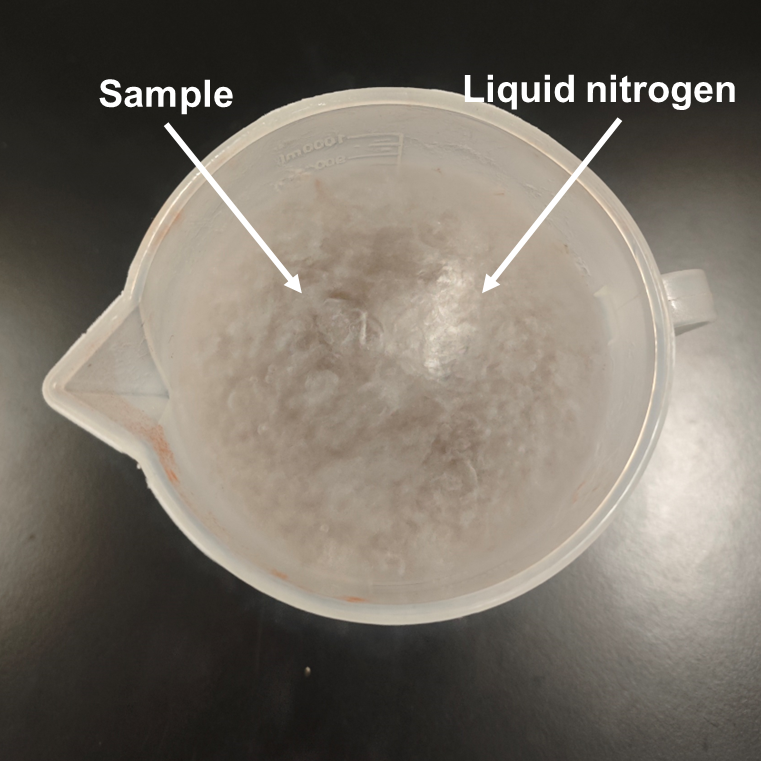


**Supplementary Figure 40.** Catalyst treatment in liquid nitrogen.


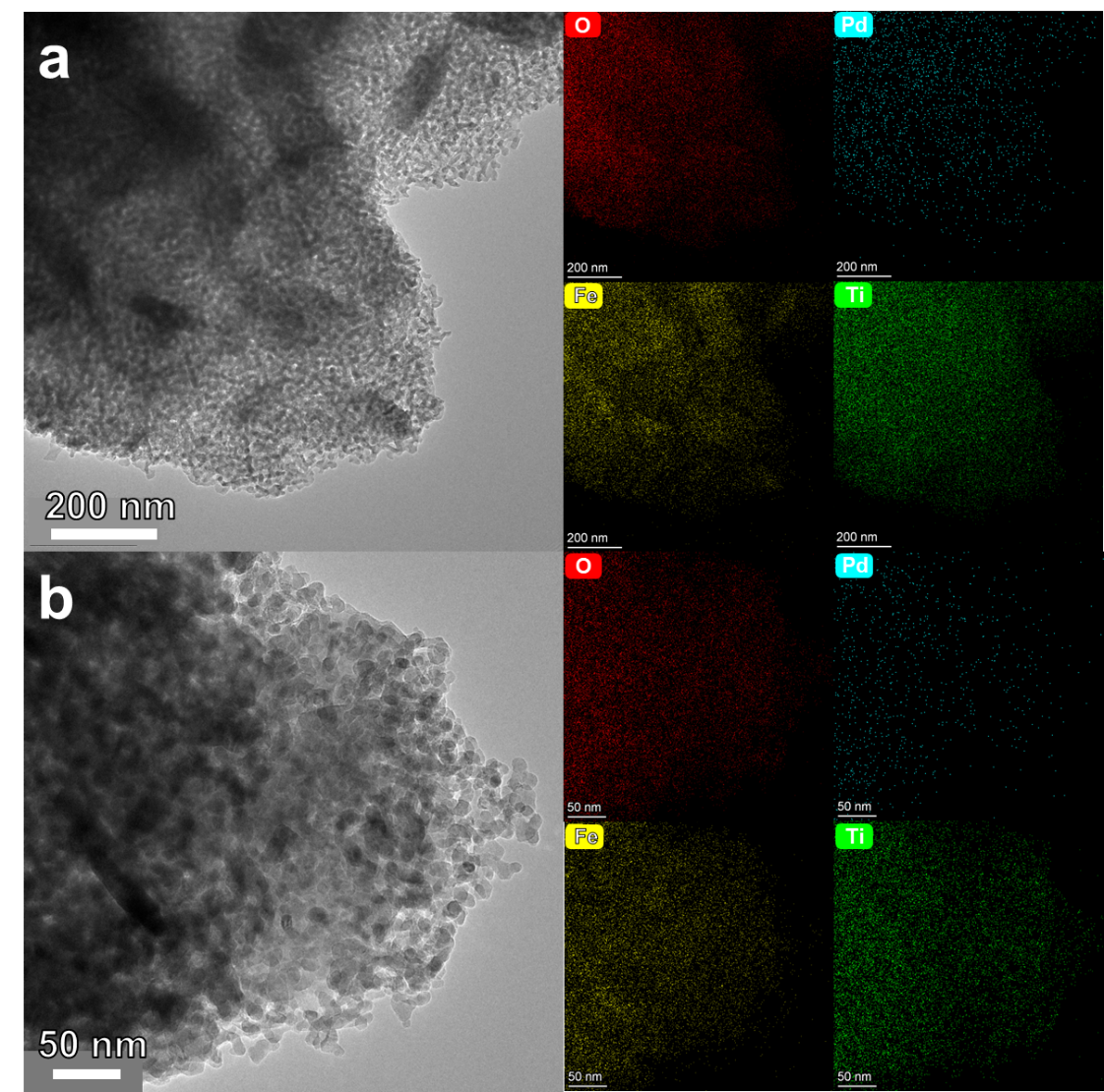


**Supplementary Figure 41.** TEM images after reaction.


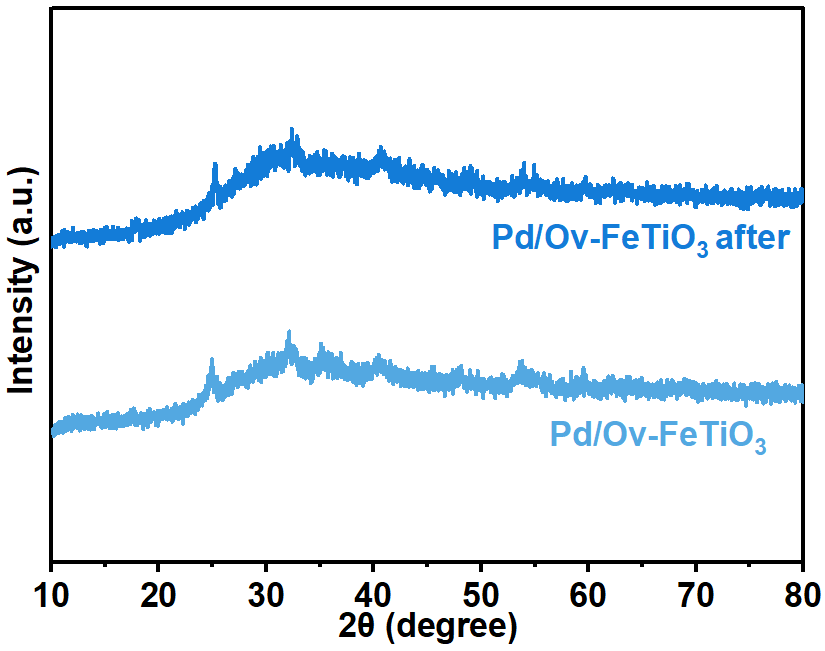


**Supplementary Figure 42.** XRD patterns before and after reaction.

After undergoing cryogenic treatment with liquid nitrogen, the Pd/Ov-FeTiO_3_ catalyst exhibited extremely high stability in both microstructure and crystal structure following CO_2_ reduction performance testing. TEM and XRD characterization results showed no significant difference in the sample before and after the reaction: no aggregation of Pd nanoparticles was observed, and the phase structure in the XRD pattern remained unchanged.


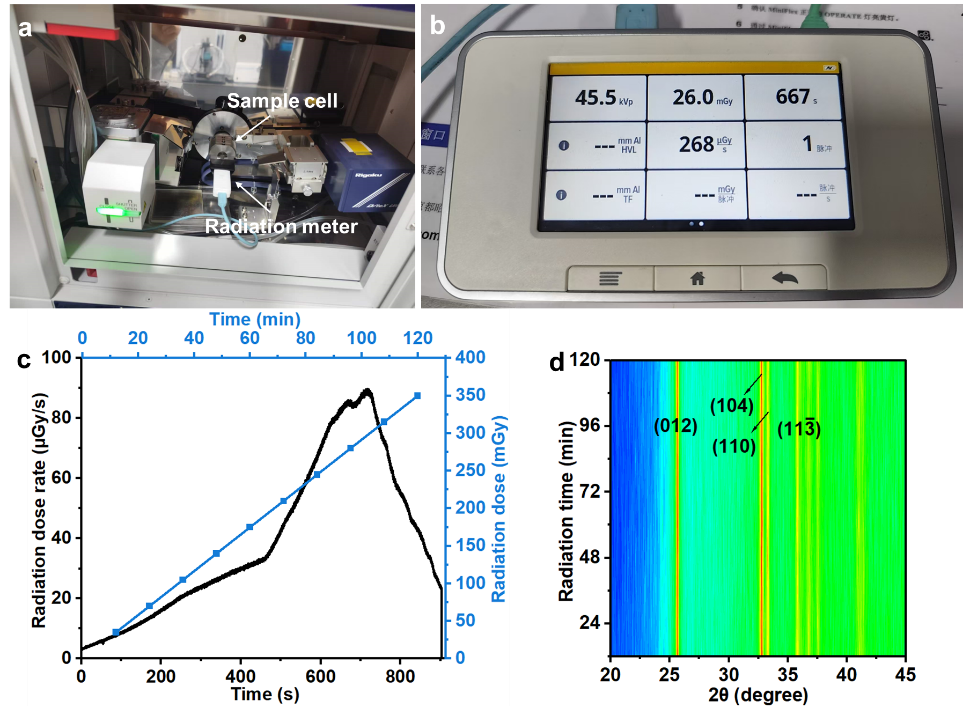


**Supplementary Figure 43.** (a) XRD apparatus and X-ray detector. (b) X-ray power meter. (c) X-ray power received by the sample and estimated cumulative value. (d) XRD pattern of Pd/Ov-FeTiO_3_ after simulated ionization damage caused by high-energy photons.


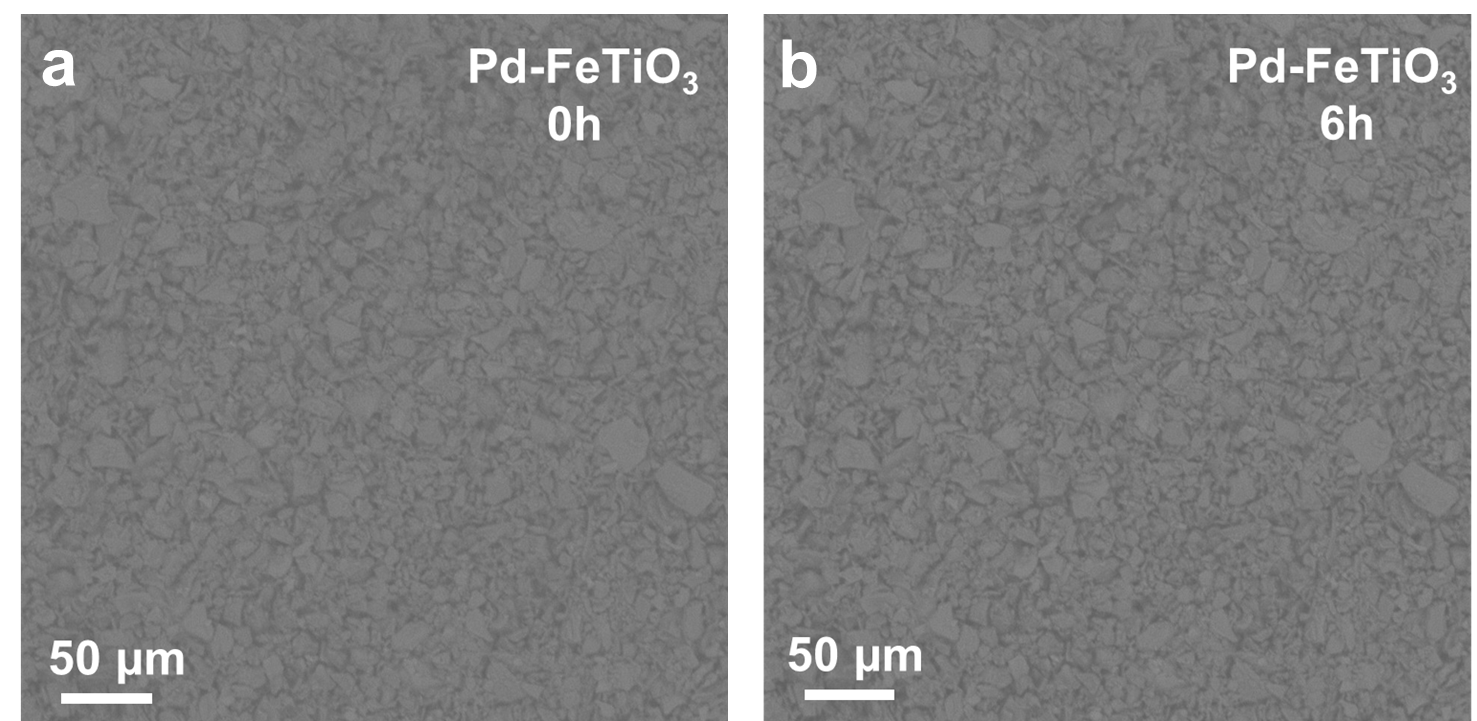


**Supplementary Figure 44.** SEM images before and after 6 hours of high-energy electron irradiation.


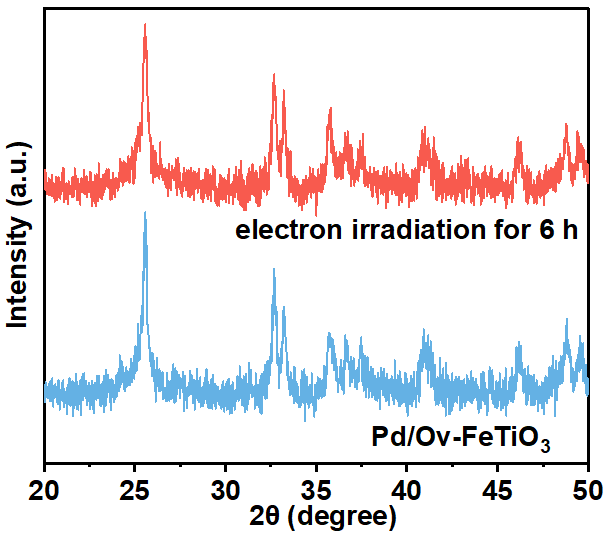


**Supplementary Figure 45.** XRD images before and after 6 hours of high-energy electron irradiation.


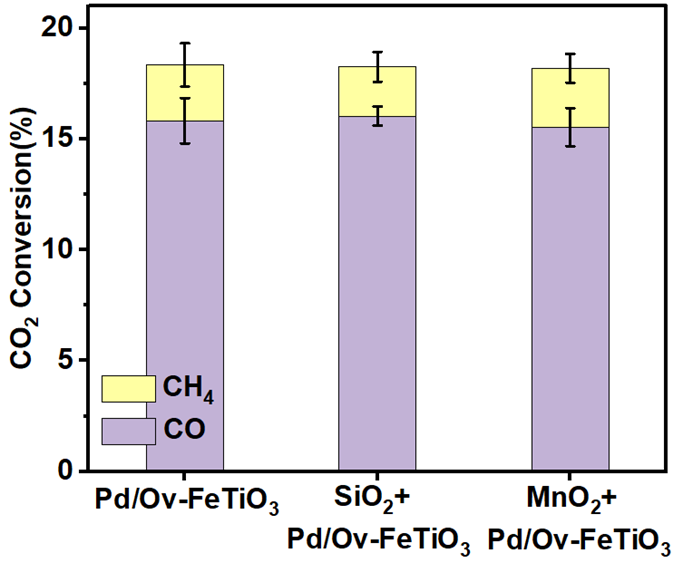


**Supplementary Figure 46.** Performance comparison of SiO_2_ and MnO_2_ mixed with Pd/Ov-FeTiO_3_.

When enriching FeTiO_3_ from lunar soil using processes such as magnetic separation, impurities such as SiO_2_ and MnO_2_ are inevitably introduced. Theoretically, SiO_2_, as an inert support, does not participate in the reaction, while MnO_2_, although possessing the potential to collect holes and promote the photothermal reduction of CO_2_,^11^ is expected to have limited impact due to its low abundance in lunar soil and the difficulty in achieving effective spatial separation of charge carriers through simple physical mixing.^1^ By mixing 5 wt% MnO_2_ and SiO_2_ under the same conditions with Pd/Ov-FeTiO_3_ and conducting tests, the results showed that the presence of both did not significantly affect the overall performance of the catalyst. This result fully confirms that the simple physical mixing of lunar soil-associated impurities does not interfere with the original photothermal catalytic mechanism of Pd/Ov-FeTiO_3_.


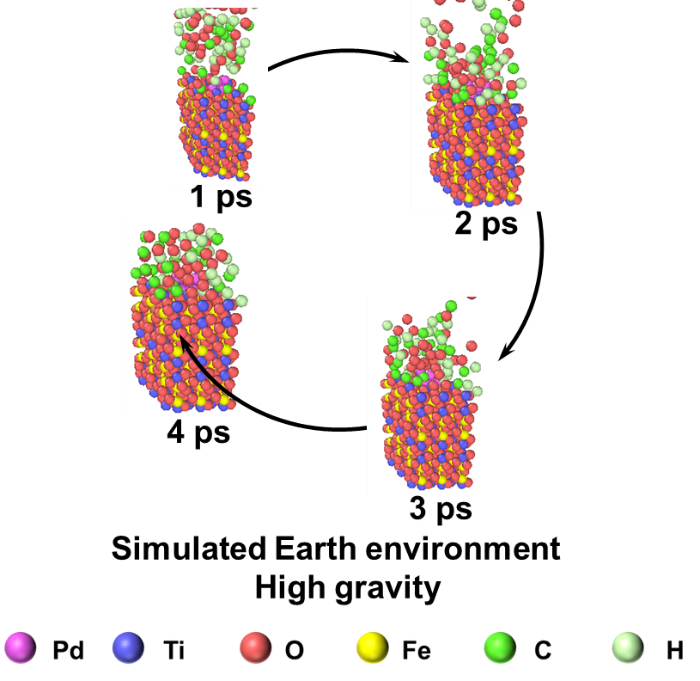


**Supplementary Figure 47.** Molecular dynamics of Pd/Ov-FeTiO_3_.


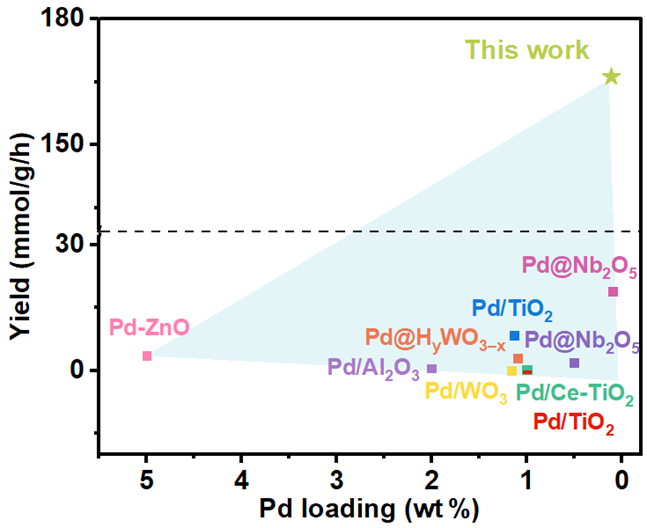


**Supplementary Figure 48.** Comparison of CO_2_ reduction performance of Pd-supported catalysts.


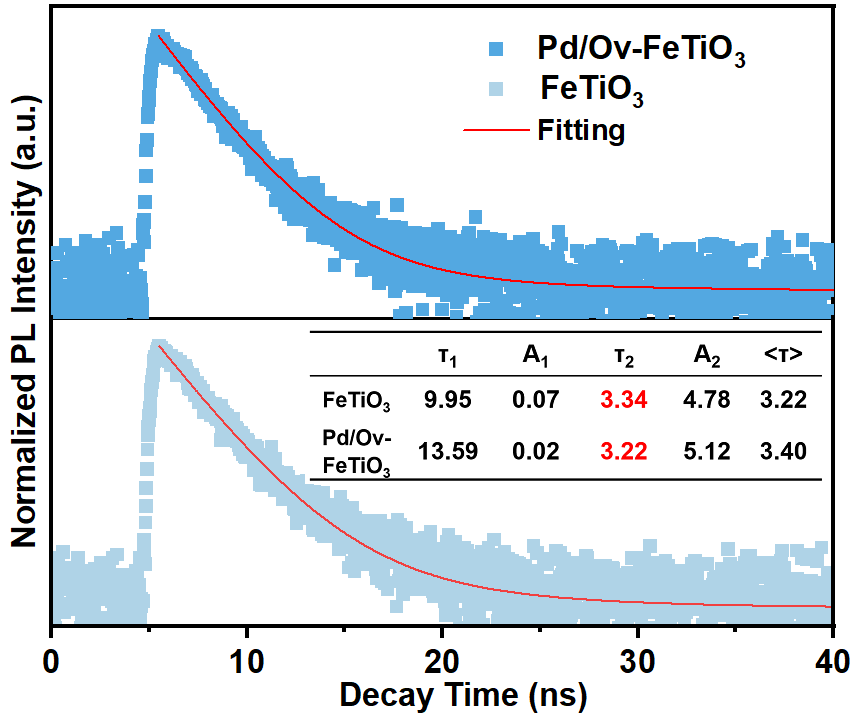


**Supplementary Figure 49**. Transient fluorescence spectra and second-order fitting results.

By performing a second-order fit on the transient fluorescence, the τ_1_ and τ_2_ components represent nonradiative recombination and trapped recombination, respectively. The weighting factor indicates that the τ_2_ component is the dominant process. The introduction of Ov and Pd into FeTiO_3_ reduces τ_2_, implying that electrons can be rapidly trapped by defects in Pd/Ov-FeTiO_3_. This aligns with the conclusion obtained in TA that the shortened electron residence time in the shallow levels indicates their rapid extraction and transfer to the reactants by the electron-rich Pd pool at the interface, providing further evidence for the self-evolving transient interface.


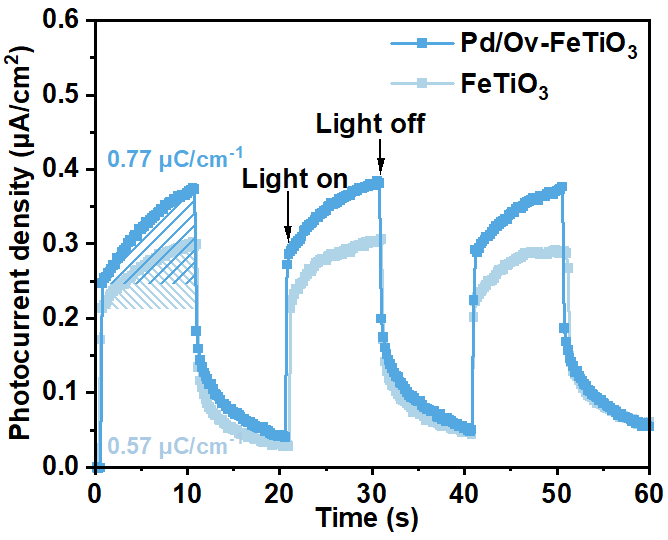


**Supplementary Figure 50.** Photocurrent of FeTiO_3_ and Pd/Ov-FeTiO_3_.


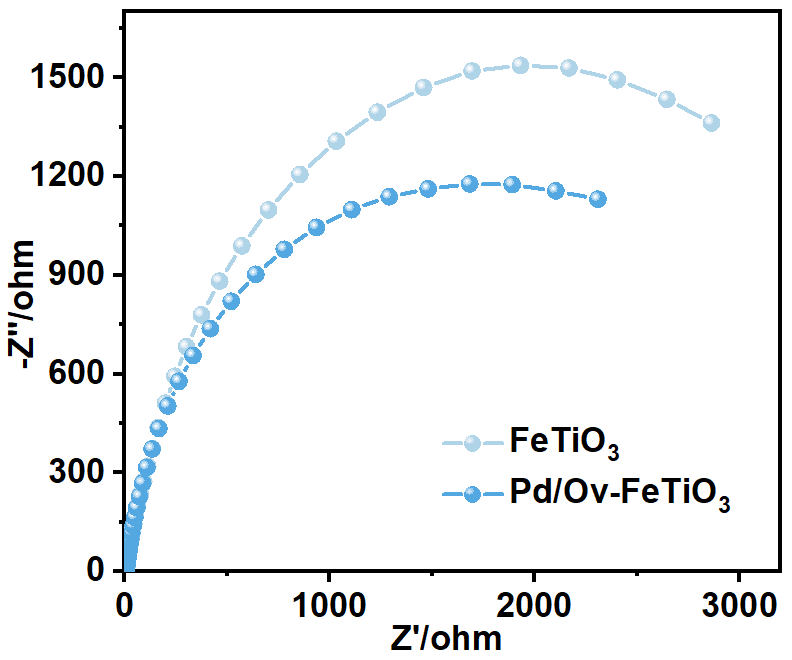


**Supplementary Figure 51.** Impedance spectra of FeTiO_3_ and Pd/Ov-FeTiO_3_.


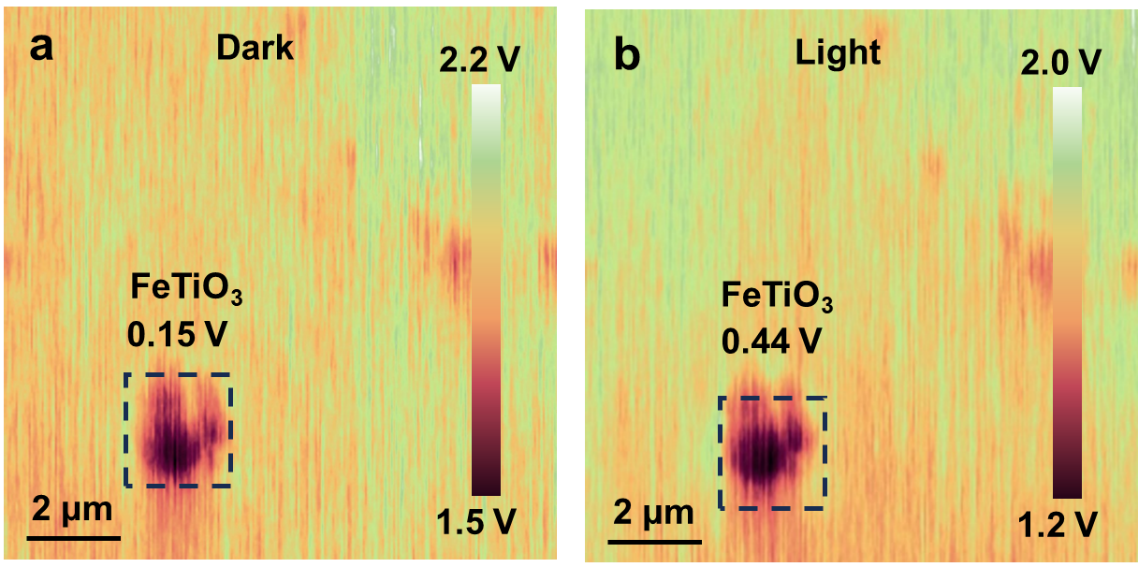


**Supplementary Figure 52.** KPFM images of FeTiO_3_(a, b) in the dark and under light.


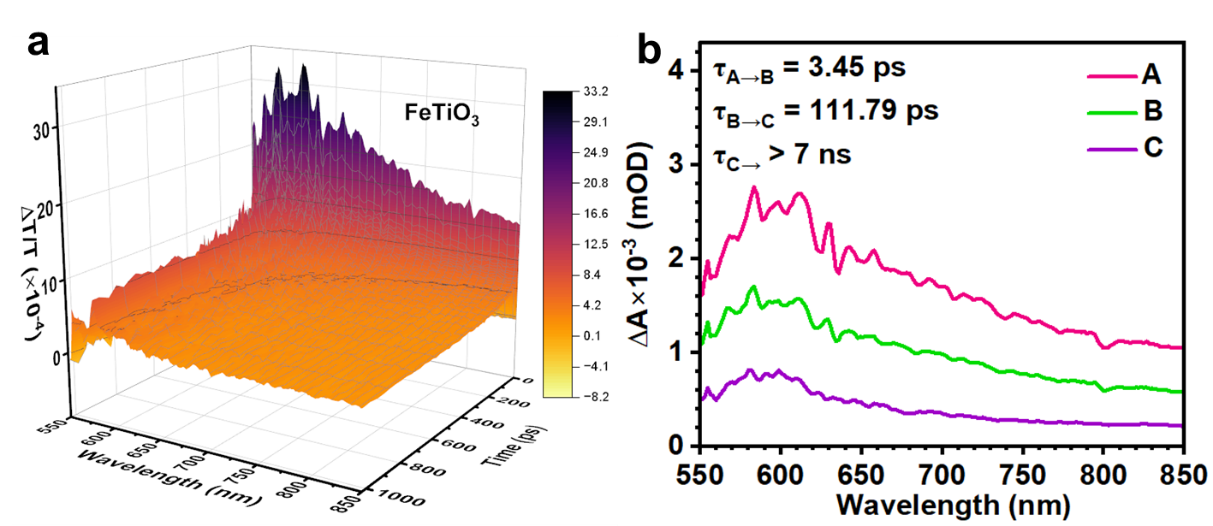


**Supplementary Figure 53.** (a)fs-TA spectra and (b)Globally fitted species-associated spectra (SAS) and time constants of FeTiO_3_.

**
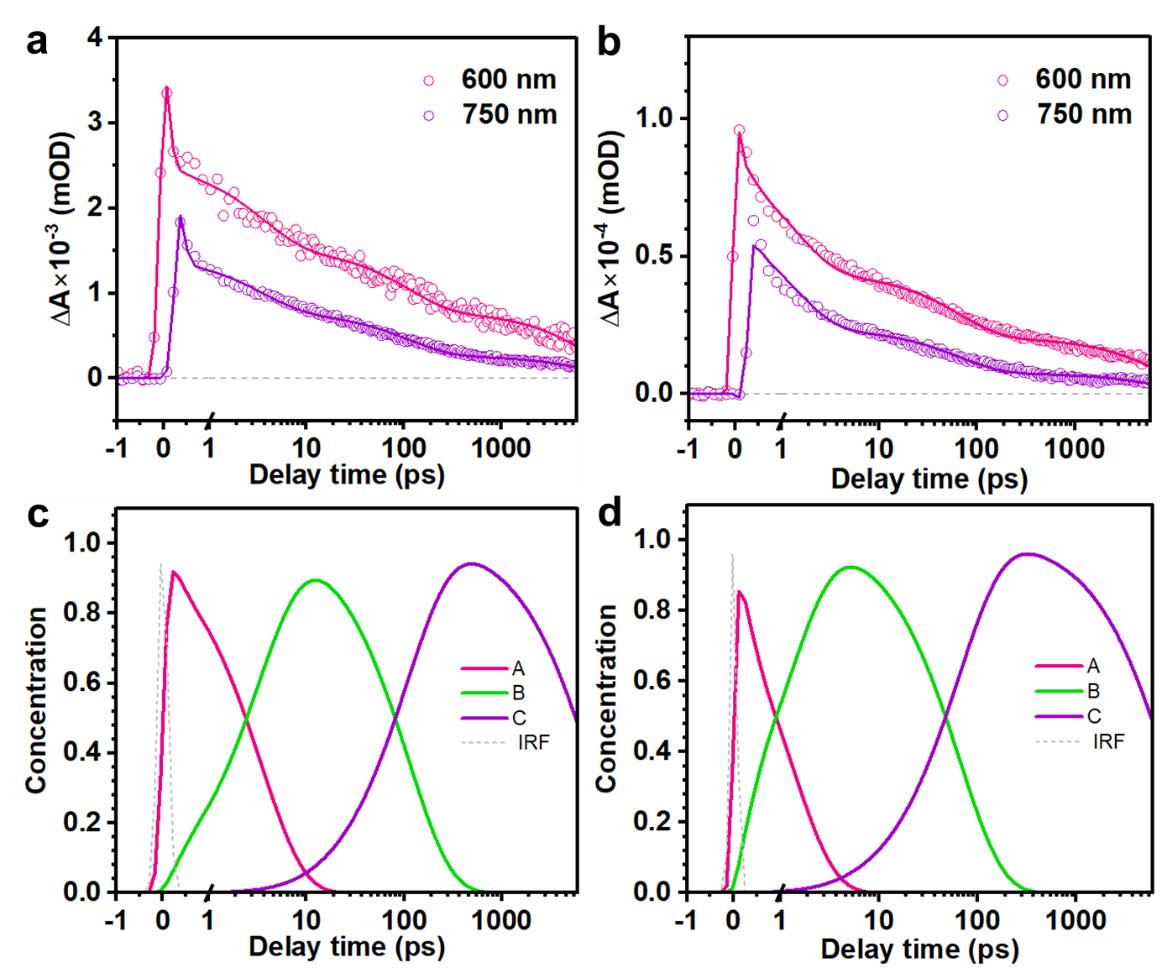
**

**Supplementary Figure 54.** Kinematic comparison of 600/750 nm wavelengths before and after SAS spectral fitting for FeTiO_3_(a) and Pd/Ov-FeTiO_3_(b); Evolution curves of species concentration over time for FeTiO_3_(c) and Pd/Ov-FeTiO_3_(d).

The global fit results show an excellent fit with the original data, which strongly confirms the reliability of the current model construction and the selected parameters. The fitted IRF is the detection limit of the instrument.


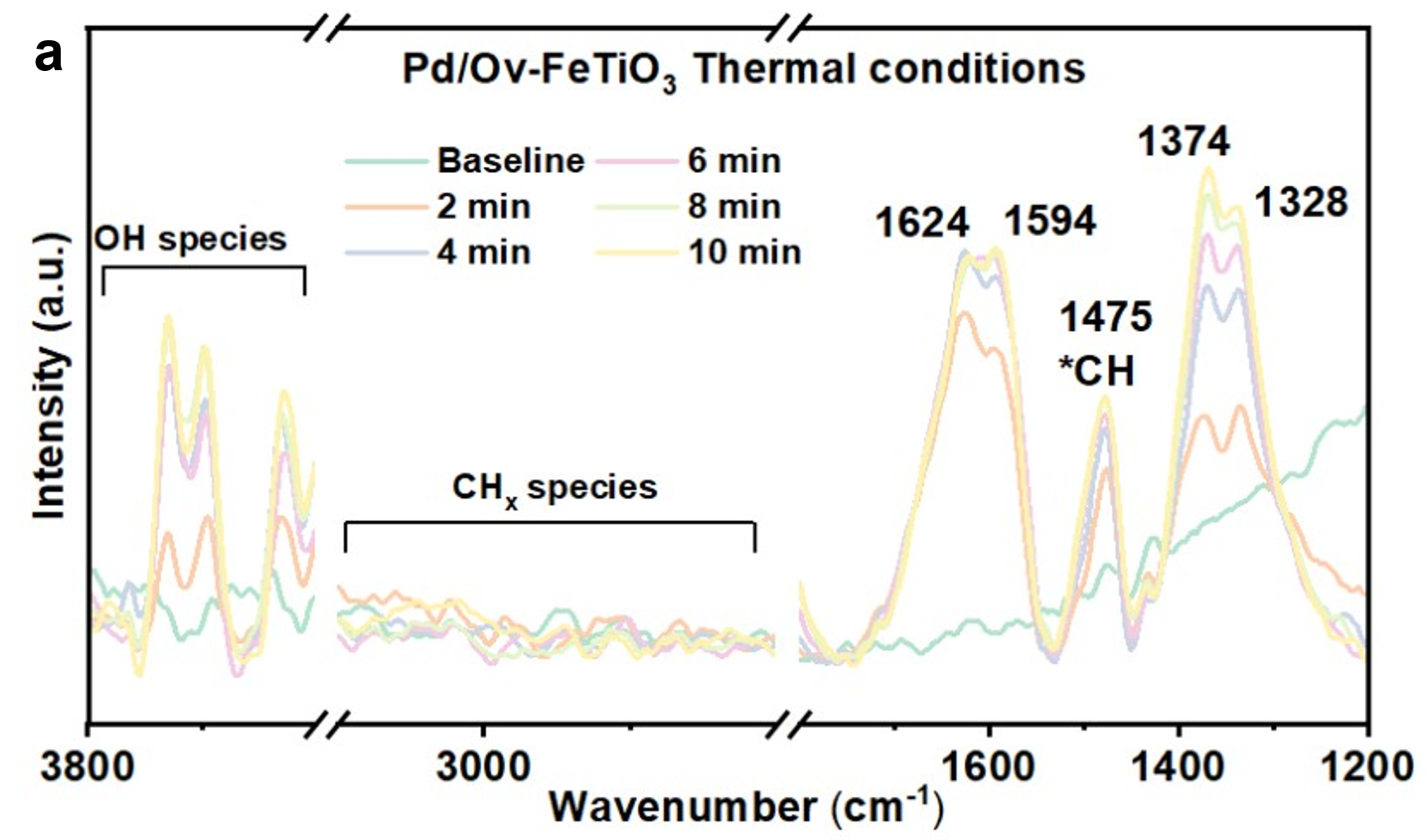


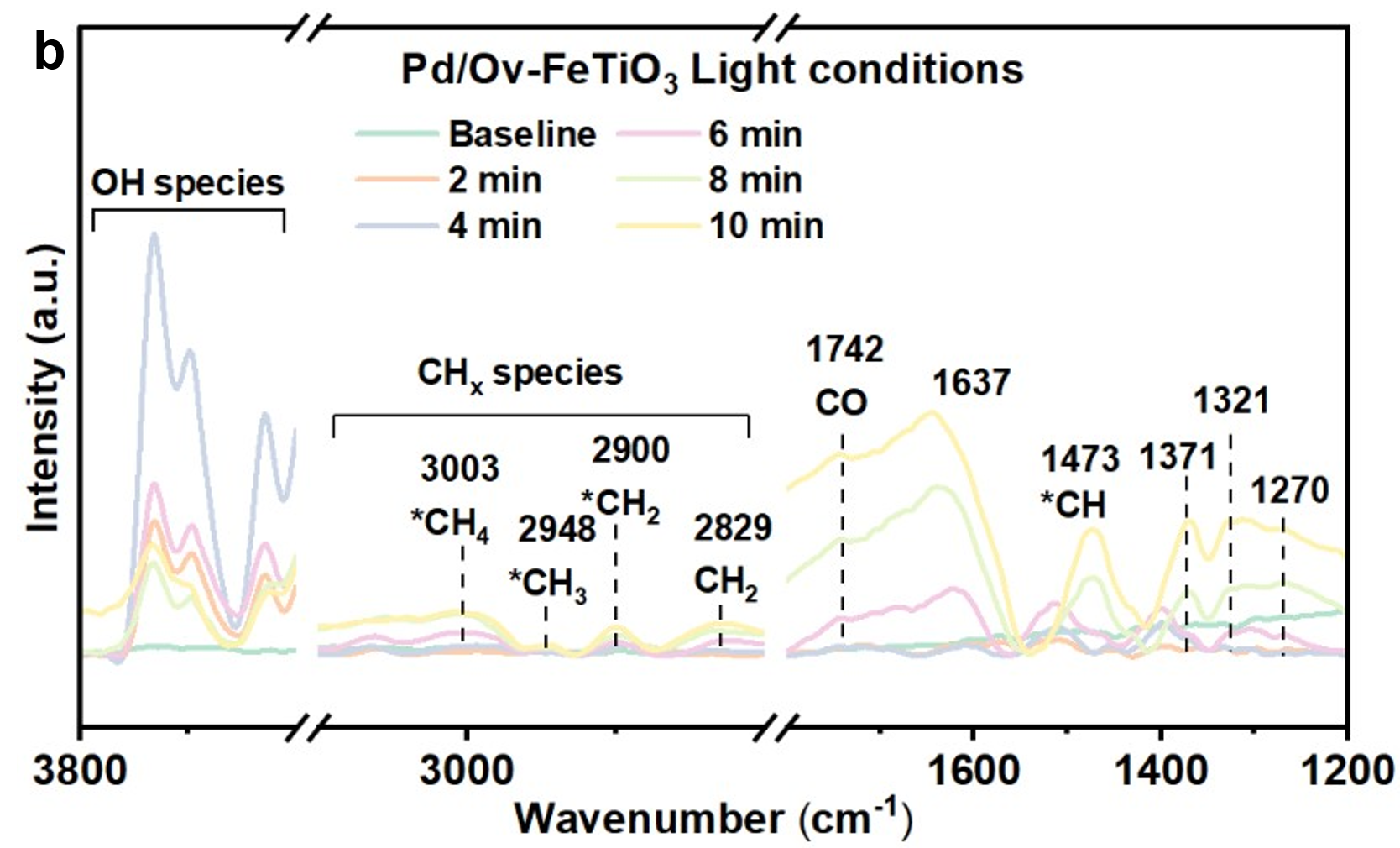


**Supplementary Figure 55.** In-situ infrared spectra of (a) Pd/Ov-FeTiO_3_ under thermal and (b) photo conditions.


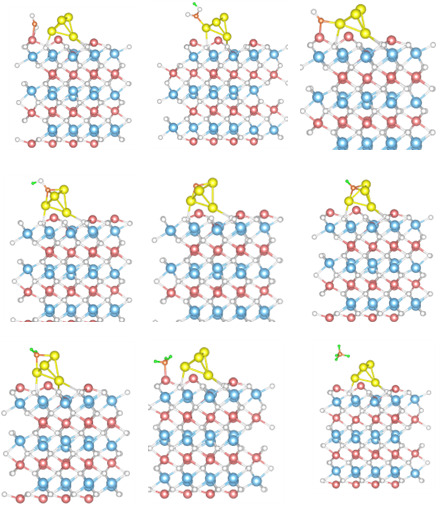


**Supplementary Figure 56.** Model diagram of the reaction path of Pd/Ov-FeTiO_3_.


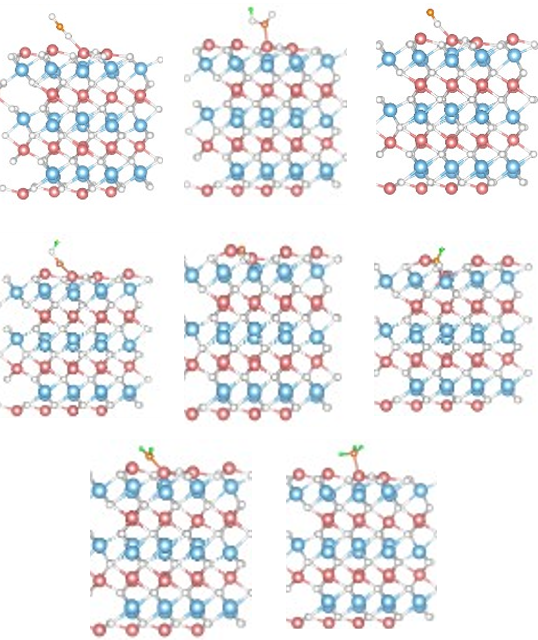


**Supplementary Figure 57.** Model diagram of the reaction path of FeTiO_3_.

**Supplementary** **Table 1.** The characteristics of ilmenite in lunar mineral processing and its technological significance

| **Characteristic** | **Significance for beneficiation and resource utilization** |
| --- | --- |
| Weak ferromagnetism and electrical conductivity | Enables efficient separation and enrichment through magnetic and electrostatic beneficiation techniques. |
| Applicability of magnetic and electrostatic separation | Low operational cost and simple equipment requirements; highly suitable for the Moon’s low-gravity, water-deficient, and vacuum environment; allows for simplified, dry-process flows. |
| Lightweight beneficiation equipment | Minimizes launch mass and associated costs, facilitating on-site beneficiation operations on the lunar surface. |
| Valuable by-products | Ilmenite enrichment yields iron oxides and titanium oxides as by-products, which can be further utilized as feedstock for construction materials. |
| Abundant occurrence in high-Ti basalts of lunar mare regions | Broad spatial distribution enhances accessibility and supports in-situ resource utilization strategies. |
| High overall feasibility | Compared with other mineral separation targets and beneficiation methods, offers lower total operational cost, improved sustainability, and greater overall technical feasibility. |

**Supplementary** **Table 2.** X-ray Photoelectron Spectroscopy Fitting Data

| Samples | Molar ratio | | O_latt_ (%) | O_vac_ (%) | O_ads_  (%) | Molar ratio |
| --- | --- | --- | --- | --- | --- | --- |
|  | Pd^0^/Pd^δ+^ | Fe^2+^/Fe^3+^ |  |  |  | O_vac_/O_latt_ |
| FeTiO_3_ | **—** | 0.61 | 78.13 | 7.81 | 14.06 | 9.99 |
| Pd/Ov-FeTiO_3_ | 1.05 | 0.61 | 78.13 | 8.59 | 13.28 | 10.99 |
| Pd/Ov-FeTiO_3_  after | 1.05 | 0.61 | 80.65 | 8.87 | 10.48 | 10.99 |

**Supplementary** **Table 3.** Fitting parameters of the Fe-refined EXAFS spectra of samples

| **Sample** | **Path** | **N** | **R (Å)** | **ΔE_0_ (eV)** | **σ^2^ (Å^2^)** | **R-factor** |
| --- | --- | --- | --- | --- | --- | --- |
| Fe foil | Fe–Fe | 8.0 | 2.44 | 2.64 | 0.006 | 0.014 |
| FeO | Fe–O | 6.0 | 2.11 | -3.26 | 0.013 | 0.010 |
|  | Fe–O–Fe | 12.0 | 3.07 |  | 0.013 |  |
| Fe_2_O_3_ | Fe–O | 4.0 | 1.96 | -7.28 | 0.013 | 0.012 |
|  | Fe–O–Fe | 4.0 | 3.00 |  | 0.009 |  |
| Fe_3_O_4_ | Fe–O | 3.0 | 1.99 | -6.75 | 0.017 | 0.008 |
|  | Fe–O–Fe | 2.0 | 3.00 |  | 0.009 |  |
| FeTiO_3_ | Fe–O | 3.0 | 1.92 | -8.40 | 0.016 | 0.025 |
|  | Fe–O–Ti | 1.0 | 2.67 |  | 0.009 |  |
| Pd/Ov-FeTiO_3_ | Fe–O | 3.0 | 1.95 | -5.78 | 0.008 | 0.024 |
|  | Fe–O–Ti | 3.0 | 3.08 |  | 0.024 |  |

(N: coordination number; R: distance; E0: energy shift; σ^2^: mean-square disorder; R-factor: Errors in data fitting).

**Supplementary** **Table 4.** Fitting parameters of the Ti-refined EXAFS spectra of samples

| **Sample** | **Path** | **N** | **R (Å)** | **ΔE_0_ (eV)** | **σ^2^ (Å^2^)** | **R-factor** |
| --- | --- | --- | --- | --- | --- | --- |
| Ti foil | Ti–Ti | 8.0 | 2.90 | 4.99 | 0.007 | 0.014 |
| TiO_2_ | Ti–O | 2.0 | 1.93 | -6.48 | 0.009 | 0.013 |
|  | Ti–O–Ti | 4.0 | 3.69 |  | 0.017 |  |
| Pd/Ov-FeTiO_3_ | Ti–O | 6.0 | 1.91 | -5.99 | 0.010 | 0.019 |
|  | Ti–O–Fe | 2.0 | 3.10 |  | 0.006 |  |

(N: coordination number; R: distance; E0: energy shift; σ2: mean-square disorder; R-factor: Errors in data fitting).

**Supplementary** **Table 5.** In situ X-ray Photoelectron Spectroscopy Fitting Data

| Samples | Molar ratio | | O_latt_ (%) | O_vac_ (%) | O_ads_  (%) | Molar ratio |
| --- | --- | --- | --- | --- | --- | --- |
|  | Pd^0^/Pd^δ+^ | Fe^2+^/Fe^3+^ |  |  |  | O_vac_/O_latt_ |
| Dark | 1.05 | 0.61 | 81.97 | 9.02 | 9.01 | 9.9 |
| Light | 1.49 | 0.66 | 86.96 | 8.70 | 4.34 | 9.1 |
| Light+300 °C | 1.61 | 0.90 | 87.72 | 7.9 | 4.38 | 8.3 |

**Supplementary** **Table 6.** Ea activation energy fitting data

|  | A | k | Ea (kJ/mol) |
| --- | --- | --- | --- |
| FeTiO_3_  Photo-thermal | -2.33 | 7.88 | 65.49 |
| Pd/Ov-FeTiO_3_  Photo-thermal | -12.82 | 1.03 | 8.55 |
| Pd/Ov-FeTiO_3_  Thermal | -4.24 | 6.32 | 52.56 |

**Supplementary** **Table 7.** Comparison of the output of this work with other work

| No. | Sample | Temperature (^o^C) | Yield | References |
| --- | --- | --- | --- | --- |
| 1 | Pd-FeTiO_3_ | 300 | 166.16 mmol/g/h | This Work |
| 2 | Ti-MOF | 400 | 4944.81 μmol/g/h | 12 |
| 3 | ZrO_2_/ZnS | 96 | 9.4 μmol/g/h | 13 |
| 4 | CuTCPP/MXene/TiO_2_ | 25 | 124 μmol/g/h | 14 |
| 5 | CBB/MoS_2_ | 117 | 172.79 μmol/g/h | 15 |
| 6 | α-Fe_2_O_3_@NiO_x_ | 25 | 199.3 μmol/g/h | 16 |
| 7 | ZnIn_2_S_4_/Cu_x_O | 5 | 1579.4 μmol/g/h | 17 |
| 8 | ZnIn_2_S_4_@Ni(OH)_2_/NiO | 92 | 133.74 μmol/g/h | 18 |
| 9 | IO-B-TiO_2_/In_2_O_3_ | 178.7 | 251.25 μmol/g/h | 19 |
| 10 | LaNiO_3_ | 200 | 38 mmol/g/h | 20 |
| 11 | Ru/TiO_2_ | 550 | 8.7 mmol/g/h | 21 |
| 12 | NiO/Co_3_O_4_/Fe_2_O_3_ | 200 | 2.9 mmol/g/h | 22 |
| 13 | Ru-TiO_x_ | 259.3 | 15.84 mmol/g/h | 23 |
| 14 | Ni_1_Mo_1_ NPs | 340 | 71.1 mmol/g/h | 24 |
| 15 | Ba_0.9_Ti_0.9_Ni_0.1_O_3-δ_ | 400 | 60.12 mmol/g/h | 25 |
| 16 | Gd_2_O_3_&Co_3_O_4_ | 45 | 4.14 mmol/g/h | 26 |
| 17 | Ni/N_5.0_-CeO_2_ | 325 | 20.9 mmol/g/h | 27 |
| 18 | Co-Ce-Sludge | 300 | 3039.81 μmol/g/h | 28 |
| 19 | Ru-Ni TiO_2_ | 250 | 5 mmol/g/h | 29 |
| 20 | Co@CoN&C | 518 | 132 mmol/g/h | 30 |
| 21 | CuGa/CeO_2_ | 320 | 111.2 mmol/g/h | 31 |
| 22 | CuSiO/CuO_x_ | 500 | 40.4 mmol/g/h | 32 |

**Supplementary** **Table 8. Performance comparison of Pd-supported catalysts in different literature**

| No. | Sample | Pd loading  (wt %) | Yield  (mmol /g_cat_/h) | References |
| --- | --- | --- | --- | --- |
| 23 | Pd/Ov-FeTiO_3_ | 0.11 | 166.16 | This Work |
| 24 | Pd@Nb_2_O_5_ | 0.1 | 18.8 | 33 |
| 25 | Pd@Nb_2_O_5_ | 0.5 | 1.8 | 33 |
| 26 | Pd/TiO_2_ | 1.13 | 8.47 | 34 |
| 27 | Pd/TiO_2_ | 1 | 0.02 | 35 |
| 28 | Pd/Ce-TiO_2_ | 1 | 0.25 | 36 |
| 29 | Pd/Al_2_O_3_ | 2.0 | 0.53 | 37 |
| 30 | Pd/WO_3_ | 1.16 | 0.08 | 38 |
| 31 | Pd@H*_y_*WO_3–_*_x_* | 1.1 | 3.0 | 39 |
| 32 | Pd-ZnO | 5 | 3.6 | 40 |

**Supplementary** **Table 9.** Transient fluorescence biexponential fitting data

|  | τ1 | A1 | τ2 | A2 | <τ> |
| --- | --- | --- | --- | --- | --- |
| FeTiO_3_ | 9.95 | 0.07 | 3.34 | 4.78 | 3.22 |
| Pd/Ov-FeTiO_3_ | 13.59 | 0.02 | 3.22 | 5.12 | 3.40 |

**Supplementary** **Table 10.** Frequency and distribution of intermediates on different samples

| ν (cm^-1^) | Assignment | References |
| --- | --- | --- |
| 1441, 1473,1475, 1479 | *CH | 42 |
| 1726, 1742 | CO | 43 |
| 1270, 1371, 1374,  1594, 1595 | COOH | 44 |
| 2829, 2837, 2984, 2900 | *CH_2_ | 42 |
| 2948, 2952 | *CH_3_ | 45 |
| 3000, 3003 | *CH_4_ | 45 |
| 3600-3800 | OH | 46 |
| Remaining data | Carbonate species | 43, 47, 48 |

**References**

1. Yao, Y. et al. Extraterrestrial photosynthesis by Chang’E-5 lunar soil. Joule 6, 1008–1014 (2022).

2.Wang, L. & Yu, J. Principles of photocatalysis. Interface Sci. Technol. 35, 1–52 (2023).

3.Gläser, P. et al. Illumination conditions at the lunar south pole using high resolution Digital Terrain models from LOLA. Icarus 243, 78–90 (2014).

4.Elvis, M. et al. Concentrated lunar resources: imminent implications for governance and justice. Philos. Trans. R. Soc. A 379, 2188 (2021).

5.Fan, L. et al. Research progress of lunar mineral resources and in-situ beneficiation technology. Conserv. Util. Miner. Resour. 43, 1–11 (2023).

6 Zhu, W. et al. Construction of A-FeOx/LaFeO3 hybrid microreactors for efficient photocatalytic degradation of organic pollutants. Journal of Rare Earths 43, 953-961 (2025).

7. Sun, D. et al. Heterogeneous Fenton-like removal of tri(2-chloroisopropyl) phosphate by ilmenite (FeTiO3): Kinetic, degradation mechanism and toxic assessment. Chemosphere 307, 135915 (2022).

8.Chen, X. et al. Producing substantial amount of water through reaction of lunar ilmenite and endogenous hydrogen. The Innovation 5, 100690 (2024).

9.Grönbeck, H. & Barth, C. Revealing carbon phenomena at palladium nanoparticles by analyzing the work function. J. Phys. Chem. C 123, 4360–4370 (2019).

10.Murata, Y. et al. Orientation-dependent work function of graphene on Pd(111). Appl. Phys. Lett. 97, 143114 (2010).

11.Z. Li, X. Xu, R. Ma, Z. Xie, Q. Kuang, Synergistic bulk-surface engineering of ferroelectric PbTiO3: Polarization amplification and surface carrier confinement for efficient photo-thermal coupled catalytic CO2-to-fuel conversion, Adv. Funct. Mater. 36 (2026) 2522013.

12. Dou, C. et al. Characterization of Ti-MOF derived TiOx assembled with different carboxylic acid organic ligands and differences in their CO2 photothermal catalytic reduction performance. Separation and Purification Technology, 359(3), 130863 (2025).

13. Hu, Z. et al. Vacancies induce the enhancement of CO2 photothermal reduction with water vapor via ZrO2/ZnS composite catalysts. Applied Surface Science, 686, 162209 (2025).

14. Yue, F. et al. Efficient solar-driven: Photothermal catalytic reduction of atmospheric CO2 at the gas-solid interface by CuTCPP/MXene/TiO2. Journal of Colloid and Interface Science, 677(A), 758–770 (2025).

15. Jin, M. et al. UV–visible-infrared light driven photothermal synergistic catalytic reduction of CO2 over Cs3Bi2Br9/MoS2 S-scheme photocatalyst. Journal of Colloid and Interface Science, 680(B), 235–245 (2025).

16. Liu, X. et al. Photothermal-boosted S-scheme heterojunction of α-Fe2O3@NiOx for high-selective reduction of CO2 to CO. Applied Surface Science, 671, 160747 (2024).

17. Shi, J. et al. Z-scheme ZnIn2S4/CuxO heterostructure on flexible substrate for efficient photothermal catalytic CO2 reduction. Applied Surface Science, 680, 161369 (2025).

18. Wang, J. et al. Efficient photothermal catalytic CO2 reduction over in situ construction ZnIn2S4@Ni(OH)2/NiO Z-scheme heterojunction. Chemical Engineering Journal, 479, 147719 (2024).

19. Cui, H. et al. Construction of IO-B-TiO2/In2O3 S-scheme heterojunction with photothermal effects and its highly efficient photocatalytic reduction of CO2 under full-spectrum light. Chemical Engineering Journal, 479, 147618 (2024).

20. Zhao, S. et al. High-performance photothermal catalytic CO2 reduction to CH4 and CO by ABO3 (A = La, Ce; B = Ni, Co, Fe) perovskite nanomaterials. Ceramics International, 49(12), 20907–20919 (2023).

21. Li, Q. et al. Suppressive strong metal-support interactions on ruthenium/TiO2 promote light-driven photothermal CO2 reduction with methane. Angewandte Chemie International Edition, 62(19), e202300129 (2023).

22. Zhao, S. et al. Ternary Ni–Co–Fe oxides based on Prussian blue analog for efficient photothermal catalytic CO2 reduction to CO and CH4. Applied Catalysis A: General, 655, 119109 (2023).

23. Dong, T. et al. Ru decorated TiOx nanoparticles via laser bombardment for photothermal co-catalytic CO2 hydrogenation to methane with high selectivity. Applied Catalysis B: Environmental, 326, 122176 (2023).

24. Zhang, X. et al. Tunable selectivity of photothermal CO2 reduction over composition-mediated Ni–Mo alloy catalysts. Energy Technology, 13(6), 2301505 (2025).

25. Li, Q. et al. Unveiling the roles of lattice strain by Ni exsolution on photothermal reduction of CO2 activity in BaTiO3 catalyst. Small, 21(10), 2409441 (2025).

26. Luo, Y. et al. Highly efficient and selective photothermal catalytic CO2 reduction to CH4 using the CoNi bimetallic-modified Gd2O3&Co3O4 nanocomposite. ACS Sustainable Chemistry & Engineering, 12(42), 15682–15695 (2024).

27. Jia, Z. et al. Selective photothermal reduction of CO2 to CO over Ni-nanoparticle/N-doped CeO2 nanocomposite catalysts. ACS Applied Nano Materials, 4(10), 10485–10494 (2021).

28. Hao, S. et al. Preparation of Co-Ce modified sludge-based catalyst and its application in CO2 photothermal catalytic reduction. Fuel, 397, 135415 (2025).

29. Sanz-Marco, A. et al. Continuous photothermal gas-phase CO2 hydrogenation over highly dispersed Ru-Ni on TiO2. Catalysis Today, 459, 115440 (2025).

30. Ning, S. et al. Microstructure induced thermodynamic and kinetic modulation to enhance CO2 photothermal reduction: A case of atomic-scale dispersed Co–N species anchored Co@C hybrid. ACS Catalysis, 10(8), 4726–4736 (2020).

31. Deng, B. et al. Metal-organic framework-derived Ga-Cu/CeO2 catalyst for highly efficient photothermal catalytic CO2 reduction. Applied Catalysis B: Environmental, 298, 120519 (2021).

32. Jia, J. et al. Photothermal Catalyst Engineering: Hydrogenation of Gaseous CO2 with High Activity and Tailored Selectivity. Advanced Science 4, 1700252 (2017).

33. Jia, J. et al. Visible and Near-Infrared Photothermal Catalyzed Hydrogenation of Gaseous CO2 over Nanostructured Pd@Nb2O5. Advanced Science 3, 1600189 (2016).

34. Yang, Z. et al. Size-modulated photo-thermal catalytic CO2 hydrogenation performances over Pd nanoparticles. Journal of Catalysis 424, 22–28 (2023).

35. Xu, C. et al. Photothermal Coupling Factor Achieving CO2 Reduction Based on Palladium-Nanoparticle-Loaded TiO2. ACS Catalysis 8, 6582–6593 (2018).

36. Li, N. et al. Enhanced Visible Light Photocatalytic Hydrogenation of CO2 into Methane over a Pd/Ce-TiO2 Nanocomposition. The Journal of Physical Chemistry C 121, 25795–25804 (2017).

37. Meng, X. et al. Photothermal Conversion of CO2 into CH4 with H2 over Group VIII Nanocatalysts: An Alternative Approach for Solar Fuel Production. Angewandte Chemie International Edition 53, 11478–11482 (2014).

38. Li, Y. F. et al. Cu Atoms on Nanowire Pd/HyWO3–x Bronzes Enhance the Solar Reverse Water Gas Shift Reaction. Journal of the American Chemical Society 141, 14991–14996 (2019).

39. Li, Y. F. et al. Pd@HyWO3–x Nanowires Efficiently Catalyze the CO2 Heterogeneous Reduction Reaction with a Pronounced Light Effect. ACS Applied Materials & Interfaces 11, 5610–5615 (2019).

40. Wu, D. et al. Plasmon-Assisted Photothermal Catalysis of Low-Pressure CO2 Hydrogenation to Methanol over Pd/ZnO Catalyst. ChemCatChem 11, 1598–1601 (2019).

41. Li, Q. et al. Disclosing support-size-dependent effect on ambient light-driven photothermal CO₂ hydrogenation over nickel/titanium dioxide. Angew. Chem. 136, e202318166 (2024).

42. Tan, T. H. et al. Unlocking the potential of the formate pathway in the photo-assisted Sabatier reaction. Nat. Catal. 3, 1034–1043 (2020).

43. Wan, X. et al. A nonmetallic plasmonic catalyst for photothermal CO₂ flow conversion with high activity, selectivity and durability. Nat. Commun. 15, 1273 (2024).

44. Ji, G. et al. In-situ exsolved ultrafine Ni nanoparticles from CeZrNiO₂ solid solution for efficient photothermal catalytic CO₂ reduction by CH₄. Adv. Powder Mater. 3, 100188 (2024).

45. Heterophase junction engineering: Enhanced photo-thermal synergistic catalytic performance of CO₂ reduction over 1T/2H-MoS₂. J. Colloid Interface Sci. 652, 936–944 (2023).

46. Liang, L. et al. Infrared light-driven CO₂ overall splitting at room temperature. Joule 2, 1004–1016 (2018).

47. Guo, C. et al. Reinforcing the efficiency of photothermal catalytic CO₂ methanation through integration of Ru nanoparticles with photothermal MnCo₂O₄ nanosheets. ACS Nano 17, 23761–23771 (2023).
